# Selective activation of FZD2 and FZD7 reveals non-redundant function during mesoderm differentiation

**DOI:** 10.1016/j.stemcr.2024.102391

**Published:** 2025-01-16

**Authors:** Rony Chidiac, Andy Yang, Elli Kubarakos, Nicholas Mikolajewicz, Hong Han, Maira P. Almeida, Pierre E. Thibeault, Sichun Lin, Graham MacLeod, Jean-Philippe Gratton, Jason Moffat, Stephane Angers

**Affiliations:** 1 Donnelly Centre for Cellular and Biomolecular Research, University of Toronto, Toronto, ON, Canada; 2 Leslie Dan Faculty of Pharmacy, University of Toronto, Toronto, ON, Canada; 3 Program in Genetics and Genome Biology, The Hospital for Sick Kids, Toronto, ON, Canada; 4 Department of Pharmacology and Physiology, Faculty of Medicine, Université de Montréal, Montreal, QC, Canada; 5 Department of Molecular Genetics, University of Toronto, Toronto, ON, Canada; 6 Department of Biochemistry, University of Toronto, Toronto, ON, Canada

**Keywords:** Wnt signaling, human pluripotent stem cells, mesoderm specification, directed differentiation, Wnt agonists, Frizzled receptors

## Abstract

During gastrulation, Wnt-β-catenin signaling dictates lineage bifurcation generating different mesoderm cell types. However, the specific role of Wnt receptors in mesoderm specification remains elusive. Using selective Frizzled (FZD) and LRP5/6 antibody-based agonists, we examined FZD receptors’ function during directed mesoderm differentiation of human pluripotent stem cells (hPSCs). We found that FZD2 and FZD7 receptors are expressed at the membrane of hPSCs and that their activation triggers β-catenin signaling with different kinetics, thereby influencing mesoderm patterning choices. Specifically, FZD7 activation enhances both paraxial and lateral mesoderm differentiation, whereas FZD2 activation favors paraxial mesoderm. Mechanistically, FZD2 activation promotes sustained Wnt-β-catenin levels, guiding hPSCs differentiation toward paraxial mesoderm, while blocking lateral mesoderm. In contrast, FZD7 activation kinetics display similar initial activation but more dampening of β-catenin signaling, permitting lateral mesoderm induction in addition to paraxial mesoderm specification. Our findings reveal non-redundant roles for FZD2 and FZD7 in mesoderm specification, offering leverage for precise directed differentiation outcomes.

## Highlights

• FZD2 and FZD7 are the most highly expressed FZD receptors in hPSCs
• FZD7 but not FZD2 activation directs lateral mesoderm differentiation
• FZD2 and FZD7 activation trigger distinct Wnt-β-catenin signaling kinetics
• Selective activation of FZD2 and FZD7 activation dictates the mesodermal fate

## Graphical abstract

**Figure:**
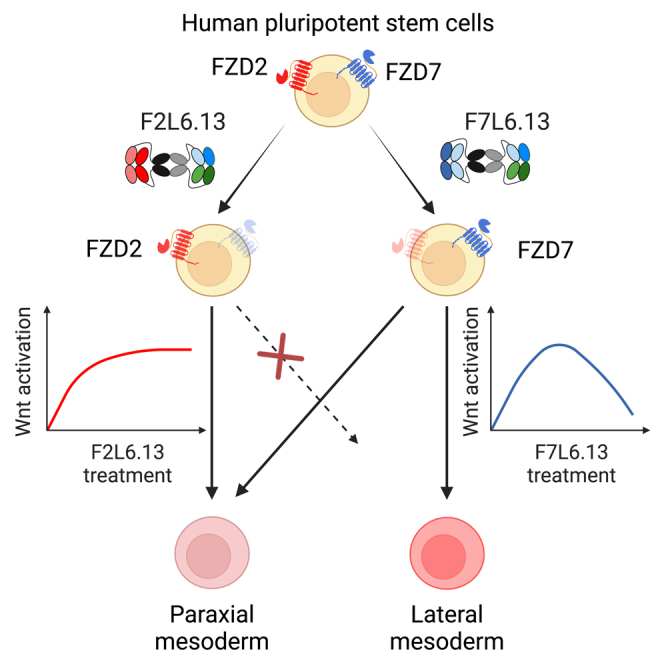

## Teaser

Chidiac and colleagues characterize Wnt receptor roles in mesoderm specification. They showed that the activation of FZD2 and FZD7, the only receptors expressed at the hPSC membrane, drives β-catenin signaling with unique kinetics. FZD7 activation is transient promoting paraxial and lateral mesoderm, while FZD2 activation is sustained favoring paraxial mesoderm. These findings underscore their distinct, non-redundant roles in mesoderm patterning.

## Introduction

During embryonic development, secreted growth factors govern the differentiation of human pluripotent stem cells (hPSCs) into specific cell types (Fowler et al., 2020). The magnitude and duration of intracellular signaling determine cell fate commitment. hPSCs are a tractable *in vitro* model to study how these signals influence cell differentiation. However, current protocols often result in heterogeneous cell types, limiting their therapeutic potential (Cohen and Melton, 2011; Fowler et al., 2020). A refined understanding of the signaling mechanisms underlying cellular differentiation will lead to more homogenous and functional cell populations.

During gastrulation, hPSCs differentiate into three germ layers (Solnica-Krezel and Sepich, 2012), a process initiated by the formation of a primitive streak (PS), coinciding with the expression of the early pan-mesodermal marker gene, *TBXT* (encoding the BRACHYURY protein) (Kispert and Herrmann, 1994; Murry and Keller, 2008). Different anterior-posterior regions of the PS generate distinct mesodermal derivatives *in vitro* and *in vivo* (Fowler et al., 2020). Current mesoderm differentiation protocols use a combination of bone morphogenetic protein 4 (BMP4), fibroblast growth factor 2 (FGF2), Activin-A/NODAL, and Wnt signaling (Ang et al., 2022; Loh et al., 2016). NODAL and BMP4 signaling gradients establish the anterior-posterior axis of the PS whereas FGF and Wnt signaling are active throughout the PS at all stages (Kattman et al., 2011; Loh et al., 2014; Sumi et al., 2008). Wnt-β-catenin signaling is known to modulate the differentiation of hPSCs (Chidiac and Angers, 2023). Wnt proteins are required to differentiate hPSCs into mesendoderm since inhibition of Wnt-β-catenin signaling blocks PS induction and any downstream derivatives (Rao et al., 2016). After the formation of the PS, sustained BMP4 signaling activation and Wnt signaling suppression promote the development of lateral mesoderm (Ang et al., 2022; Loh et al., 2016; Rao et al., 2016). In contrast, prolonged Wnt signaling activation leads to the formation of the paraxial mesoderm (Loh et al., 2016; Tani et al., 2020). The paraxial mesoderm gives rise to somites that produce the bone, cartilage, skeletal muscle, and dorsal dermis whereas lateral mesoderm generates limb bud mesoderm, cardiac mesoderm, and blood (Loh et al., 2016).

Wnt-β-catenin signaling is initiated upon Wnt ligands binding to Frizzled (FZD) receptors and low-density lipoprotein receptor-related proteins 5 and 6 (LRP5/6) resulting in the accumulation and nuclear translocation of β-catenin (Steinhart and Angers, 2018). In stem and progenitor cells, nuclear β-catenin regulates a broad spectrum of context-dependent Wnt target genes implicated in cell fate specification and differentiation (Chalamalasetty et al., 2011; Huggins et al., 2017; Yamaguchi et al., 1999). In vertebrates, 19 Wnt ligands can bind and activate 10 different FZDs and LRP5/6 co-receptors (Steinhart and Angers, 2018). The function of individual FZDs during the differentiation of hPSCs remains unknown due to a paucity of available reagents to study the expression and selective activation of individual FZD receptors. Most hPSC-directed differentiation protocols often use glycogen synthase kinase-3 α/β inhibitors (GSK3i; e.g., CHIR99021) to activate Wnt-β-catenin signaling (Tan et al., 2013); however, the pleiotropic roles of this kinase could lead to off-target effects. Recent protein engineering advances have enabled the development of antibody-based FZD and LRP5/6 agonists (FLAgs) that can cluster and activate one or multiple FZDs and LRP co-receptors with complete specificity and high efficiency (Chen et al., 2020; Janda et al., 2017; Tao et al., 2019).

Here, we used tetravalent FLAg antibodies (Tao et al., 2019) to precisely dissect the dynamics of Wnt-FZD signaling circuits in individual progenitor cells during mesoderm specification. We found that both FZD2 and FZD7 are expressed in hPSCs but differences in β-catenin activation kinetics triggered by FZD2 and FZD7 lead to distinct gene expression programs that affect the mesoderm differentiation outcome. Whereas activation of FZD7 promotes paraxial and lateral mesoderm formation, FZD2 stimulation preferentially induces paraxial mesoderm specification. Our results show that FZD2 activation leads to longer and sustained Wnt-β-catenin activation when compared to FZD7 activation. These findings demonstrate that individual FZD receptors activate Wnt-β-catenin signaling with different kinetics thereby influencing cell lineage commitment during the differentiation of hPSCs into mesoderm.

## Results

### FZD2 and FZD7 receptors are required for Wnt signaling during PS induction

To examine Wnt signaling activity in hPSCs, we generated an H1 iCas9 *AXIN2*-Citrine reporter human embryonic stem cell (hESC) line by replacing the first exon of *AXIN2* (a generic Wnt-β-catenin target gene) with a cDNA coding for histone H2B fused to the Citrine fluorescent protein into H1 hESCs that were previously engineered to express doxycycline-inducible Cas9 (Figure S1A). Upon clonal selection and expansion, cells maintained expression of pluripotency markers such as OCT4 and SOX2 (Figure S1B). Validating the reporter line, treatment with the GSK3i CHIR99021 led to a significant increase in Citrine intensity and BRACHYURY expression confirming the activation of Wnt-β-catenin signaling and induction of the PS, respectively (Figures S1C and S1D).

**Figure 1:**
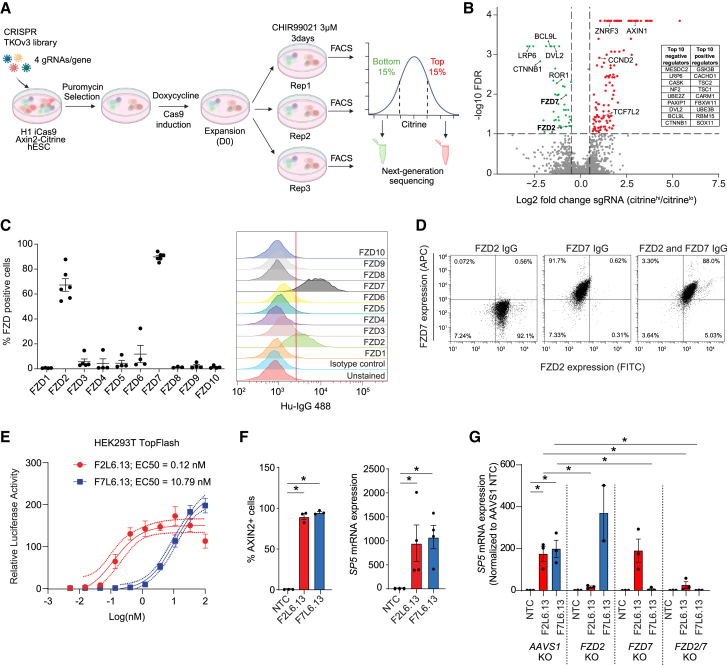
FZD2 and FZD7 are required for Wnt activation and primitive streak induction in hESCs (A) Schematic representing a genome-wide CRISPR-Cas9 in H1 *AXIN2*-citrine reporter cell line treated with CHIR99021 for three days. (B) Volcano plot of gene enrichment in the top versus bottom 15% fractions from the CRISPR screen (cutoff; log2 fold change above +/− 0.5, −log10 FDR <0.1). Green and red dots correspond to significantly enriched negative and positive regulators, respectively. Table lists the top 10 hits. Highlighted dots represent selected Wnt-related hits. (C) FZDs expression profiling in H1 hESCs by flow cytometry using specific FZDs IgG antibodies (*n* = 4–6 independent experiments). (D) Co-expression of FZD2 and FZD7 in H1 hESCs. Representative plots of three independent experiments. (E) Dose-response curve for the activation of LEF/TCF reporter gene in HEK293T cells treated with F2L6.13 or F7L6.13 (*n* = 2 independent experiments). (F) (Left) Flow cytometry analysis of the percentage of AXIN2-positive cells after one day of treatment with 30 nM of F2L6.13 or F7L6.13 using the H1 *AXIN2*-citrine reporter line (*n* = 3 independent experiments). (Right) RT-qPCR of *SP5* mRNA expression in H1 hESC treated with 30 nM of F2L6.13 and F7L6.13 for 24 h (*n* = 4 independent experiments). (G) RT-qPCR of *SP5* mRNA expression in H1 hESC treated with 30 nM of F2L6.13 and F7L6.13 for 24 h in FZD2 and FZD7 KO hESC lines (*n* = 3 independent experiments). Data are represented as mean ± SEM. Statistical analysis was performed using a two-tailed t test or one-way ANOVA followed by Tukey’s *post hoc* test. ^∗^*p* ≤ 0.05 was considered significant.

To identify regulators of Wnt-β-catenin signaling during PS formation, we performed a genome-wide CRISPR screen in the reporter H1 line treated with CHIR99021 to induce PS fate (Figure 1A). To do this, cells were first transduced with the human Toronto KnockOut library version 3 (TKOv3), selected with puromycin, and then Cas9 expression was induced to generate a population of knockout (KO) cells. Subsequently, cells were treated with CHIR99021 for three days and sorted to isolate the top and bottom 15% citrine-expressing cells (higher and lower amounts of β-catenin signaling, respectively). Next-generation sequencing was performed to quantify the relative single guide RNA (sgRNA) abundance in these two fractions (Figures 1A and 1B; Table S1). Multiple known negative regulators of Wnt-β-catenin signaling such as *TCF7L2*, *AXIN1*, *ZNRF3*, and *GSK3B* and positive regulators, such as *LRP6*, *DVL2*, *CTNNB1*, *BCL9L*, *FZD2*, and *FZD7*, were significantly enriched in the screen (Figures 1B and S1E). Supporting these results, Kyoto Encyclopedia of Genes and Genomes (KEGG) pathway enrichment showed that Wnt signaling was among the top five enriched pathways (Figure S1F). The inhibition of GSK3α/β using CHIR99021 initiates Wnt signaling downstream of FZD receptors, yet two (FZD2 and FZD7) of the ten human FZD receptors were identified as positive regulators of Wnt signaling during hESCs differentiation toward PS indicating a putative contribution of Wnt proteins-mediated signaling under these conditions (Figure 1B). To validate the screen hits, we knocked out *FZD2*, *FZD7*, and *CTNNB1* using independent sgRNAs and observed a reduction in Citrine levels three days post-CHIR99021 treatment (Figure S1G).

Next, we investigated the cell surface expression of different FZDs using antibodies that were designed to specifically bind to each of the 10 FZD receptors (Pavlovic et al., 2018; Steinhart et al., 2017). We found that FZD2 and FZD7 were highly expressed at the cell membrane of hPSCs in both H1 and WTC11 lines (Figures 1C, 1D, and S1H). Overall, these results suggest that the two main FZD receptors expressed in hPSCs, FZD2 and FZD7, have non-redundant functions during Wnt activation in hPSCs and PS formation.

### Antibody agonists specifically bind and activate FZD2 and FZD7 receptors in hESCs

We recently introduced a novel antibody modality platform enabling the selective activation of one or more FZD and LRP5/6 receptors (Chidiac et al., 2021; Tao et al., 2019). These FLAgs fully mimic the function of Wnt proteins both *in vitro* and *in vivo* (Chidiac et al., 2021; Hu et al., 2022; Nabhan et al., 2023; Tao et al., 2019; Yang et al., 2024). Leveraging this platform, we first compared FLAgs targeting the FZD2:LRP6 (F2L6.13) and FZD7:LRP6 (F7L6.13) receptor complexes (Tao et al., 2019) for their ability to activate β-catenin signaling. Treatment of HEK293T cells expressing the β-catenin-activated reporter (pBAR) (Biechele and Moon, 2008), which monitors lymphoid enhancer factor/T cell factor (LEF/TCF)-mediated transcription, showed that F2L6.13 (half maximal effective concentration (EC50) = 0.12 nM) is more potent than F7L6.13 (EC50 = 10.79 nM) (Figure 1E). To study the role of FZD2 and FZD7 in hPSC differentiation, we used 30 nM of F2L6.13 and F7L6.13 since near-maximal levels of Wnt-β-catenin signaling were observed at this dose (Figure 1E). As expected, treatment of H1 hESCs with 30 nM of F2L6.13 and F7L6.13 for 24 h increased *SP5* mRNA levels and AXIN2-Citrine expression to comparable levels (Figure 1F). To confirm the specificity of the agonists, we used CRISPR-Cas9 gene editing to generate clonal FZD2 and/or FZD7 KOs in H1 cells and confirmed the KO efficiency by sequencing (data not shown) and flow cytometry (Figure S1I). These KO clones remain pluripotent as shown by the retention of OCT4 expression (Figure S1J). Confirming the specificity of the antibody agonists, F2L6.13- or F7L6.13-mediated activation of β-catenin signaling was blunted in FZD2 and FZD7 KO lines, respectively (Figure 1G). Deletion of each individual FZD did not affect the expression or the activity of the other receptor (Figures 1G and S1I). We conclude that F2L6.13 and F7L6.13 specifically and efficiently activate β-catenin signaling in hPSCs through the respective engagement of FZD2:LRP6 and FZD7:LRP6 receptor complexes.

### Activation of FZD7 but not FZD2 receptor induces lateral mesoderm

**Figure 2:**
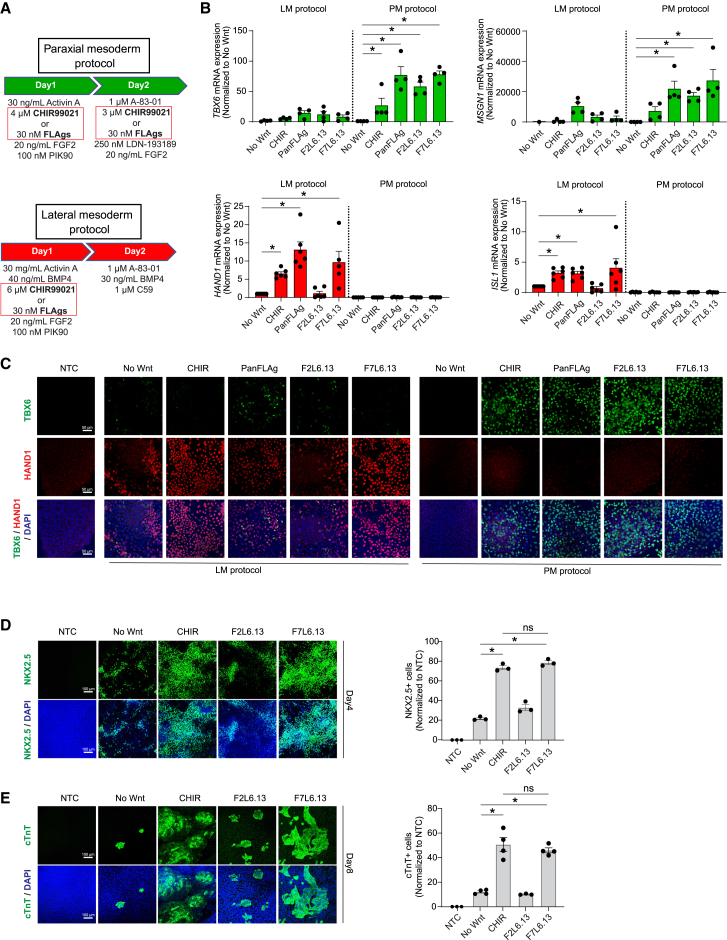
FZD2 and FZD7 activation promote distinct bifurcation choices into paraxial or lateral mesoderm (A) Paraxial versus lateral mesoderm differentiation protocol adapted from Loh et al., 2016. In each differentiation protocol, CHIR99021 treatment was replaced by 30 nM of isotype control, PanFLAg, F2L6.13, or F7L6.13 treatment. (B) RT-qPCR of hESC-derived paraxial mesoderm markers (*TBX6* and *MSGN1*) or lateral mesoderm markers (*HAND1* and *ISL1*) at day 2 of differentiation (*n* = 4–5 independent differentiation experiments). (C) TBX6 (green) and HAND1 (red) immunostaining staining in paraxial versus lateral mesoderm differentiation protocol in H1 hESCs. Images are representative of three independent differentiation experiments. (D) NKX2.5 immunostaining staining (left) and flow cytometry quantification of NKX2.5 expression (right) of hESC-derived cardiac mesoderm (day 4) (*n* = 3 independent differentiation experiments). Images are representative of three independent differentiation experiments. (E) cTnT immunostaining staining (left) and image quantification (right) of hESC-derived cardiomyocytes (day 8) (*n* = 3 independent differentiation experiments). Images are representative of three independent differentiation experiments. Data are represented as mean ± SEM. Statistical analysis was performed using a one-way ANOVA followed by Tukey’s *post hoc* test. ^∗^*p* ≤ 0.05 was considered significant.

Previously published differentiation protocols used Activin-A, BMP4, FGF2, and a GSK3i such as CHIR99021 to induce PS formation and subsequent specification of cells into different mesodermal subtypes (Loh et al., 2016). To study the role of FZD2 and FZD7 during mesoderm differentiation, we replaced CHIR99021 with (1) a FLAg that binds to FZD1, 2, 4, 5, 7, and 8 (PanFLAg); (2) F2L6.13; or (3) F7L6.13 during differentiation (Figure 2A). Similar to CHIR99021 treatment, the addition of each FLAg induced PS formation as shown by increased BRACHYURY expression and reduction of OCT4 and SOX2 after one day of differentiation (Figures S2A–S2C). We next monitored mesoderm specification under conditions where CHIR99021 was substituted by F2L6.13 or F7L6.13 treatments using a panel of paraxial and lateral mesoderm markers. Interestingly, when normalized to the absence of Wnt activation (No Wnt condition), F2L6.13 significantly increased paraxial mesoderm markers *TBX6* and *MSGN1* but not lateral/cardiac mesoderm markers *HAND1* and *ISL1* (Figures 2B, 2C, and S2D). On the other hand, PanFLAg and F7L6.13 treatment increased both paraxial and lateral mesoderm markers (Figures 2B, 2C, and S2D). This result suggests that, although early activation of FZD2 and FZD7 receptors can induce Wnt/β-catenin signaling and PS formation, subsequent activation of these two FZD receptor complexes leads to distinct mesodermal cell fates. Since only treatment with F7L6.13 promoted lateral mesoderm specification, we predicted that F2L6.13 would be unable to support cardiac mesoderm differentiation and cardiomyocyte production. As expected, F7L6.13—but not F2L6.13—treatment increased cardiac mesoderm and cardiomyocyte formation above the “No Wnt” control condition, as shown by an increase in NKX2.5 and cardiac troponin (cTnT) expression, respectively (Figures 2D and 2E). We conclude that, while stimulation of FZD7 leads to both paraxial and lateral mesoderm fate specification, FZD2 activation preferentially promotes paraxial mesoderm differentiation.

**Figure 3:**
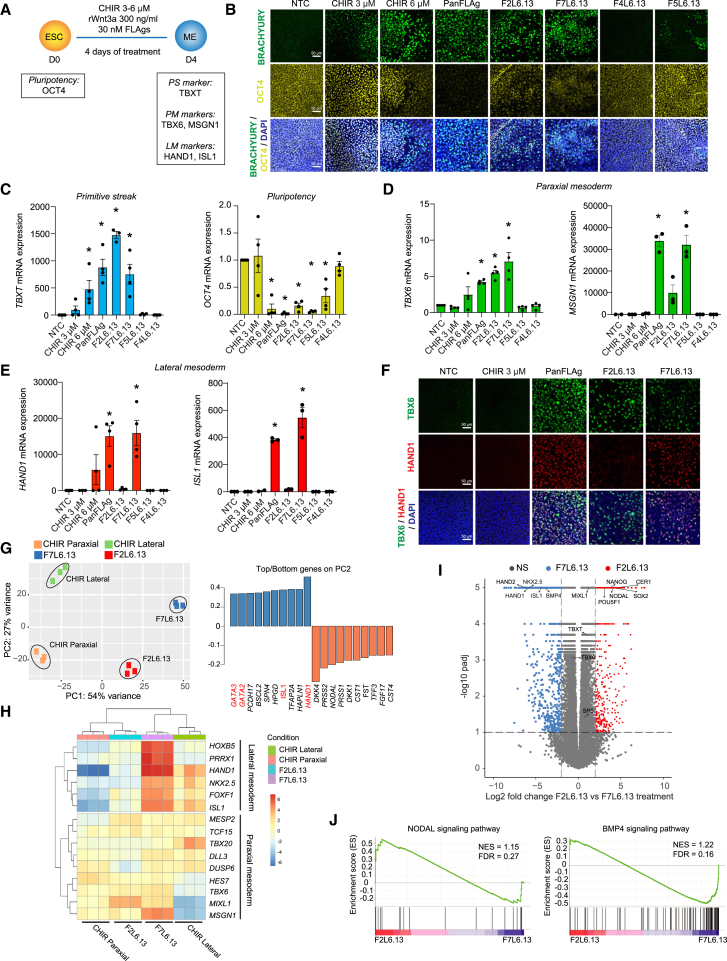
F2L6.13 and F7L6.13 differentially alter the transcriptome of hESCs (A) Schematic for mesoderm (ME) differentiation following treatment of hESCs with FLAgs or CHIR99021 without any other external signaling cues. (B and C) Immunostaining (B) and RT-qPCR (C) of the primitive streak (*TBXT*) and pluripotency (*OCT4*) markers after 4 days of treatment with the indicated doses of CHIR99021 or 30 nM of different FLAgs (*n* = 4 independent differentiation experiments). Images in (B) are representative of three independent differentiation experiments. (D and E) RT-qPCR of H1 hESC-derived paraxial mesoderm (*TBX6* and *MSGN1*) (D) and lateral mesoderm (*HAND1* and *ISL1*) (E) markers after 4 days of treatment with the indicated doses of CHIR99021 or 30 nM of different FLAgs (*n* = 3–4 independent differentiation experiments). (F) Immunostaining of TBX6 and HAND1 in CHIR99021-, PanFLAg-, F2L6.13-, or F7L6.13-treated H1 hESCs. Images are representative of three independent differentiation experiments. (G) (Left) Principal-component analysis of RNA sequencing comparing F2L6.13 and F7L6.13 treatment to the standard differentiation protocol in which CHIR99021 treatment was used to drive specifically paraxial or lateral mesoderm. (Right) Top 10 genes defining PC1 and PC2 were listed. Lateral mesoderm markers were highlighted in red. For RNA sequencing, three independent differentiation experiments per condition were performed. (H) Heatmap of hierarchical clustering of paraxial and lateral mesoderm makers. (I) Volcano plot of gene enrichment in F2L6.13- versus F7L6.13-treated H1 hESCs (30 nM, 4 days). F2L6.13-enriched genes (red circles) and F7L6.13-enriched genes (blue circles) were highlighted. (J) GSEA enrichment analysis shows NODAL and BMP4 gene sets enriched in F2L6.13 and F7L6.13 treatment, respectively. Data are represented as mean ± SEM. Statistical analysis was performed using a one-way ANOVA followed by Tukey’s *post hoc* test. ^∗^*p* ≤ 0.05 was considered significant.

To probe mechanisms and eliminate confounding effects, we assessed whether β-catenin activation using FLAg, Wnt proteins, or CHIR99021 treatments was sufficient to induce mesoderm specification independently of exogenous FGF2, ACTIVIN, and BMP4 growth factors. To do this, we treated H1 hESC with CHIR99021 (3 μM or 6 μM), rWnt3a purified protein (300 ng/mL), PanFLAg, F2L6.13, or F7L6.13 (30 nM) alone, in the absence of any exogenous factors used in standard mesoderm differentiation protocols (Figure 3A). After 4 days of PanFLAg, F2L6.13, or F7L6.13 treatment alone, we observed an increase in BRACHYURY expression to levels comparable to or higher than those after treatment with 6 μM CHIR99021 that was accompanied by a significant decrease of self-renewal markers OCT4 and SOX2 (Figures 3B and S3A–S3E). FZD4 and FZD5 receptors are not expressed in hPSCs, and therefore F4L6.13 and F5L6.13 (antibodies previously shown to activate β-catenin signaling through FZD4 and FZD5 receptors, respectively [Chidiac et al., 2021; Yang et al., 2024]) were used as negative controls. We found that stimulation of hPSCs with F2L6.13 or F7L6.13 was sufficient to induce PS (Figures 3B and 3C). We then asked if F2L6.13 or F7L6.13 treatment can derive specific mesoderm subtypes. Treating with 3 μM of CHIR99021 or 300 ng/mL of purified Wnt3a protein alone increased BRACHYURY expression slightly but did not induce any mesoderm specification as shown by the absence of induction of paraxial (*TBX6* and *MSGN1*) and lateral (*HAND1* and *ISL1*) mesoderm markers (Figures 3A–3F and S3A). This suggests that lower levels of β-catenin signaling can lead to PS but are not sufficient to generate mesoderm subtypes. Indeed, when cells were treated with higher doses (6 μM) of CHIR99021, an increase in paraxial and lateral mesoderm markers was observed. Notably, we found that treating hPSCs with 30 nM of F2L6.13 for 4 days significantly increased paraxial mesoderm markers expression (*TBX6* and *MSGN1*) but did not induce lateral mesoderm markers expression (*HAND1* and *ISL1*) (Figures 3D–3F). In contrast, PanFLAg and F7L6.13 treatment significantly increased the expression of both lateral and paraxial mesoderm markers (Figures 3D–3F). PS is known to form definitive endoderm and mesoderm. In the absence of other signaling cues used in directed differentiation protocols, F2L6.13 and F7L6.13 treatment also increased SOX17 (endoderm marker) indicating the formation of a heterogeneous mesendoderm population (Figures S3C and S3E). Overall, these results suggest that FZD2 activation drives hPSCs differentiation only toward paraxial mesoderm, whereas FZD7 activation promotes the formation of paraxial and lateral mesoderm.

### Bifurcation of paraxial versus lateral mesoderm using F2L6.13 and F7L6.13 treatment

To understand the mesodermal lineage differentiation bias between the two selective FLAgs targeting FZD2 and FZD7 receptors, we performed bulk RNA sequencing in H1 hESCs treated with either F2L6.13 or F7L6.13 for 4 days. We compared these treatments to the standard protocols used to derive paraxial or lateral mesoderm. Principal-component analysis (PCA) showed clustering of the F7L6.13-treated population with the lateral mesoderm protocol (CHIR_Lateral) and the F2L6.13-treated population with the paraxial mesoderm protocol (CHIR_Paraxial) on the principal component 2 (PC2) axis (Figure 3G). Genes involved in lateral mesoderm formation (e.g., *GATA2*, *GATA3*, *ISL1*, and *HAND1*) were among the top 10 genes contributing to the variance explained by PC2 (Figure 3G), further confirming that PC2 represents the paraxial-lateral mesoderm axis of variation. Using a curated list of paraxial and lateral mesoderm markers, we found that F7L6.13 treatment induced both mesoderm progenitor lineages whereas F2L6.13 preferentially promoted paraxial mesoderm (Figure 3H). We next performed differential gene expression analysis comparing F2L6.13 and F7L6.13 treatments (Figure 3I and Table S2). Paraxial mesoderm genes were not differentially regulated reflecting that both treatments increase paraxial mesoderm markers. However, lateral/cardiac mesoderm genes (e.g., *HAND1*, *ISL1*, and *NKX2.5*) were significantly enriched in the F7L6.13 treatment. Interestingly, *BMP4* expression was significantly increased with F7L6.13 treatment and *NODAL*, *LEFTY1*, and *CER1* were significantly upregulated with F2L6.13 treatment. These genes are known to play different/opposing roles in cell fate specification during PS formation (Kattman et al., 2011; Nostro et al., 2008; Sumi et al., 2008; Xu et al., 2014). For instance, BMP4 plays an important role in posteriorizing and lateral mesoderm formation and NODAL is increased during anterior cell specification (Martyn et al., 2018; Tsaytler et al., 2023). Accordingly, gene set enrichment analysis (GSEA) comparing the transcriptome of the two treatments revealed that BMP4 signaling was enriched in the F7L6.13-treated cells whereas NODAL signaling was enriched in the F2L7.13-treated cells (Figure 3J). Using qPCR, we validated that *BMP4* mRNA levels were highly upregulated following F7L6.13—but not F2L6.13—treatment. BMP4 signaling inhibition using LDN193189 significantly reduced the potency of F7L6.13 in deriving lateral mesoderm (Figure S3F). These results suggest that F7L6.13 mediates lateral mesoderm differentiation in part through the activation of BMP4 signaling.

### snRNA-seq reveals temporal differentiation dynamics in response to F2L6.13 and F7L6.13 treatment

**Figure 4:**
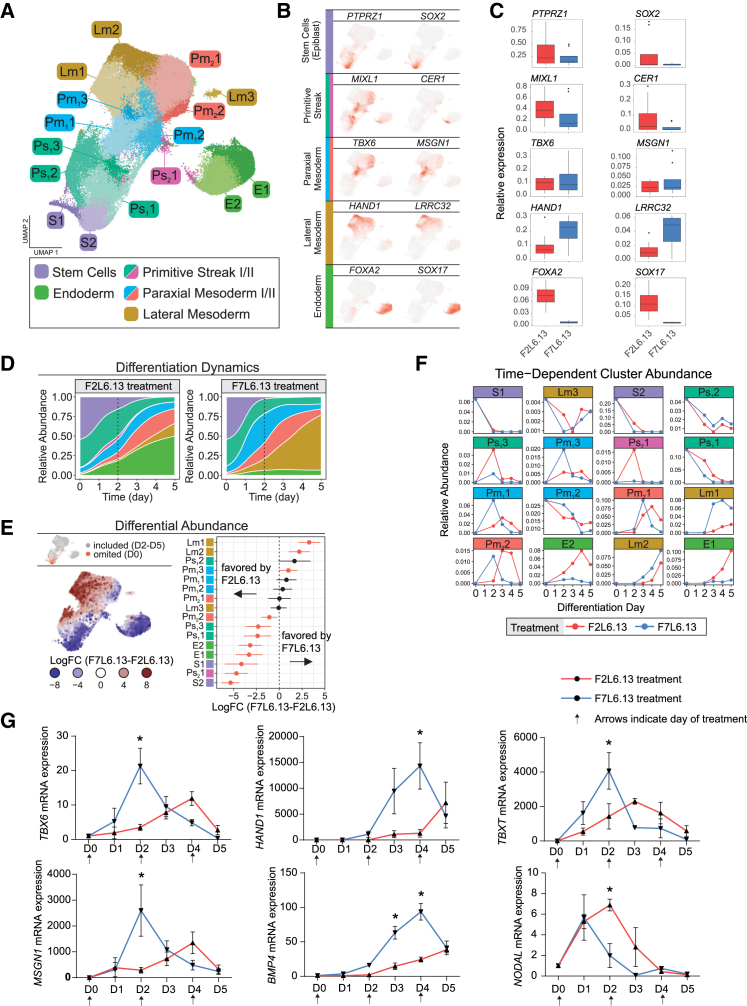
Single-nucleus transcriptome map of F2L6.13- and F7L6.13-induced mesoderm differentiation from hESCs (A) Uniform manifold approximation and projection (UMAP) of integrated single-cell populations profiled over differentiation time course. (B) UMAPs showing expression of cell-type-specific markers. (C) Boxplots of cell-type-specific markers in F2L6.13- vs. F7L6.13-treated hESCs. Data represent sample-level average expression for each day and treatment condition. D0 samples were omitted from this analysis. (D) Stream plot of relative abundances of each cell type as a function of time treated with F2L6.13 (left) or F7L6.13 (right). The same colors are used to indicate cell types as in (A). (E) Differential abundance analysis between F2L6.13- and F7L6.13-treated cells represented as UMAP (*left*) and forest plot (*right*). UMAP nodes represent cellular neighborhoods. Differentially abundant populations are indicated in red on forest plots (5% FDR, Wilcoxon test). (F) Relative abundance of cell subpopulations across five differentiation days (*n* = 2 independent differentiation experiments/time point/treatment). (G) Time course RT-qPCR analysis of stage-specific markers including primitive streak (*TBXT*), paraxial mesoderm (*TBX6* and *MSGN1*), lateral mesoderm (*HAND1* and *BMP4*), and endoderm (*NODAL*) in H1 hESCs treated with 30 nM of F2L6.13 or F7L6.13 for 5 days. Arrows indicate the day of treatment (*n* = 4 independent differentiation experiments). Data are represented as mean ± SEM. Statistical analysis was performed using a one-way ANOVA followed by Tukey’s *post hoc* test. ^∗^*p* ≤ 0.05 was considered significant.

We next used single-nucleus RNA sequencing (snRNA-seq; sci-RNA-seq3 protocol) to profile the temporal transcriptomic changes and differentiation kinetics of F2L6.13- and F7L6.13-treated H1 hESCs during days 2–5 of mesoderm induction. After stringent quality control, filtering and batch-effect correction, 81,294 nuclei were included for downstream analysis with a median of 2,097 genes detected per nucleus. We identified 16 distinct cell clusters, representing subpopulations of stem cells (S1-2), PS (Ps_1_1-3 and Ps_2_1), paraxial (Pm_1_1-3 and Pm_2_1-2) and lateral (Lm1-3) mesoderm, and endoderm (E1-2) (Figures 4A and S4A). Cell-type annotations were based on cell-type marker profiles (Figures 4B and S4B) and reference atlas-based transfer learning (see supplemental experimental procedures). Each temporal dynamic class was dissected to ensure cell-type compositions were well resolved. In line with our previous data, F7L6.13 but not F2L6.13 treatment induced increased lateral mesoderm markers *HAND1*, while F2L6.13 treatment increased endoderm markers *SOX17* and *FOXA2* (Figure 4C). We also performed differential expression analyses based on the time-dependent relative abundance similarities of each cluster and their lineage relationships (Figures 4D–4F). As expected, the abundance of stem cell states decreased over time as shown by pluripotency markers such as *SOX2* and *PTPRZ1*, whereas mesodermal and endodermal progenitors expressing specific markers such as *TBX6*, *MSGN1*, *HAND1*, and *SOX17* emerged as early as day 2 (Figure 4D). Next, we compared the relative abundance between F2L6.13- and F7L6.13-treated cells during differentiation to understand the identity of differentially regulated cell populations between the two treatments. Lateral mesoderm subpopulations (Lm1 and Lm2; defined by *HAND1*, *ISL1*, and *LRRC32* expression) were significantly enriched in F7L6.13- but not F2L6.13-treated hPSCs. We also found that the paraxial mesoderm subpopulations (Pm_1_1-3, defined by *TBX6*, *MSGN1*, *DLL1*, and *DLL3*) were equally represented in F2L6.13- and F7L6.13-treated conditions (Figures 4E, 4F, and S4C). Endoderm subpopulations (E1 and E2; defined by *SOX17* and *FOXA2*) were significantly enriched by F2L6.13 treatment. Interestingly, the endoderm lineage was preceded by a rare and distinct DKK4+/CER1+ PS subpopulation PS_2_1 that was only detected in F2L6.13- but not F7L6.13-treated cells (Figures 4E, S4B, and S4C). This is in agreement with the finding that F2L6.13 treatment significantly increased *NODAL* expression after 2 days of treatment when compared to F7L6.13 treatment (Figure 4G). In addition, pluripotent cell state markers and early PS markers were also significantly favored by F2L6.13 treatment indicating that cells treated with F2L6.13 are slower to exit the pluripotency state when compared to F7L6.13 treatment (Figures 4E, 4F, and S4C). The kinetics difference in the lateral and paraxial mesoderm formation was further validated by qPCR analysis using specific markers (Figure 4G).

Taken together, our bulk and snRNA-seq expression analyses support the conclusion that β-catenin signaling activation through selective FZD7 or FZD2 activation leads cells to adopt a paraxial mesoderm fate and that FZD7 activation additionally promotes lateral mesoderm specification.

### Kinetics of Wnt signaling activation governs mesodermal fate induction

**Figure 5:**
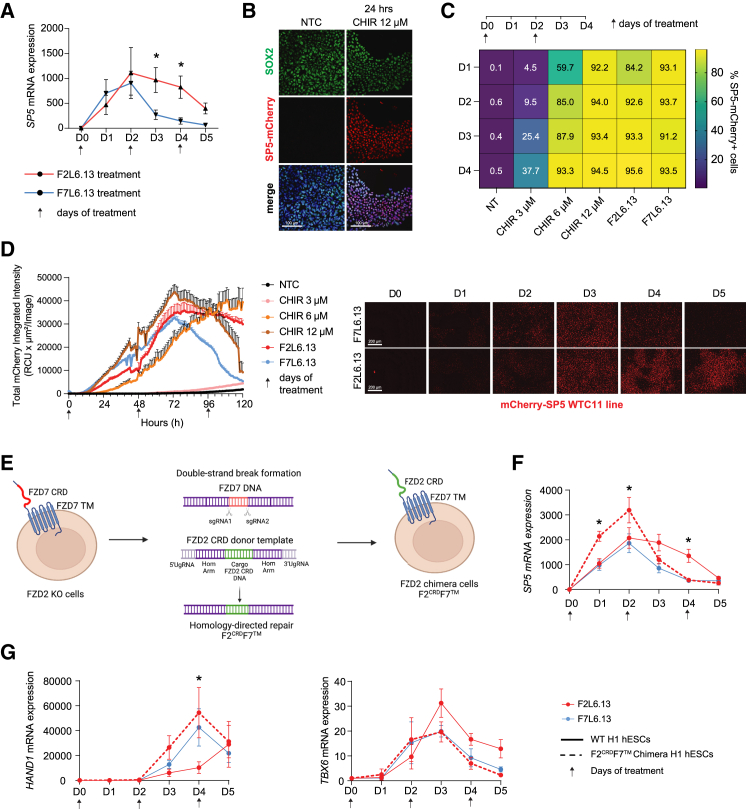
Wnt activation kinetics dictates the fate of mesoderm differentiation (A) Time course RT-qPCR analysis of *SP5* mRNA expression in H1 hESCs treated with 30 nM F2L6.13 and F7L6.13 for 5 days. Arrows indicate the day of treatment (*n* = 4 independent differentiation experiments). (B) Immunostaining against SOX2 and mCherry expression in the SP5-mCherry H1 hESC reporter line following 24 h treatment with 12 μM CHIR99021. Images are representative of two independent experiments. (C) Heatmap of flow cytometry quantification of the percentage of SP5-mCherry-positive cells in WTC SP5-mCherry reporter line treated for 5 days with different doses of CHIR99021 or 30 nM of F2L6.13 or F7L6.13. Cells were harvested for flow cytometry every day (*n* = 3 independent experiments). (D) (Left) Quantification of time-lapse imaging of SP5-mCherry fluorescence intensity over 5 days of differentiation. (Right) Representative images of SP5-mCherry cells treated with either F2L6.13 or F7L6.13 for 5 days (mean ± SEM of 9 different images per treatment). Experiment was repeated twice with similar results. (E) Diagram showing the CRISPR-Cas9 gene editing engineering of hPSC H1 chimera cell line. Using specific sgRNA, the FZD7 CRD is cut out and replaced by the FZD2 CRD domain on a FZD7 transmembrane domain creating FZD2 chimera cells (F2^CRD^F7^TM^). (F and G) Time course RT-qPCR analysis of SP5 (F) and HAND1 or TBX6 (G) mRNA expression in H1 hESCs treated with F2L6.13 and F7L6.13 or F2^CRD^F7^TM^ chimera cells treated with F2L6.13 for 5 days (*n* = 3 independent differentiation experiments). Data are represented as mean ± SEM. Statistical analysis was performed using a one-way ANOVA followed by Tukey’s *post hoc* test. ^∗^*p* ≤ 0.05 was considered significant.

A Wnt signaling gradient is known to modulate cell fate specification during gastrulation, but the contribution of different FZD receptors remains largely unexplored. Our snRNA-seq findings led us to hypothesize that the distinct kinetics of Wnt-β-catenin signaling downstream of FZD2 and FZD7 promote unique mesoderm fate specification. To test this, we examined the activation kinetics of the Wnt target gene *SP5* in response to F2L6.13 and F7L6.13 treatment (Figure 5A). Interestingly, we found that both FZD agonists caused similar steady increases in *SP5* expression during the early phase of treatment (days 0–2). However, after day 2, the activation kinetics of the Wnt-β-catenin pathway began to diverge (Figure 5A). Whereas F2L6.13 treatment resulted in sustained *SP5* expression for 4–5 days, a rapid attenuation of *SP5* expression was observed following 2 days of F7L6.13 treatment (Figure 5A). CHIR99021 treatment at a high concentration (12 μM) mirrored the kinetics of F7L6.13-mediated Wnt activation (Figure S4D).

To evaluate temporal Wnt pathway activity at a single-cell level, we generated a clonal WTC11 SP5-mCherry reporter cell line (Figure 5B). Flow cytometry analysis indicated that all cells were SP5-mCherry+ after just one day of treatment with CHIR99021 (12 μM), F2L6.13, or F7L6.13 (30 nM), and this was sustained until at least day 4 (Figure 5C). To directly monitor the activation kinetics in real time, we performed live-cell imaging experiments and measured SP5-mCherry intensity in individual cells. Consistent with our mRNA results, F2L6.13 treatment was associated with sustained SP5 upregulation compared to F7L6.13 treatment (Figure 5D). Similar results were observed in the H1 AXIN2-Citrine reporter line (Figure S4E).

Next, we investigated whether lower concentrations of F2L6.13 would enhance lateral mesoderm formation by treating cells with a maximal dose of 30 nM F2L6.13 or F7L6.13 for 2 days followed by a dose-response treatment starting on day 2. Decreasing concentration of F2L6.13 showed lower Wnt activation and increased lateral formation on day 4 (Figure S5A). Moreover, we used the Wnt-β-catenin inhibitor XAV939 to block F2L6.13-induced Wnt signaling after day 2 of differentiation. We found that lateral mesoderm formation was significantly enhanced in F2L6.13-treated cells reaching levels similar to F7L6.13-treated cells (Figure S5B). No difference in paraxial mesoderm formation was observed. These results support our findings that the differences in F2L6.13- and F7L6.13-mediated differentiation are rooted in their different ability to activate Wnt-β-catenin signaling over time, which is known to influence cell fate determination. Interestingly, when mining our transcriptomic data, we observed that FZD7 expression decreased in F7L6.13-treated cells while FZD2 expression increased in F2L6.13-treated cells, possibly offering a mechanism underlying sustained β-catenin engagement by the FZD2 agonist antibody (Figure S5C). The complete mechanisms regulating FZDs expression and activation during differentiation are therefore complex and will require further studies.

Finally, we sought to confirm that the observed differences in signaling kinetics and cell specification outcomes following F2L6.13 and F7L6.13 treatments were a property of the activated receptor and signaling network and not due to different properties of the antibodies. To do this, we generated a chimeric receptor F2^CRD^F7^TM^ in which the N-terminal cysteine-rich domain (CRD) of FZD7 is replaced by the FZD2 CRD while preserving the transmembrane domain (TM) of FZD7. We used our clonal FZD2 KO H1 lines and precisely swapped the FZD7 CRD within the FZD7 open reading frame with the coding sequence of the FZD2 CRD (Figures 5E and S5D). F2^CRD^F7^TM^ chimera line was confirmed by sequencing and flow cytometry (Figures S5D and S5E). Strikingly, F2^CRD^F7^TM^ cells treated with F2L6.13 did not show sustained Wnt-β-catenin activation observed in the H1 parental cells. In contrast, F2L6.13 treatment in the F2^CRD^F7^TM^ cells led to an early stimulation of the Wnt target genes *SP5* that was rapidly dampened, mimicking F7L6.13 stimulation in the parental cells (Figure 5F). In agreement with the observed Wnt-β-catenin activation kinetics, stimulation of hESCs expressing the F2^CRD^F7^TM^ chimeric receptor with F2L6.13 led to robust expression of *HAND1* (lateral mesoderm) and to faster dampening of *TBX6* (paraxial mesoderm), both mimicking the response obtained following FZD7 stimulation (Figure 5G).

Collectively, our data show that sustained Wnt pathway activation downstream of the FZD2 receptor promotes paraxial mesoderm and represses lateral mesoderm. In contrast, transient activation of the Wnt-β-catenin pathway through the FZD7 receptor promotes the lateral mesoderm. Therefore, our results reveal that activation of different FZD-containing receptor complexes leads to distinct Wnt-β-catenin signaling kinetics that influences cell differentiation outcomes such as mesodermal cell fate bifurcation choices.

## Discussion

Wnt-β-catenin signaling governs mesendoderm lineage specification; however, the role of individual FZD receptors in regulating hPSC differentiation is poorly defined. Here, using selective FZD antibody agonists, we precisely controlled the activation of Wnt signaling at the receptor level during mesoderm cell fate specification and uncovered that stimulation of FZD2 and FZD7 led to different differentiation outcomes. Mechanistically, we demonstrated that stimulation of FZD2 and FZD7 activates β-catenin signaling with different kinetics and transcriptional changes promoting distinct mesodermal lineage bifurcation choices.

Selective activation of FZD receptor complexes using antibody-based molecules that mimic Wnt proteins and efficiently stimulate the pathway has only recently been developed (Janda et al., 2017; Tao et al., 2019). In previous studies, we showed that a pan-acting agonist antibody that binds to FZD1, 2, 4, 5, 7, 8, and LRP6 can induce the differentiation of hPSCs into mesoderm (Tao et al., 2019), whereas a selective FZD5:LRP6 agonist was shown to pattern neural progenitors into midbrain fate (Yang et al., 2024). A FZD7:LRP6 tetravalent antibody agonist was also shown to induce mesendodermal differentiation of hESCs (Gumber et al., 2020). The access to highly selective Wnt surrogates therefore provides an emerging option for optimal spatiotemporal control of Wnt-β-catenin signaling to enhance directed differentiation of hPSCs.

Our transcriptomic data reveal that H1 hESCs express FZD2, FZD3, FZD5, and FZD7, consistent with findings from other studies (Fernandez et al., 2014). However, when examining protein levels using the selective FZD antibodies, we observed that FZD2 and FZD7 receptors are predominantly expressed at the membrane among the ten different FZDs. This discrepancy underscores the importance of assessing FZD receptor protein levels at the membrane, as they may differ from their transcriptional expression. FZD2 and FZD7 share strong sequence homology and were suggested to play redundant roles during development (Sagara et al., 1998; Yu et al., 2012). FZD7 is known to be the most abundant FZD receptor in undifferentiated hPSCs (Fernandez et al., 2014). Our genome-wide CRISPR screen designed to identify regulatory mechanisms of Wnt-β-catenin signaling during PS formation suggested a unique and non-redundant function for FZD2 and FZD7 receptors in hESCs. This also suggests that, during CHIR-driven hPSC differentiation, β-catenin signaling contributes to the upregulation and secretion of Wnt proteins that engage FZD2 and FZD7, consistent with the literature demonstrating a positive feedback mechanism where Wnt pathway activation enhances Wnt ligand expression (Nakamura et al., 2011).

Although both *AXIN2* and *SP5* are target genes of Wnt signaling, *SP5* is a more robust marker for pathway activation in stem cells and provides a more accurate measure than *AXIN2*, which shows a transient and weaker response following Wnt3a and GSK3β inhibitor treatments (Gumber et al., 2020; Huggins et al., 2017; Söderholm et al., 2023). We showed that, while the Wnt target gene *SP5* was equally upregulated 24 h and 48 h following F2L6.13 and F7L6.13 treatments in hPSCs, F7L6.13 induced a faster exit from the pluripotency state when compared to F2L6.13. This result suggests early differences in gene expression regulation following activation of FZD2 and FZD7. Whole and single-cell transcriptomic analysis further revealed that F2L6.13 and F7L6.13 elicit distinct transcriptional responses associated with mesoderm specification. FZD7 but not FZD2 activation showed strong induction of lateral/cardiac mesoderm markers such as *HAND1*, *ISL1*, *LRRC32*, *NKX2.5*, *BMP4*, *GATA2*, and *GATA3* (Loh et al., 2016). Our snRNA-seq data show that F2L6.13 treatment forms unique PS subtypes (Ps_1_3 and Ps_2_1) characterized by the expression of anterior PS genes such as *DKK4*, *CER1*, and *FOXA2*. This suggests that Ps_1_3 and Ps_2_1 cell populations might represent the anterior PS generated by F2L6.13, potentially acting as a transitional population toward paraxial mesoderm and endoderm populations while restricting lateral mesoderm formation.

Highlighting the engagement of regulatory loops supporting lineage commitment, F2L6.13 and F7L6.13 differentially trigger the expression of distinct growth factors such as *NODAL* and *BMP4*, respectively. FZD2 activation favors endoderm and paraxial mesoderm, whereas FZD7 activation promotes paraxial and lateral mesoderm, consistent with the role of NODAL in anterior PS patterning for endoderm and the role of BMP4 in mesoderm specification (Kim et al., 2015). In addition, the transcriptional hierarchy of BMP4 to Wnt to NODAL is conserved in hESCs and during gastrulation (Chhabra et al., 2019; Martyn et al., 2018). Practically, BMP4 has been harnessed to form posterior PS-like cells and induce lateral plate mesoderm patterning and cardiac mesoderm differentiation (Rojas et al., 2005; Tsaytler et al., 2023) while Activin/NODAL pathway maintains pluripotency and drives definitive endoderm specification (Brown et al., 2011; Osnato et al., 2021).

Wnt-β-catenin signaling dynamics vary dramatically in response to Wnt ligands depending on contextual factors such as the stage of differentiation or the cell type (Massey et al., 2019). A precise Wnt gradient governs the anteroposterior patterning of PS (anterior vs. posterior PS) and mesoderm specification (paraxial vs. lateral/cardiac mesoderm) (Wu et al., 2024; Zhao et al., 2019). We showed that, although activation of FZD2 and FZD7 leads to similar levels of early β-catenin target gene activation, the kinetics of activation differs between the two receptors and correlates with the observed differentiation outcomes. The robust and strong activation of Wnt signaling obtained using high doses of CHIR00921 treatment (higher than 6 μM) followed by the use of Wnt inhibitors such as the porcupine inhibitor (IWP2) is commonly used to generate cardiac mesoderm differentiation (Lian et al., 2013). We observed that FZD7 activation stimulates a strong early phase of Wnt-β-catenin activation that is inhibited on its own within 48 h similar to what is observed following treatment with 12 μM of CHIR99021. In contrast, FZD2 activation led to sustained activation kinetics compared to FZD7 activation or CHIR99021-mediated β-catenin signaling. This sustained activation of Wnt target genes is also associated with a slower emergence of paraxial mesoderm while lateral mesoderm is blocked. It has been shown that the continuous expression of *TBX6* can suppress cardiac differentiation and induce paraxial mesoderm and somite lineage specification (Sadahiro et al., 2018). *TBX6* and *MGSN1* expression slowly and continuously increase in F2L6.13-treated cells whereas F7L6.13 treatment is faster in increasing *TBX6* expression. This kinetics profile is in correlation with a slower exit of pluripotency induced by the FZD2 antibody agonist.

One important question raised by our findings is how activation of two structurally related receptors (FZD2 and FZD7) leads to distinct β-catenin activation profiles. Our results demonstrate that the kinetics of β-catenin transcriptional activity differs following activation of FZD2 and FZD7. Mechanistically how this is achieved remains to be determined but could be a result of different rates of receptor endocytosis or recycling to the plasma membrane or ligand-dependent receptor degradation leading to dampening of the response. Alternatively, these receptors could localize to different plasma membrane subdomains, interact with different effectors, or transit to separate endocytic routes influencing the extent of β-catenin stabilization. Importantly, these differences are intrinsic to the individual FZD receptors, and not a property of the ligand, since swapping the CRD of FZD7 for the CRD of FZD2 was found to change β-catenin activation kinetics in response to the FZD2 antibody agonist F2L6.13 when compared to wild-type cells.

Altogether, our work uncovered that activation of FZD2 and FZD7 in hES cells leads to distinct kinetics of ß-catenin-mediated transcriptional response that influences cell differentiation. Given the complex network of 19 Wnt and 10 FZD receptors, these differences are likely to pervasively influence lineage commitment during development and tissue homeostasis. This therefore constitutes a previously underappreciated layer of regulation in addition to the spatiotemporal control of Wnt proteins and FZD receptor expression. Whether these differences in β-catenin activation kinetics play important roles *in vivo* remains to be determined but could, as we demonstrated, be leveraged to improve directed differentiation of hPSCs into more homogenous or functional cell types.

## Experimental procedures

For additional information, see supplemental experimental procedures.

### Maintenance of hPSCs

The hPSC line H1 hESCs (male) were obtained from the WiCell Research Institute (WAe001-A). WTC11 hiPSC line (male) was obtained from the Conklin Lab at Gladstone Institutes, UCSF (UCSFi001-A). H1 hESCs and WTC11 hiPSCs were cultured on Geltrex-coated plates in a StemFlex basal medium. hPSCs were maintained and expanded using established procedures and detailed in the supplemental information.

### Cell differentiation

hPSCs were differentiated into mesoderm and downstream lineages as previously described (Loh et al., 2016). Alternatively, mesoderm differentiation was also examined in response to only Wnt activation in the absence of any external cues using specific concentrations of CHIR99021 or FLAg molecules every 2 days for 5 days total.

### Generation of cell lines

#### H1 *AXIN2*-Citrine reporter cell line

H1 iCas9 *AXIN2*-Citrine reporter hESC line was generated by replacing the first exon of *AXIN2* with a cDNA coding for histone H2B fused to the Citrine fluorescent protein into H1 hESCs that were previously engineered to express doxycycline-inducible Cas9 (see supplemental information, Table S3).

#### WTC11 SP5 reporter cell line

The pGTag-NLS-eGFP-SV40 vector (Addgene #117811) was adapted to apply the GeneWeld method (Wierson et al., 2020) for generating the SP5-2A-mCherry reporter allele using the H1 hESCs. The knockin vector contains a 2A-mCherry reporter cassette, a puromycin selection marker, and *LoxP* sites for marker removal. The vector also features universal sgRNA sites for Cas9-induced double-strand breaks, releasing the knockin sequence for integration. Electroporation was used to deliver the CRISPR components, and cells were selected with puromycin before isolating monoclonal lines, with the undifferentiated state confirmed by OCT4 and SOX2 staining (see supplemental information, Table S3).

#### F2^CRD^F7^TM^ chimera line

FZD7 CRD was replaced by FZD2 CRD using the GeneWeld CRISPR-Cas9 knockin strategy. Two sgRNAs were designed to induce double-strand breaks enabling the removal of the FZD7 CRD. A donor vector, containing an FZD2 CRD with 48 bp homology arms, was designed with silent mutations to prevent further cleavage by Cas9. H1 hESCs FZD2 KO cells were transfected with the donor vector and universal sgRNAs to facilitate the integration of FZD2 CRD on the FZD7 receptor. Cells were sorted by fluorescence-activated cell sorting (FACS), and genotyping confirmed correct integration. The undifferentiated state was validated by OCT4 and SOX2 staining (see supplemental information, Table S3).

### CRISPR-Cas9 screen

H1 iCas9 *AXIN2*-Citrine cells were infected with the TKOv3 sgRNA library (Addgene #90924) at an MOI of 0.3, with 400-fold coverage. After 48 h of puromycin selection, cells were treated with doxycycline and split into 3 biological replicates, each treated with 3 μM of CHIR99021, and then sorted by citrine expression (top and bottom 15%) using FACS. Genomic DNA was extracted, sequenced, and analyzed for sgRNA representation. Hits were ranked by false discovery rate (FDR), and KEGG pathway enrichment was performed on top and bottom 15% gene hits (see supplemental information, Table S1).

### Flow cytometry

Cells were harvested and stained with viability dyes or antibodies (Table S3) and then fixed before being run on the CytoFLEX S flow cytometer. Flow cytometry data were analyzed using the FlowJo software (see supplemental information).

### Bulk RNA sequencing and snRNA-seq analysis

See supplemental information for processing and analysis methods.

### Immunofluorescence

H1 hESCs treated with FLAgs and CHIR99021 were fixed, permeabilized, and blocked before incubation with primary antibodies (Table S3) and Alexa Fluor-labeled secondary antibodies. Cells were then imaged on a Zeiss LSM700 confocal microscope, and images were processed using ImageJ and Photoshop (see supplemental experimental procedures).

### Reverse-transcription PCR

Detailed methods, including primer sequences, can be found in the supplemental information as well as Table S3.

### Statistics

Statistical analyses were performed with the Prism 8 software (GraphPad, San Diego, CA, USA). The number of independent experiments is indicated in the figure legends. Graphs represent the mean ± SEM. Statistical significance was determined using a two-tailed Student’s t test to compare the means of two groups or a one-way ANOVA followed by *post hoc* Tukey’s multiple comparisons test to compare different groups with one variable. ^∗^*p* ≤ 0.05 was considered significant.

## Resource availability

### Lead contact

Further information and requests for resources and reagents should be directed to and will be fulfilled by the lead author, Stephane Angers (stephane.angers@utoronto.ca).

### Materials availability

Cell lines generated in this study are available from the lead contact upon request.

### Data and code availability

The accession number for the RNA-seq data reported in this paper is deposited at the Gene Expression Omnibus (GEO) Database: GSE267334 and the snRNA-seq data is deposited to Figshare: https://doi.org/10.6084/m9.figshare.27228831.v1.

## Acknowledgments

The Center for Pharmaceutical Oncology provided the support and instruments that were used in these experiments. We also thank the Temerty Faculty of Medicine flow cytometry facility for assisting us with cell sorting experiments. We would also like to thank all members of the Angers labs for helpful discussion throughout this study. This work was supported by the University of Toronto Medicine by Design program (MBDC2-2019-03 to J.M. and S.A.), which receives funding from the 10.13039/501100010785Canada First Research Excellence Fund. Figure 5E and the graphical abstract were created with Biorender.com.

## Author contributions

Conceptualization, R.C. and S.A.; methodology, R.C., A.Y., E.K., N.M., H.H., M.P.A., P.E.T., S.L., J.M., and S.A.; formal analysis, R.C., A.Y., E.K., N.M., H.H., and S.A.; investigation, R.C., A.Y., E.K., N.M., H.H., M.P.A., and G.M.; resources, J.-P.G., J.M., and S.A.; writing – original draft, R.C.; writing – review and editing, R.C., A.Y., N.M., P.E.T., G.M., and S.A.; funding acquisition and supervision, J.M. and S.A.

## Declaration of interests

S.A. is an inventor on patents for the antibodies described in the manuscript.

## Supplemental information

Document S1. Figures S1–S5 and supplemental experimental procedures

Table S1. CRISPR screen

Table S2. RNA sequencing

Table S3. Primer, gRNA, and antibody details

Document S2. Article plus supplemental information
