# Supplementary material for: Selective activation of FZD2 and FZD7 reveals non-redundant function during mesoderm differentiation
Source: Stem Cell Reports. 2025 Jan 16;20(2):102391. doi: 10.1016/j.stemcr.2024.102391 (PMC11864152; doi:10.1016/j.stemcr.2024.102391)
Supplement: Document S1. Figures S1–S5 and supplemental experimental procedures [file mmc1.pdf]

Stem Cell Reports, Volume *20*

Supplemental Information

Selective activation of FZD2 and FZD7 reveals non-redundant function during mesoderm differentiation

Rony Chidiac, Andy Yang, Elli Kubarakos, Nicholas Mikolajewicz, Hong Han, Maira P. Almeida, Pierre E. Thibeault, Sichun Lin, Graham MacLeod, Jean-Philippe Gratton, Jason Moffat, and Stephane Angers

Supplementary Figure 1

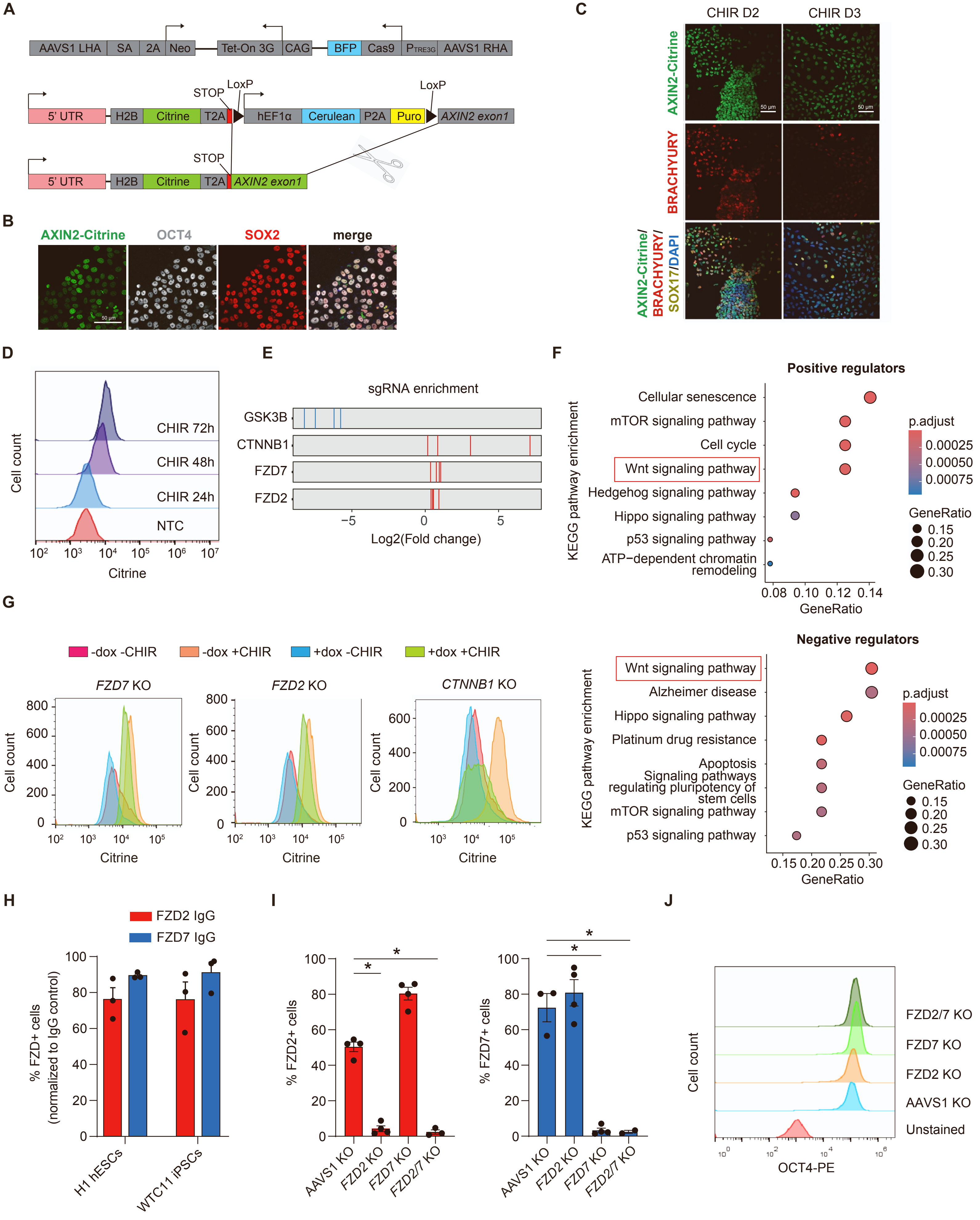

Supplementary Figure 2

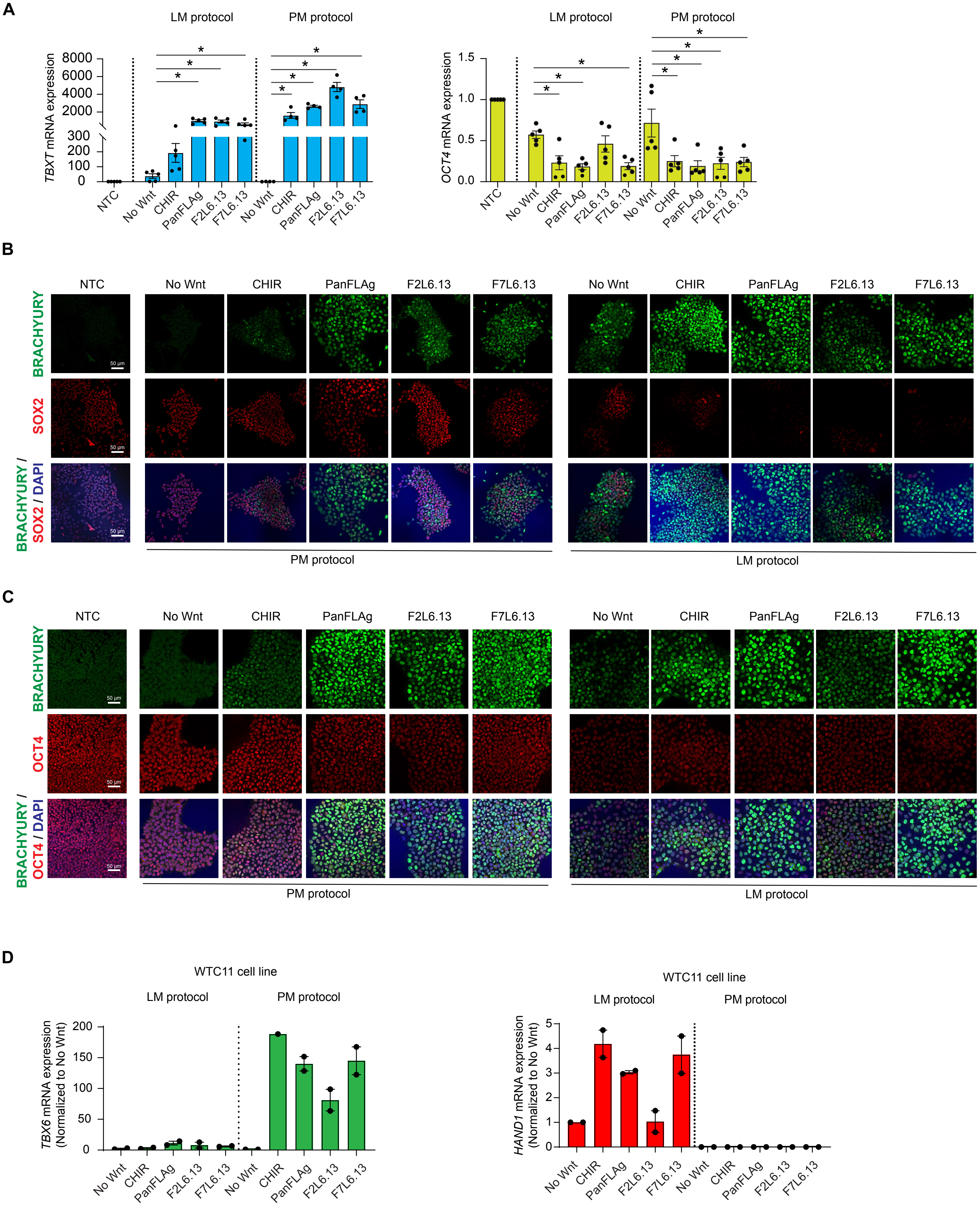

Supplementary Figure 3

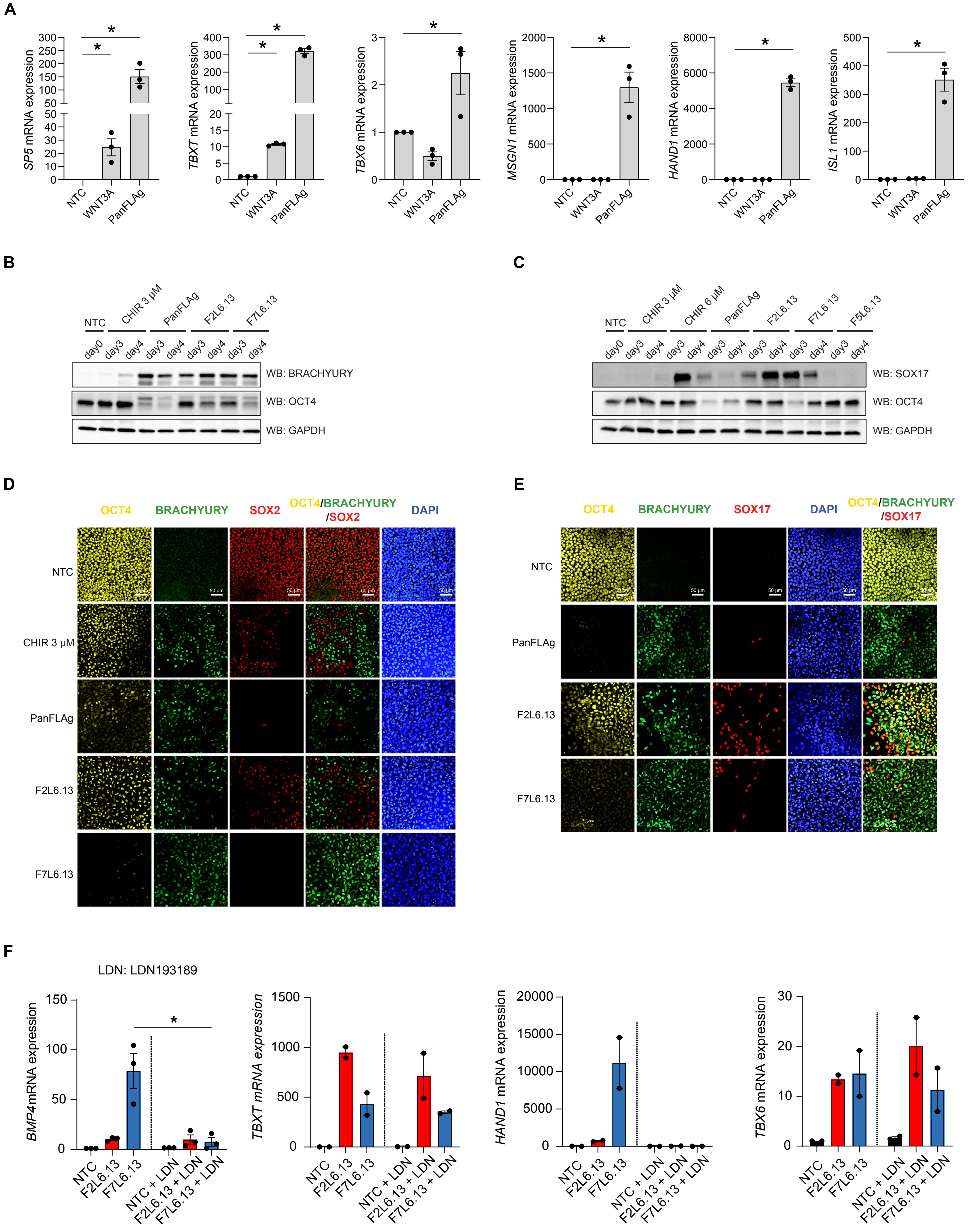

Supplementary Figure 4

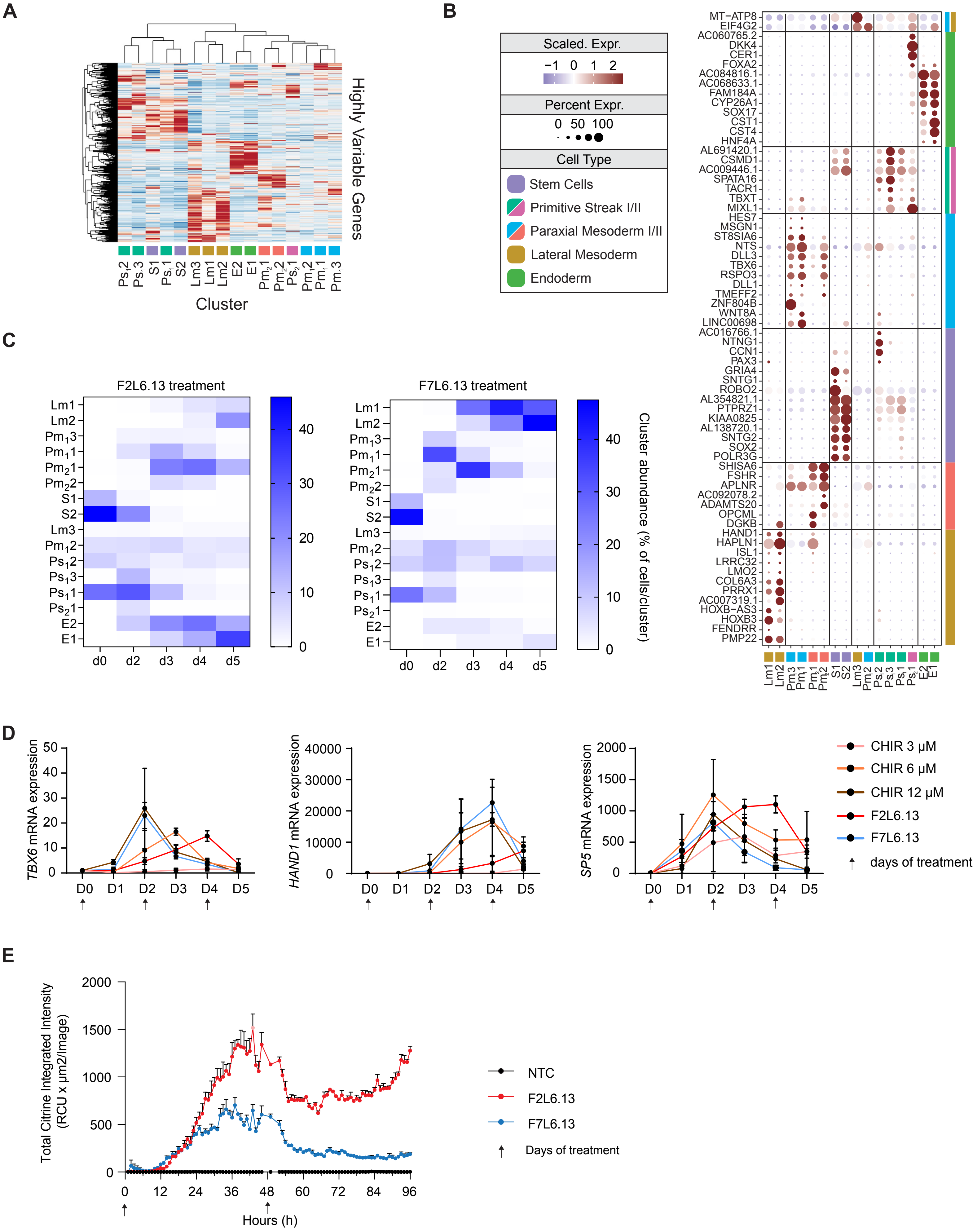

Supplementary Figure 5

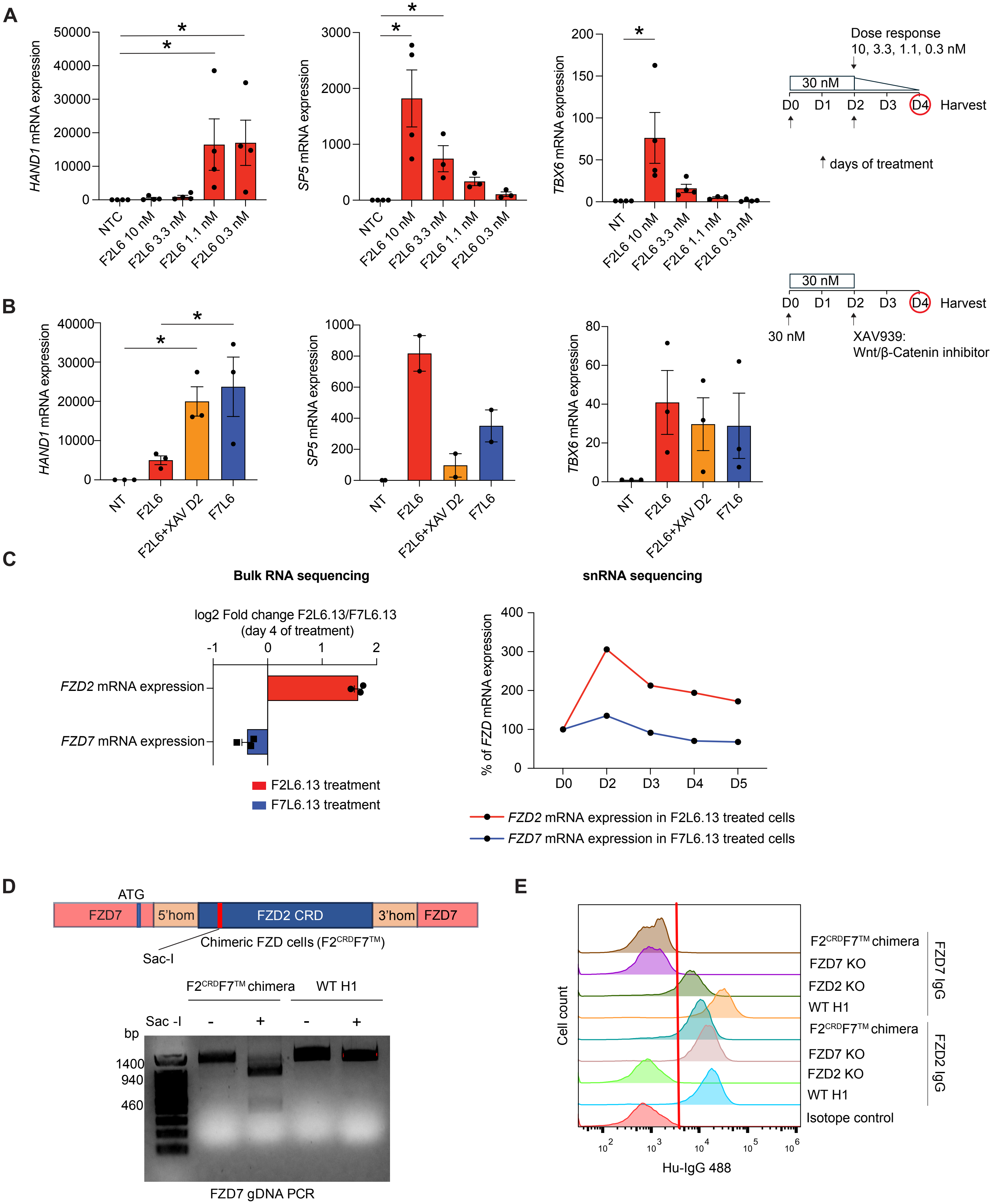

Supplementary Figures legends

Figure S1: Genome-wide CRISPR screen showing non-redundant functions for FZD2 and FZD7 during Wnt activation in hPSCs, related to Figure 1.

A) Left and right homology arms (LHA and RHA, respectively) targeting the AAVS1 safe-harbour locus allowed for integration of a Cas9-Blue fluorescent protein (BFP) cassette driven by an inducible Tet-On 3G system, coupled with a neomycin (Neo) selection cassette (cell line commissioned by the Centre for Commercialization of Regenerative Medicine, CCRM). Left and right homology arms (LHA and RHA, respectively) were cloned using restriction enzymes into a pre-constructed vector containing a nuclear H2B-tagged citrine fluorescent protein, with a constitutive hEF1α-driven cerulean fluorescent protein and puromycin (Puro) resistant cell selection cassette flanked with LoxP excision sites.

B) Immunostaining against Citrine, OCT4 and SOX2 expression in the H1 iCas9 *AXIN2*-Citrine reporter line. Images are representative of two independent experiments.

C) Immunostaining against AXIN2-Citrine, BRACHYURY and SOX17 in the H1 iCas9 *AXIN2*-Citrine cells treated with 3 μM CHIR99021 for 3-4 days. Images are representative of two independent experiments.

D) Flow cytometry profiles of H1 iCas9 *AXIN2*-citrine reporter treated with 3 μM CHIR99021 for 24-72 hrs validating the responsiveness to Wnt activation. Data are representative histogram plots from two independent experiments.

E) The individual gRNA enrichment of GSK3β, CTNNB1, FZD2 and FZD7 genes is shown.

F) Gene ontology (GO) enrichment analysis plots of negative and positive regulators identified in our CRISPR screen.

G) Flow cytometry density plots of AXIN2-Citrine expression in H1 iCas9 *AXIN2*-Citrine knockout of positive regulators *FZD2*, *FZD7*, and *CTNNB1* (βcatenin) treated with 3 μM CHIR99021 for 72 hrs. Data are representative histogram plots from two independent experiments.

H) FZDs expression profiling by flow cytometry in H1 hESCs or WTC11 iPSCs using specific FZD2 or FZD7 specific IgG antibodies. Data are presented as mean ± SEM, n = 3 independent experiments.

I) Knockout FZD2, FZD7 or both in H1 hESCs. Flow cytometry shows the expression of FZD2 and FZD7 in each KO line. Data are presented as mean ± SEM, n = 4 independent experiments. Statistical analysis was performed using one-way ANOVA followed by Tukey's post hoc test. $*P \leq 0.05$ was considered significant.

J) Flow cytometry profile of OCT4 expression showing that KO lines maintain their undifferentiated state. Data are representative histogram plots from two independent experiments.

Figure S2: Mesoderm differentiation using F2L6.13 and F7L6.13 agonists, related to figure 2.

A) RT-qPCR of primitive streak (Left, *TBXT*) or pluripotency (Right, *OCT4*) marker at day 1 of differentiation protocol adapted from Loh et al. 2016. Data are presented as mean ± SEM, n = 5 independent differentiation experiments. Statistical analysis was performed using one-way ANOVA followed by Tukey's post hoc test. $*P \leq 0.05$ was considered significant.

B, C) Immunostaining against BRACHYURY (B-C), SOX2 (B) and OCT4 (C) in paraxial versus lateral mesoderm differentiation protocol (day 1) in hESCs. Images are representative of three independent differentiation experiments.

D) RT-qPCR of WTC11-derived paraxial mesoderm markers (*TBX6*) or lateral mesoderm markers (*HAND1*) at day 2 of differentiation. Data are presented as mean ± SEM, n = 2 independent differentiation experiments.

Figure S3: Examining Wnt-mediated mesoderm differentiation using Wnt ligands, CHIR99021 or various FLAgs, related to figure 3.

A) RT-qPCR of a panel of differentiation markers in H1 hESCs after 4 days of treatment with 300 ng/ml of purified Wnt3a protein or 30 nM of PanFLAg. Data are presented as mean ± SEM, n=3 independent differentiation experiments. Statistical analysis was performed using one-way ANOVA followed by Tukey's post hoc test. $^*P \leq 0.05$ was considered significant.

B) Western blot analysis of BRACHYURY and OCT4 expression in H1 hESCs treated with CHIR99021 or FLAgs. GAPDH was used as a loading control. Representative blots of three independent experiments are shown.

C) Western blot analysis of SOX17 and OCT4 expression in H1 hESCs treated with CHIR99021 or FLAgs. GAPDH was used as a loading control. Representative blots of two independent experiments are shown.

D) Immunofluorescence of BRACHYURY, OCT4 and SOX2 in H1 hESCs treated with CHIR99021 or FLAgs for 4 days. Images are representative of three independent differentiation experiments.

E) Immunofluorescence of BRACHYURY, OCT4 and SOX17 in H1 hESCs treated with CHIR99021 or FLAgs for 4 days. Images are representative of three independent differentiation experiments.

F) RT-qPCR of *BMP4*, *TBXT*, *TBX6,* and *HAND1* mRNA expression in H1 hESC treated with 30 nM of F2L6.13 and F7L6.13 in the presence or absence of BMP4 inhibitor LDN193189 (LDN) for 4 days. Data are presented as mean ± SEM, n = 2-3 independent experiments. For *BMP4* mRNA expression (n = 3), statistical analysis was performed using one-way ANOVA followed by Tukey's post hoc test. *$P \leq 0.05$ was considered significant.

Figure S4: Single-nucleus RNA sequencing analysis and kinetic profiles of Wnt target genes in F2L6.13 and F7L6.13 treated hESCs, related to figures 4 and 5.

A) Hierarchical clustering of highly variable gene expression in each cellular subpopulation. Stem cells (S1-2), primitive streak ($Ps_1$1-3 and $Ps_2$1), paraxial ($Pm_1$1-3 and $Pm_2$1-2) and lateral (Lm1-3) mesoderm, and endoderm (E1-2).

B) Dot plot of differentially expressed gene markers. Scaled average expression and percent of cells expressing the indicated gene were indicated by dot color and size, respectively. Temporal classes with colors corresponding to the identity of each cluster.

C) Heatmap of cluster abundance measuring the percentage of cells in each cluster of F2L6.13 (Left) versus F7L6.13 (Right) treated H1 hESCs. Data are presented as means, n = 2 independent differentiation experiments/timepoint/treatment.

D) Time course gene expression of *SP5*, *TBX6*, and *HAND1* markers in hESCs treated with the indicated concentration of CHIR99021 or 30 nM of F2L6.13 and F7L6.13 for 5 days. Data are presented as mean ± SEM. Experiment was repeated twice with similar results.

E) Quantification of time-lapse imaging of AXIN2-Citrine fluorescence intensity over 4 days of differentiation (mean ± SEM of 3 different images per treatment). Experiment was repeated twice with similar results

Figure S5: Effect of modulating Wnt activation kinetics on mesoderm formation, related to figure 5.

A) Dose-response RT-qPCR analysis of *SP5*, *TBX6*, and *HAND1* markers in hESCs treated with a maximal dose of 30 nM F2L6.13 followed with different concentrations of F2L6.13 starting on day 2 and harvested at day 4 of treatment. Data are presented as mean ± SEM, n = 4 independent experiments. Statistical analysis was performed using one-way ANOVA followed by Tukey's post hoc test. *$P \leq 0.05$ was considered significant.

B) RT-qPCR analysis of *SP5*, *TBX6*, and *HAND1* markers in hESCs treated with a maximal dose of 30 nM F2L6.13 followed with 2 μM XAV939 on day 2 and harvested at day 4 of treatment. Data are presented as mean ± SEM, n = 2-3 independent experiments. For *HAND1* mRNA expression (n = 3), statistical analysis was performed using one-way ANOVA followed by Tukey's post hoc test. *$P \leq 0.05$ was considered significant.

C) *FZD2* and *FZD7* expression in bulk and snRNA sequencing data. (Right) *FZD2* and *FZD7* mRNA expression in F2L6.13 and F7L6.13-treated cells were determined by calculating the fold

change between F2L6.13 and F7L6.13 treatment. (Left) *FZD2* and *FZD7* mRNA expression is shown as a percentage relative to baseline expression at day 0.

D) Schematic of $F2^{CRD}F7^{TM}$ chimera design (top) in which the coding sequence for the FZD2 CRD replaced that of the FZD7 CRD at the *FZD7* locus. SacI is a restriction enzyme that cuts uniquely in the FZD2 CRD. PCR (bottom) using either WT H1 or $F2^{CRD}F7^{TM}$ chimeric cells in which SacI enzyme was used to cut inside the FZD2 CRD confirming the chimeric line and showing that the line is homozygous.

E) Flow cytometry showing FZD2 or FZD7 expression in WT H1 or $F2^{CRD}F7^{TM}$ chimeric cells using specific FZD2 or FZD7 IgGs. Data are representative histogram plots from two independent experiments.

Supplemental experimental procedures

Maintenance of hPSCs

H1 hESCs and WTC11 hiPSCs were cultured on Geltrex-coated (Gibco, A1413302, 1:100 in DMEM/F12; Gibco, 11320082) plates and maintained in StemFlex basal medium (Gibco, A3349401) supplemented with 1% Penicillin-Streptomycin (Gibco 10,000 U/mL, 15-140-122) at 37 °C in a humidified CO2 incubator. For maintenance, cells were passaged every 3-4 days at a 1:10 split ratio with Versene (Gibco, 15040066). For experiments, cells were washed once with PBS (Gibco, 14-190-250) and dissociated using TrypLE Select enzyme (Gibco, 12-604-021) and neutralized using 10% FBS (Gibco, 12483020) in DMEM/F12. Cells were then plated onto Geltrex-coated plates in StemFlex supplemented with 1X RevitaCell (Gibco 100X, A2644501). RevitaCell was removed the next day and the media was changed every 2 days for cell maintenance. All differentiations were conducted on authenticated and G-banded karyotyped H1 or WTC11 cells grown in feeder-free and monolayer conditions in StemFlex media and experiments were conducted within 10 passages. Daily observation under the microscope was done to monitor morphology, infection or any sort of contamination. Cells were frozen using the PSC cryopreservation kit from ThermoFisher (A2644601). hPSC lines were tested routinely for mycoplasma contamination using the MycoAlert Plus detection kit (Lonza).

Mesoderm, cardiac and cardiomyocyte cell differentiation

hPSCs were differentiated into mesoderm and downstream lineages as previously described (Loh et al. 2016). For paraxial and lateral mesoderm-directed differentiation, hPSCs were briefly washed and then differentiated into either anterior primitive streak (30 ng/mL Activin A, 4 µM

CHIR99021, 20 ng/mL FGF2, 100 nM PIK90) or mid primitive streak (30 mg/mL Activin A, 40 ng/mL BMP4, 6 µM CHIR99021, 20 ng/mL FGF2, 100 nM PIK90) for 24 hrs. Subsequently, day 1 anterior primitive streak was differentiated towards day 2 paraxial mesoderm for 24 hrs (1 µM A-83-01, 3 µM CHIR99021, 250 nM LDN-193189, 20 ng/mL FGF2). Separately, day 1 mid-primitive streak was differentiated towards day 2 lateral mesoderm for 24 hrs (1 µM A-83-01, 30 ng/mL BMP4, 1 µM C59).

For cardiac mesoderm and cardiomyocytes differentiation, day 2 lateral mesoderm was differentiated into day 4 cardiac mesoderm by treating them with 1 µM A8301, 30 ng/mL BMP4, 1 µM C59, 20 ng/mL FGF2 for 48 hrs. Subsequently, day 4 cardiac mesoderm was treated with 30 ng/mL BMP4, 1 µM XAV939, 200 µg/mL 2-phospho-ascorbic acid (Sigma) for 96 hrs to yield day 8 cardiomyocyte-containing populations. Media was changed 24 hrs for all steps.

H1 *AXIN2*-Citrine reporter cell line

The H1 iCas9 cells were generated from the H1 hESC parental line Centre for Commercialization of Regenerative Medicine (Toronto, Canada) by insertion of a vector sequence containing the inducible TRE3G-Cas9-P2A-BFP2 and constitutive Tet-On 3G expression cassettes into the safe harbor locus AAVS1 using CRISPR (Supplementary Figure S1a). H1 iCas9 cells were engineered to incorporate an H2B-tagged Citrine fluorescent protein before the first exon of the *AXIN2* locus. To do so, we used the reporter backbone TNTDNA104-pKW1-H2B-Citrine, a generous gift from the Ajamete Kaykas Lab (Novartis) which has a constitutive hEF1α-driven cerulean fluorescent protein and puromycin (Puro) resistant cell selection cassette flanked with LoxP excision sites allowing for cells to be selected for successful integration which is shown by PCR (Supplementary Figure 1A, 1B). The nickase Cas9 (nuclease deficient Cas9 D10A) and two sgRNA were used to introduce a double-stranded break upstream of the first exon of the *AXIN2* locus. Electroporation

of the sgRNA and a donor template (containing the H2B-Citrine) into H1 iCas9 cells incurred a DSB and successful insertion of the Citrine cassette in the *Axin2* locus. $1x10^6$ H1 iCas9 hESCs were electroporated with 2 µg of each *Axin2* Nickase sgRNA (Table S3) and 2 µg of the donor vector (containing the H2B-Citrine). Cells were electroporated using the Neon Transfection System (ThermoFisher), 100 µL Neon Tips (ThermoFisher) and following the electroporation parameters: 1050 V, 30 ms pulse width, 2 pulses. Electroporated cells were plated in 6-well plates with prewarmed StemFlex media supplemented with Revitacell. Following 2 days of puromycin treatment (2 µg/mL), cell colonies were picked manually and re-plated in individual 24-wells with Revitacell and expanded with Versene (Gibco) until they could be harvested and frozen for characterization. PCR and randomly integrated clones were excluded from subsequent experiments. PCR also confirmed the integration was heterozygous as a WT allele was still intact (Supplementary Figure 1D), and upon manual clonal selection and expansion, cells displayed heterogenous citrine expression.

Following clonal selection, the hEF1α-Cerulean-Puromycin cassette was excised using the Cre-LoxP system with the electroporation of 2.5 µg of Cre-mRNA (TriLink Biotechnologies, Cat. L-7211) per 500,000 cells. We confirmed that the clone had resumed puromycin sensitivity, Then, to ensure the removal of the Cerulean-Puromycin cassette and single-cell clonality, cells were sorted via fluorescence-activated cell sorting (FACS Aria III, BD BioSciences) and clones were further expanded. To assess whether pluripotency had been affected in the editing process, we characterized the undifferentiated cell state using OCT4 and SOX2 markers.

sgRNA lentivirus production and infection

HEK 293T cells were transfected at approximately 60% confluency with 5 µg of a psPAX2 packaging plasmid, 2 µg of a VSV.G enveloping plasmid and 5 µg of the intended lentiviral

construct cloned with the gene of interest (using a pLCKO backbone; addgene, 73311) in 250 µL of OptiMEM (Gibco, 31985070) reduced serum media. A 3:1 ratio of PEI to DNA was prepared separately, diluted in OptiMEM and briefly vortexed. The transfection reagent was transferred to the tube containing DNA and incubated for 20 min, then added dropwise to cell culture plates. The following day, the media was changed to regular DMEM containing 10% FBS and 1% PenStrep, and 24 hrs later that media was harvested and spun down (2000 x g for 2 minutes). The supernatant was filtered with a 0.4 µm filter and Lenti-X Concentrator (Takara Bio, 631232) was added to the media in a 1:3 volume ratio. Tubes were rotated for 2 hrs at 4 °C then spun at 1500 x g for 45 min at 4 °C. The pellets were resuspended in 250 µL of DMEM/F12, aliquoted and stored at -80 °C for long-term storage. Infection of hESCs was done in regular StemFlex media, the day after seeding. Media was changed before infection with 3-5 µL of concentrated virus. Media was changed 24 hrs later, then cells were selected with puromycin (2 µg/ml) for 2 days of selection. Cas9 editing was then induced by adding doxycycline (1.5 µg/ml) (Biobasic, DB0889) to media for 3-4 days in infected lines. Samples were Sanger sequenced using respective forward TIDE primers and INDELs and sgRNA cutting efficiency was measured by Tracking of Indels by DEcomposition (TIDE) analysis. Primers and sgRNA sequences were added to Table S3.

CRISPR Cas9 Screen

H1 iCas9 *AXIN2*-Citrine cells (passage number 75) were seeded in 15 cm plates with RevitaCell-supplemented StemFlex media. The following day cells were infected with the TKOv3 virus (71,090 sgRNAs) (addgene pooled library #90924) (Hart et al. 2017) at a MOI of 0.3 and library coverage of 400-fold. After 24 hrs, media was changed and cells were subjected to 48 hrs of puromycin selection (2 µg/mL). After splitting cells, and maintaining fold coverage, cells were treated with doxycycline (1.5 µg/mL) for 4 days. Following doxycycline selection, cells were split

into 3 biological replicates and treated with a final concentration of 3 μM of CHIR99021 for 3 days. Cells were harvested, resuspended in FACS buffer (5 mM EDTA, 25 mM HEPES, 1% FractionV BSA, in DPBS) and then stained with live/dead stain eBioscience Fixable Viability Dye eFluor 450 (Invitrogen, 65-0863-18) for 30 min on ice in the dark, fixed with 1% paraformaldehyde (PFA 32%, EMD 15714-S) for 20 min., then resuspended in FACS buffer. Samples were stored in the dark at 4 °C in FACS buffer until sorted. Cells were sorted for the top 15% and bottom 15% of citrine-expressing cells per replicate on a BD Influx and BD FACS AriaIII (BD Biosciences). Genomic DNA was extracted, amplified and barcoded with Illumina TruSeq adapters. Samples were sent for next-generation sequencing. The analysis included filtering guides with low-read counts so there was no guide misrepresentation, normalization of read counts of sorted cells versus the unsorted control and setting the default to 200 reads per sgRNA. The false discovery rate (FDR) was calculated for each individual sgRNA hit, -log10 transformed and ranked. Top 15% and bottom 15% mean counts across replicates were pooled and plotted against each other (log2 fold change "top15/bottom15"). KEGG pathway enrichment analysis was performed by taking the top list of genes from the top 15% and bottom 15% ranked list of hits with an FDR < 0.1.

Generation of a WTC11 SP5 reporter cell line

The pGTag-NLS-eGFP-SV40 vector (Addgene #117811) was adapted to apply the GeneWeld method for generating the *SP5-2A-mCherry* reporter allele using the H1 hESCs (Wierson et al. 2020). This CRISPR/Cas9 precision targeted integration strategy provides short homology arms that drive homology-mediated end joining at the cut site (Welker et al. 2021). The knock-in vector has a reporter cassette, which is composed of a self-cleaving 2A peptide from porcine teschvirus-1, a red fluorescent protein (mCherry), and a bovine growth hormone transcription termination and

polyadenylation sequence (2A-mCherry-BGHpolyA). The vector also has a selection marker consisting of a human elongation factor-1 alpha promoter followed by a puromycin resistance gene, and a rabbit beta-globulin termination and polyadenylation sequence (huEF1α-Puro-RBGpolyA). The presence of LoxP sites allows the removal of the selection marker using Cre recombinase. Flanking the knock-in sequence there are 48 bp homology arms corresponding to the genomic target site. Externally, there are universal sgRNA (UgRNA) sites for Cas9-induced double-strand breaks and consequent release of the knock-in sequence from the vector backbone and exposure of the short homology arms, which are important for integration. 5' and 3' homology arms were designed and cloned into the vector as described (Wierson et al. 2020).

To generate the reporter allele, the knock-in cassette was inserted between the last amino acid and the stop codon to guarantee the maintenance of SP5 endogenous expression. A genomic sgRNA sequence upstream of the stop codon was selected. The bases that would have been lost after the double-strand break induction were added to the end of the 5' homology arm. sgRNA and homology arm oligonucleotide sequences are listed in Table S3. Since we could not mutate the PAM without changing the amino acid, we created four silent mutations within the spacer sequence to avoid a second Cas9-induced DSB after precise integration. For SP5 targeted integration, the Neon Transfection System (Invitrogen) was used, and the electroporation mix contained 1 μg of SP5 sgRNA, 1 μg of UgRNA, 1.2 μg of Cas9 mRNA, and 1 μg of vector. 300,000 H1-hESCs were transfected at a time using 1450 V, 10 ms, and 3x pulse. Later, the polyclonal population was selected with puromycin, and the surviving cells went through the process of monoclonal line isolation followed by genotyping. Their undifferentiated state was confirmed by OCT4 and SOX2 staining.

FZD7 CRD replacement and generation of chimeric receptor

For replacing FZD7 CRD with FZD2 CRD, we created a knock-in vector compatible with the GeneWeld CRISPR/Cas9 precision targeted integration strategy[70]. Initially, two sgRNAs outside FZD7 CRD were selected for induction of DNA double-strand breaks and complete deletion of the genomic sequence between the guides. Next, we designed the donor vector to contain the FZD2 CRD, which is flanked by the 5' and 3' 48 bp homology arms corresponding to the two FZD7 genomic targeting sites. Homology arms were designed and cloned into the vector as described[71]. The bases that would have been lost after double-strand break induction were added back to both homology arms. To avoid a second Cas9-induced DSB after CRD replacement, we created six silent mutations within the sgRNA spacer sequences (Table S3). Additionally, universal sgRNA (UgRNA) sites present in the donor vector were responsible for inducing Cas9 double-strand breaks, releasing the knock-in sequence from the backbone, and exposing the short homology arms for precise integration. The Neon Transfection System (Invitrogen) was used to deliver 1 μg of FZD7 sgRNA#1, 1 μg of FZD7 sgRNA#2, 1 μg of UgRNA, 1.2 μg of Cas9 mRNA, and 1 μg of vector. H1-hESCs FZD2$^{-/-}$ were transfected using 1450 V, 10 ms, and 3x pulse. Next, cells were sorted via fluorescence-activated cell sorting (FACS Aria III, BD BioSciences) using FZD2 antibodies and clones were further expanded. Precise integration was confirmed by genotyping and the undifferentiated state was validated by OCT4 and SOX2 staining.

Flow cytometry

Cells were washed with PBS once then harvested with TrypLE and STOP solution, and washed once with PBS following pelleting (1200 rpm, 4 min, 4°C). For endogenous fluorescence, cells were stained for 30 min on ice in the dark with a fixable viability dye eFluor 450 or eFluor 780, then fixed with 1% PFA or run directly on the CytoFLEX S (Beckman Coulter) flow cytometer. For intracellular staining, cells were harvested, washed once with PBS and stained with appropriate

eFluor. Following 1% PFA fixation (10 min on ice), cells were permeabilized with 0.3% Triton (Sigma) (5 min), washed once with PBS + 0.05% Triton, blocked for 1 h in 3% bovine serum albumin (BSA) (Gibco, Cat. 15260037) + 0.05% Triton, washed once, stained with the primary (or conjugated) antibody in 3% BSA + 0.05%Triton for 1 h in the dark on ice, washed twice with PBS. Secondary staining (if necessary) was done in 3% BSA + 0.05% Triton for 30 min on ice. Following 3 washes in PBS, cells were resuspended in the FACS buffer and run on the flow cytometer. For FZD membrane staining, cells were harvested, washed once with PBS and blocked with 3% BSA for 30 min on ice. Cells were stained with 100 nM of FZD IgGs in a blocking buffer for 1 h on ice. Cells were then washed 2 times and stained with secondary antibody AF488 anti-human in addition to eFluor780 for 30 min on ice in the dark. All FLOW cytometry FCS files were exported and analyzed on FlowJo Software.

RNA sequencing

H1 hESCs were differentiated as described above in three independent experiments and RNA was extracted using TRIzol reagent (Invitrogen) following the manufacturer's instructions. Sequencing libraries were prepared and run on an Illumina NextSeq-500 instrument at the Lunenfeld-Tanenbaum Research Institute Sequencing Facility (Toronto, ON) generating 75 bp single-read FASTQ files. The transcript reads from FASTQ files were aligned to the Homo Sapiens transcriptome and quantified using Kallisto (Bray et al. 2016), following default parameters. Differential expression analysis was performed using the R Bioconductor DESEq2 package (Bray et al. 2016; Love, Huber, and Anders 2014) using default parameters with the independent filtering option disabled, with thresholds of raw fold change > 1.5 and adjusted *P*-value < 0.1. Pathway enrichment analysis using a ranked gene list was performed using GSEA software (Subramanian

et al. 2005). Heatmaps and volcano plots of differentially expressed genes were generated using R packages.

Single-nucleus RNA-Seq Experiments and Analysis

Sample processing, sci-RNA-seq3 library generation, and sequencing: Cells were harvested with 0.25% trypsin-EDTA and neuron dissociation solution (Tian et al. 2019), respectively. Cell pellets were immediately snap-frozen in liquid nitrogen and then stored at -80 °C for sci-RNA-Seq3-based single-nucleus RNA-Seq processing. Samples from all conditions were processed together to minimize batch effects. Nuclei extraction and fixation were performed as previously described (Cao et al. 2019), except for the use of a modified CST lysis buffer (Slyper et al. 2020) plus 1% SUPERase In RNase Inhibitor (AM2696). Nuclei quality was checked with DAPI and Wheat Germ Agglutinin (WGA) staining. Sci-RNA-Seq3 libraries were generated as previously described (Cao et al. 2019) using three-level combinatorial indexing. The final libraries were sequenced on an Illumina NovaSeq 6000 using the following protocol: read 1: 34 bp, read 2: 69 bp, index 1: 10 bp, index 2: 10 bp. Raw sequencing reads were first demultiplexed based on i5/i7 PCR barcodes. FASTQ files were then processed using the sci-RNA-Seq3 pipeline (Cao et al. 2019). After barcodes and UMIs were extracted from the read1 FASTQ files, read alignment was performed using the STAR short-read aligner (v2.5.2b) with the human genome (hg38) and Gencode v25 gene annotations. After removing duplicate reads based on UMI, barcode, chromosome and alignment position, reads are summarized into a count matrix of M genes x N nuclei.

Filtering**:** Raw single-cell gene count matrices were loaded into a *Seurat* object (version 4.0.4) (Butler et al. 2018; Hao et al. 2021; Satija et al. 2015; Stuart et al. 2019) and filtered to retain cells

with (*i*) 200 – 9000 recovered genes per cell, (*ii*) less than 60% mitochondrial content, and (*iii*) unmatched rate within 3 median absolute deviations of the median.

Normalization: To normalize expression values, we adopted the modeling framework previously described and implemented in the *sctransform* R Package (version 0.3.2) (Hafemeister and Satija 2019). In brief, count data were modelled by regularized negative binomial regression, using sequencing depth as a model covariate to regress out the influence of technical effects, and Pearson residuals were used as the normalized and variance stabilized biological signal for downstream analysis.

Integration: Cells from each treatment condition and differentiation day were integrated in *Seurat* using the reciprocal principal component analysis-based approach, using the top 3000 variable features.

Dimensional reduction: PCA was applied to normalized and scaled data, and the top components (accounting for 90% of variance observed in the first 50 PCs) were used for UMAP embedding using RunUMAP(max_components = 2, n_neighbours = 50, min_dist = 01, metric = cosine) in *Seurat*.

Clustering: To identify clusters, we performed Louvain clustering in *Seurat* using the *FindClusters* function (resolution = 0.6).

Differential-expression analysis: Differential expression analyses were performed using the *wilcoxauc* function implemented in the *presto* package (version 1.0.0) (Korsunsky et al. 2019). Differentially expressed genes were ranked by the area under the receiver operating characteristic (AUROC) and the top markers for each cluster were used for annotation and visualization. Cell type-specific genes were visualized using the *DotPlot* function in *Seurat*.

Cell-type annotation: Cell clusters were annotated using a combined approach if *i*) marker-based annotation (Stem cells: *PTPRZ1*, *SOX2*; Primitive Streak: *MIXL1*, *CER1*; Paraxial Mesoderm: *TBX6*, *MSGN1*; Lateral Mesoderm: *HAND1*, *LRRC32*; Endoderm: *FOXA2*, *SOX17*), and *ii*) reference atlas-based label transfer. For label transfer, developmental scRNAseq data was used as a reference atlas to annotate scRNAseq data (i.e., query) in the current study (Pijuan-Sala et al. 2019; Tyser et al. 2021). Using *Seurat's* label transfer pipeline, we identified transfer anchors for each query-reference pair using FindTransferAnchors(…, normalization.method = "SCT", reference.reudction = "pca", dims = 1:50) and then mapped the query samples onto the reference atlas using MapQuery(…, reference.reduction = "pca", reduction.model = "umap"). The resulting prediction scores were cross-validated with cluster-specific markers obtained from differential-expression analyses to inform cluster annotation. In cases where many clusters annotated to a common lineage (e.g., endoderm), they were subclassified into subtypes (e.g., E1, E2 cell types).

Cell-type composition analysis: To compare the relative abundance of each lineage across treatment conditions, counts for each lineage were tallied within each treatment condition, and divided by the total number of cells profiled for that condition.

Differential abundance analysis: To evaluate regional differential abundances of cells in UMAP space across treatment conditions, we adopted the Milo method (Dann et al. 2022). In brief, for each comparison between the FZD2 and FZD7 agonist-treated conditions, cells from each condition were first resampled to normalize cell counts, and then a KNN graph representing higher-dimensional relationships between single cells was constructed. The KNN graph was then used to define neighborhoods of cells using the refined sampling scheme (Dann et al. 2022). Finally, the number of cells belonging to each condition within each neighborhood was counted and the differential abundance was computed using a negative binomial generalized linear model.

The differential abundance estimates for each neighborhood were visualized in UMAP space, with each node representing a given neighborhood (comprised of 20-80 cells each), and the color representing the differential abundance expressed as log fold-change (FZD7/FZD2). Non-significant differentials (FDR > 0.1) were truncated at zero.

Incucyte Live imaging and quantification

mCherry SP5 reporter lines were treated with different conditions for 5 days. Images were captured over time using Incucyte S3 Live-Cell Analysis System (Essen BioScience, USA). Data were acquired from a 20x objective lens in phase contrast and a red fluorescence channel (Excitation: 585, Emission: 665, acquisition time: 2000 ms). A total of four images per well were acquired at intervals. The mCherry Integrated Intensity per image (RCU x μm^2/Image) of each condition was calculated using the Incucyte S3 Live-Cell Analysis System software. Representative images on day 4 were shown.

Immunofluorescence

H1 hESCs treated with FLAgs and CHIR99021 at different doses were washed with cold PBS and then fixed for 20 min with 4% PFA. Fixed cells were rinsed with PBS, permeabilized with 0.3% triton for 10 min. and blocked with 1% BSA for 1 h. Cells were incubated for 2 hours with primary Abs for BRACHYURY, OCT4, SOX2, SOX17, TBX6, and HAND1 in 1% BSA (Table S3). Cells were washed 3 times with PBS and then stained for secondary antibody for 1 h at room temperature using Alexa Fluor 488-labeled donkey anti-goat, Alexa Fluor 568-labeled donkey anti-mouse Ab, Alexa Fluor 568-labeled donkey anti-rabbit or Alexa Fluor 647-labeled donkey anti-mouse. Coverslips were mounted using Fluoromount (Sigma-Aldrich) and analyzed on a Zeiss LSM700

confocal microscope using a 60X oil immersion objective. Images were assembled using ImageJ and Photoshop CS6 (Adobe Systems, Mountain View, CA).

Quantitative RT-PCR

Total RNA was extracted from H1 hESCs or WTC11 cells using TRIzol reagent (Invitrogen; Thermo Fisher Scientific, Inc., Waltham, MA, USA) according to the manufacturer's protocol and quantified using a nanodrop. Total RNA (1–2 µg) was reverse-transcribed into cDNA using the High-Capacity cDNA Reverse Transcription Kit (Applied Biosystems, Cat#4368813) according to the manufacturer's protocol. qPCR was performed using the Power SYBR Green PCR Master Mix (Applied Biosystems, Cat# 4368708) using the following thermocycling conditions: 95 °C for 10 min, followed by 40 cycles of 95 °C for 15 sec., 60 °C for 1 min, and 72°C for 30 sec. Primer sequences indicated in Table S3 were used, and gene expression was evaluated by the $2^{-\Delta\Delta CT}$ method.

Luciferase reporter assay

HEK293T cells were transduced with lentivirus coding for the pBARls reporter (Biechele and Moon 2008) and with *Renilla* Luciferase as a control to generate a Wnt-βcatenin signaling reporter cell line. For luciferase assay, 2×10^5 cells were seeded in each well of 24-well plates for 24 hrs prior to stimulation. The following day, F2L6.13 or F7L6.13 protein were added, and following 16 hrs of stimulation, cells were lysed and luminescence was measured following the dual luciferase protocol (Promega) using an Envision plate reader (PerkinElmer).

Western blot

H1 ESCs were solubilized with lysis buffer (1% Nonidet P-40, 0.1% sodium dodecyl sulfate (SDS), 0.1% deoxycholic acid, 50 mM Tris (pH 7.4), 0.1 mM EGTA, 0.1 mM EDTA, 20 mM sodium fluoride (NaF), 1:500 protease inhibitors (Sigma) and 1 mM sodium orthovanadate (Na3VO4). Lysate was incubated for 30 min at 4 °C, centrifuged at 14,000 × g for 10 min, boiled in SDS sample buffer, separated by SDS-polyacrylamide gel electrophoresis, transferred onto a nitrocellulose membrane and Western blotted using the following Abs: BRACHYURY, SOX17, OCT4, GAPDH (Table S3). Ab detection was performed by a chemiluminescence-based detection system (ECL; ThermoFisher).

References

Biechele, Travis L., and Randall T. Moon. 2008. "Assaying Beta-Catenin/TCF Transcription with Beta-Catenin/TCF Transcription-Based Reporter Constructs." *Methods in Molecular Biology (Clifton, N.J.)* 468: 99–110.

Bray, Nicolas L., Harold Pimentel, Páll Melsted, and Lior Pachter. 2016. "Near-Optimal Probabilistic RNA-Seq Quantification." *Nature Biotechnology* 34 (5): 525–27.

Butler, Andrew, Paul Hoffman, Peter Smibert, Efthymia Papalexi, and Rahul Satija. 2018. "Integrating Single-Cell Transcriptomic Data across Different Conditions, Technologies, and Species." *Nature Biotechnology* 36 (5): 411–20.

Cao, Junyue, Malte Spielmann, Xiaojie Qiu, Xingfan Huang, Daniel M. Ibrahim, Andrew J. Hill, Fan Zhang, et al. 2019. "The Single-Cell Transcriptional Landscape of Mammalian Organogenesis." *Nature* 566 (7745): 496–502.

Dann, Emma, Neil C. Henderson, Sarah A. Teichmann, Michael D. Morgan, and John C. Marioni. 2022. "Differential Abundance Testing on Single-Cell Data Using k-Nearest Neighbor Graphs." *Nature Biotechnology* 40 (2): 245–53.

Hafemeister, Christoph, and Rahul Satija. 2019. "Normalization and Variance Stabilization of Single-Cell RNA-Seq Data Using Regularized Negative Binomial Regression." *Genome Biology* 20 (1): 296.

Hao, Yuhan, Stephanie Hao, Erica Andersen-Nissen, William M. Mauck 3rd, Shiwei Zheng, Andrew Butler, Maddie J. Lee, et al. 2021. "Integrated Analysis of Multimodal Single-Cell Data." *Cell* 184 (13): 3573-3587.e29.

Hart, Traver, Amy Hin Yan Tong, Katie Chan, Jolanda Van Leeuwen, Ashwin Seetharaman, Michael Aregger, Megha Chandrashekhar, et al. 2017. "Evaluation and Design of Genome-Wide CRISPR/SpCas9 Knockout Screens." *G3 (Bethesda, Md.)* 7 (8): 2719–27.

Korsunsky, Ilya, Nghia Millard, Jean Fan, Kamil Slowikowski, Fan Zhang, Kevin Wei, Yuriy Baglaenko, Michael Brenner, Po-Ru Loh, and Soumya Raychaudhuri. 2019. "Fast, Sensitive and Accurate Integration of Single-Cell Data with Harmony." *Nature Methods* 16 (12): 1289–96.

Loh, Kyle M., Angela Chen, Pang Wei Koh, Tianda Z. Deng, Rahul Sinha, Jonathan M. Tsai, Amira A. Barkal, et al. 2016. "Mapping the Pairwise Choices Leading from Pluripotency to Human Bone, Heart, and Other Mesoderm Cell Types." *Cell* 166 (2): 451–67.

Love, Michael I., Wolfgang Huber, and Simon Anders. 2014. "Moderated Estimation of Fold Change and Dispersion for RNA-Seq Data with DESeq2." *Genome Biology* 15 (12): 550.

Pijuan-Sala, Blanca, Jonathan A. Griffiths, Carolina Guibentif, Tom W. Hiscock, Wajid Jawaid, Fernando J. Calero-Nieto, Carla Mulas, et al. 2019. "A Single-Cell Molecular Map of Mouse Gastrulation and Early Organogenesis." *Nature* 566 (7745): 490–95.

Satija, Rahul, Jeffrey A. Farrell, David Gennert, Alexander F. Schier, and Aviv Regev. 2015. "Spatial Reconstruction of Single-Cell Gene Expression Data." *Nature Biotechnology* 33 (5): 495–502.

Slyper, Michal, Caroline B. M. Porter, Orr Ashenberg, Julia Waldman, Eugene Drokhlyansky, Isaac Wakiro, Christopher Smillie, et al. 2020. "A Single-Cell and Single-Nucleus RNA-Seq Toolbox for Fresh and Frozen Human Tumors." *Nature Medicine* 26 (5): 792–802.

Stuart, Tim, Andrew Butler, Paul Hoffman, Christoph Hafemeister, Efthymia Papalexi, William M. Mauck 3rd, Yuhan Hao, Marlon Stoeckius, Peter Smibert, and Rahul Satija. 2019. "Comprehensive Integration of Single-Cell Data." *Cell* 177 (7): 1888-1902.e21.

Subramanian, Aravind, Pablo Tamayo, Vamsi K. Mootha, Sayan Mukherjee, Benjamin L. Ebert, Michael A. Gillette, Amanda Paulovich, et al. 2005. "Gene Set Enrichment Analysis: A Knowledge-Based Approach for Interpreting Genome-Wide Expression Profiles." *Proceedings of the National Academy of Sciences of the United States of America* 102 (43): 15545–50.

Tian, Ruilin, Mariam A. Gachechiladze, Connor H. Ludwig, Matthew T. Laurie, Jason Y. Hong, Diane Nathaniel, Anika V. Prabhu, et al. 2019. "CRISPR Interference-Based Platform for Multimodal Genetic Screens in Human IPSC-Derived Neurons." *Neuron* 104 (2): 239-255.e12.

Tyser, Richard C. V., Elmir Mahammadov, Shota Nakanoh, Ludovic Vallier, Antonio Scialdone, and Shankar Srinivas. 2021. "Single-Cell Transcriptomic Characterization of a Gastrulating Human Embryo." *Nature* 600 (7888): 285–89.

Welker, Jordan M., Wesley A. Wierson, Maira P. Almeida, Carla M. Mann, Melanie E. Torrie, Zhitao Ming, Stephen C. Ekker, et al. 2021. "GeneWeld: Efficient Targeted Integration Directed by Short Homology in Zebrafish." *Bio-Protocol* 11 (14): e4100.

Wierson, Wesley A., Jordan M. Welker, Maira P. Almeida, Carla M. Mann, Dennis A. Webster, Melanie E. Torrie, Trevor J. Weiss, et al. 2020. "Efficient Targeted Integration Directed by Short Homology in Zebrafish and Mammalian Cells." *ELife* 9 (May). https://doi.org/10.7554/eLife.53968.
