# Supplementary material for: Selective activation of FZD2 and FZD7 reveals non-redundant function during mesoderm differentiation
Source: Stem Cell Reports. 2025 Jan 16;20(2):102391. doi: 10.1016/j.stemcr.2024.102391 (PMC11864152; doi:10.1016/j.stemcr.2024.102391)
Supplement: Document S2. Article plus supplemental information [file mmc5.pdf]

Stem Cell Reports
Article
OPEN ACCESS

Selective activation of FZD2 and FZD7 reveals non-redundant function during mesoderm differentiation

Rony Chidiac,[1] Andy Yang,[1,2] Elli Kubarakos,[1,2] Nicholas Mikolajewicz,[3] Hong Han,[3] Maira P. Almeida,[1] Pierre E. Thibeault,[1] Sichun Lin,[1] Graham MacLeod,[1] Jean-Philippe Gratton,[4] Jason Moffat,[3,5] and Stephane Angers[1,2,6,7,*]
[1]Donnelly Centre for Cellular and Biomolecular Research, University of Toronto, Toronto, ON, Canada
[2]Leslie Dan Faculty of Pharmacy, University of Toronto, Toronto, ON, Canada
[3]Program in Genetics and Genome Biology, The Hospital for Sick Kids, Toronto, ON, Canada
[4]Department of Pharmacology and Physiology, Faculty of Medicine, Université de Montréal, Montreal, QC, Canada
[5]Department of Molecular Genetics, University of Toronto, Toronto, ON, Canada
[6]Department of Biochemistry, University of Toronto, Toronto, ON, Canada
[7]Lead contact
*Correspondence: stephane.angers@utoronto.ca
https://doi.org/10.1016/j.stemcr.2024.102391

SUMMARY

During gastrulation, Wnt-β-catenin signaling dictates lineage bifurcation generating different mesoderm cell types. However, the specific role of Wnt receptors in mesoderm specification remains elusive. Using selective Frizzled (FZD) and LRP5/6 antibody-based agonists, we examined FZD receptors' function during directed mesoderm differentiation of human pluripotent stem cells (hPSCs). We found that FZD2 and FZD7 receptors are expressed at the membrane of hPSCs and that their activation triggers β-catenin signaling with different kinetics, thereby influencing mesoderm patterning choices. Specifically, FZD7 activation enhances both paraxial and lateral mesoderm differentiation, whereas FZD2 activation favors paraxial mesoderm. Mechanistically, FZD2 activation promotes sustained Wnt-β-catenin levels, guiding hPSCs differentiation toward paraxial mesoderm, while blocking lateral mesoderm. In contrast, FZD7 activation kinetics display similar initial activation but more dampening of β-catenin signaling, permitting lateral mesoderm induction in addition to paraxial mesoderm specification. Our findings reveal non-redundant roles for FZD2 and FZD7 in mesoderm specification, offering leverage for precise directed differentiation outcomes.

INTRODUCTION

During embryonic development, secreted growth factors govern the differentiation of human pluripotent stem cells (hPSCs) into specific cell types (Fowler et al., 2020). The magnitude and duration of intracellular signaling determine cell fate commitment. hPSCs are a tractable *in vitro* model to study how these signals influence cell differentiation. However, current protocols often result in heterogeneous cell types, limiting their therapeutic potential (Cohen and Melton, 2011; Fowler et al., 2020). A refined understanding of the signaling mechanisms underlying cellular differentiation will lead to more homogenous and functional cell populations.

During gastrulation, hPSCs differentiate into three germ layers (Solnica-Krezel and Sepich, 2012), a process initiated by the formation of a primitive streak (PS), coinciding with the expression of the early pan-mesodermal marker gene, *TBXT* (encoding the BRACHYURY protein) (Kispert and Herrmann, 1994; Murry and Keller, 2008). Different anterior-posterior regions of the PS generate distinct mesodermal derivatives *in vitro* and *in vivo* (Fowler et al., 2020). Current mesoderm differentiation protocols use a combination of bone morphogenetic protein 4 (BMP4), fibroblast growth factor 2 (FGF2), Activin-A/NODAL, and Wnt signaling (Ang et al., 2022; Loh et al., 2016). NODAL and BMP4 signaling gradients establish the anterior-posterior axis of the PS whereas FGF and Wnt signaling are active throughout the PS at all stages (Kattman et al., 2011; Loh et al., 2014; Sumi et al., 2008). Wnt-β-catenin signaling is known to modulate the differentiation of hPSCs (Chidiac and Angers, 2023). Wnt proteins are required to differentiate hPSCs into mesendoderm since inhibition of Wnt-β-catenin signaling blocks PS induction and any downstream derivatives (Rao et al., 2016). After the formation of the PS, sustained BMP4 signaling activation and Wnt signaling suppression promote the development of lateral mesoderm (Ang et al., 2022; Loh et al., 2016; Rao et al., 2016). In contrast, prolonged Wnt signaling activation leads to the formation of the paraxial mesoderm (Loh et al., 2016; Tani et al., 2020). The paraxial mesoderm gives rise to somites that produce the bone, cartilage, skeletal muscle, and dorsal dermis whereas lateral mesoderm generates limb bud mesoderm, cardiac mesoderm, and blood (Loh et al., 2016).

Wnt-β-catenin signaling is initiated upon Wnt ligands binding to Frizzled (FZD) receptors and low-density lipoprotein receptor-related proteins 5 and 6 (LRP5/6) resulting in the accumulation and nuclear translocation of β-catenin (Steinhart and Angers, 2018). In stem and progenitor cells,

Published by Elsevier Inc. on behalf of International Society for Stem Cell Research.
This is an open access article under the CC BY license (http://creativecommons.org/licenses/by/4.0/).

nuclear β-catenin regulates a broad spectrum of context-dependent Wnt target genes implicated in cell fate specification and differentiation (Chalamalasetty et al., 2011; Huggins et al., 2017; Yamaguchi et al., 1999). In vertebrates, 19 Wnt ligands can bind and activate 10 different FZDs and LRP5/6 co-receptors (Steinhart and Angers, 2018). The function of individual FZDs during the differentiation of hPSCs remains unknown due to a paucity of available reagents to study the expression and selective activation of individual FZD receptors. Most hPSC-directed differentiation protocols often use glycogen synthase kinase-3 α/β inhibitors (GSK3i; e.g., CHIR99021) to activate Wnt-β-catenin signaling (Tan et al., 2013); however, the pleiotropic roles of this kinase could lead to off-target effects. Recent protein engineering advances have enabled the development of antibody-based FZD and LRP5/6 agonists (FLAgs) that can cluster and activate one or multiple FZDs and LRP co-receptors with complete specificity and high efficiency (Chen et al., 2020; Janda et al., 2017; Tao et al., 2019).

Here, we used tetravalent FLAg antibodies (Tao et al., 2019) to precisely dissect the dynamics of Wnt-FZD signaling circuits in individual progenitor cells during mesoderm specification. We found that both FZD2 and FZD7 are expressed in hPSCs but differences in β-catenin activation kinetics triggered by FZD2 and FZD7 lead to distinct gene expression programs that affect the mesoderm differentiation outcome. Whereas activation of FZD7 promotes paraxial and lateral mesoderm formation, FZD2 stimulation preferentially induces paraxial mesoderm specification. Our results show that FZD2 activation leads to longer and sustained Wnt-β-catenin activation when compared to FZD7 activation. These findings demonstrate that individual FZD receptors activate Wnt-β-catenin signaling with different kinetics thereby influencing cell lineage commitment during the differentiation of hPSCs into mesoderm.

RESULTS

FZD2 and FZD7 receptors are required for Wnt signaling during PS induction

To examine Wnt signaling activity in hPSCs, we generated an H1 iCas9 *AXIN2*-Citrine reporter human embryonic stem cell (hESC) line by replacing the first exon of *AXIN2* (a generic Wnt-β-catenin target gene) with a cDNA coding for histone H2B fused to the Citrine fluorescent protein into H1 hESCs that were previously engineered to express doxycycline-inducible Cas9 (Figure S1A). Upon clonal selection and expansion, cells maintained expression of pluripotency markers such as OCT4 and SOX2 (Figure S1B). Validating the reporter line, treatment with the GSK3i CHIR99021 led to a significant increase in Citrine intensity and BRACHYURY expression confirming the activation of Wnt-β-catenin signaling and induction of the PS, respectively (Figures S1C and S1D).

To identify regulators of Wnt-β-catenin signaling during PS formation, we performed a genome-wide CRISPR screen in the reporter H1 line treated with CHIR99021 to induce PS fate (Figure 1A). To do this, cells were first transduced with the human Toronto KnockOut library version 3 (TKOv3), selected with puromycin, and then Cas9 expression was induced to generate a population of knockout (KO) cells. Subsequently, cells were treated with CHIR99021 for three days and sorted to isolate the top and bottom 15% citrine-expressing cells (higher and lower amounts of β-catenin signaling, respectively). Next-generation sequencing was performed to quantify the relative single guide RNA (sgRNA) abundance in these two fractions (Figures 1A and 1B; Table S1). Multiple known negative regulators of Wnt-β-catenin signaling such as *TCF7L2*, *AXIN1*, *ZNRF3*, and *GSK3B* and positive regulators, such as *LRP6*, *DVL2*, *CTNNB1*, *BCL9L*, *FZD2*, and *FZD7*, were significantly enriched in the screen (Figures 1B and S1E). Supporting these results, Kyoto Encyclopedia of Genes and Genomes (KEGG) pathway enrichment showed that Wnt signaling was among the top five enriched pathways (Figure S1F). The inhibition of GSK3α/β using CHIR99021 initiates Wnt signaling downstream of FZD receptors, yet two (FZD2 and FZD7) of the ten human FZD receptors were identified as positive regulators of Wnt signaling during hESCs differentiation toward PS indicating a putative contribution of Wnt proteins-mediated signaling under these conditions (Figure 1B). To validate the screen hits, we knocked out *FZD2*, *FZD7*, and *CTNNB1* using independent sgRNAs and observed a reduction in Citrine levels three days post-CHIR99021 treatment (Figure S1G).

Next, we investigated the cell surface expression of different FZDs using antibodies that were designed to specifically bind to each of the 10 FZD receptors (Pavlovic et al., 2018; Steinhart et al., 2017). We found that FZD2 and FZD7 were highly expressed at the cell membrane of hPSCs in both H1 and WTC11 lines (Figures 1C, 1D, and S1H). Overall, these results suggest that the two main FZD receptors expressed in hPSCs, FZD2 and FZD7, have non-redundant functions during Wnt activation in hPSCs and PS formation.

Antibody agonists specifically bind and activate FZD2 and FZD7 receptors in hESCs

We recently introduced a novel antibody modality platform enabling the selective activation of one or more FZD and LRP5/6 receptors (Chidiac et al., 2021; Tao et al., 2019). These FLAgs fully mimic the function of Wnt proteins both *in vitro* and *in vivo* (Chidiac et al., 2021; Hu et al.,

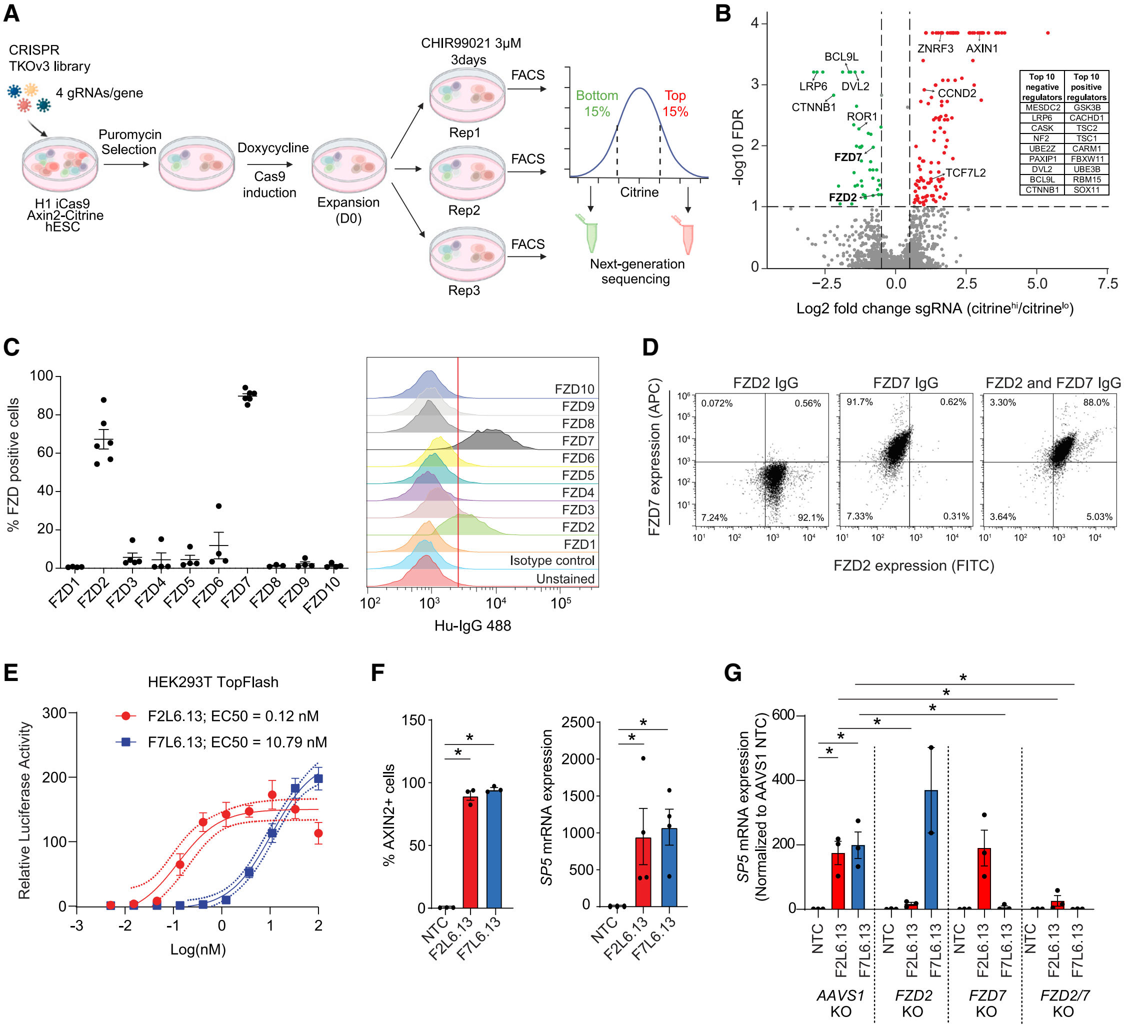

Figure 1. FZD2 and FZD7 are required for Wnt activation and primitive streak induction in hESCs

(A) Schematic representing a genome-wide CRISPR-Cas9 in H1 *AXIN2*-citrine reporter cell line treated with CHIR99021 for three days.

(B) Volcano plot of gene enrichment in the top versus bottom 15% fractions from the CRISPR screen (cutoff; log2 fold change above +/− 0.5, −log10 FDR <0.1). Green and red dots correspond to significantly enriched negative and positive regulators, respectively. Table lists the top 10 hits. Highlighted dots represent selected Wnt-related hits.

(C) FZDs expression profiling in H1 hESCs by flow cytometry using specific FZDs IgG antibodies (n = 4–6 independent experiments).

(D) Co-expression of FZD2 and FZD7 in H1 hESCs. Representative plots of three independent experiments.

(E) Dose-response curve for the activation of LEF/TCF reporter gene in HEK293T cells treated with F2L6.13 or F7L6.13 (n = 2 independent experiments).

(F) (Left) Flow cytometry analysis of the percentage of AXIN2-positive cells after one day of treatment with 30 nM of F2L6.13 or F7L6.13 using the H1 *AXIN2*-citrine reporter line (n = 3 independent experiments). (Right) RT-qPCR of *SP5* mRNA expression in H1 hESC treated with 30 nM of F2L6.13 and F7L6.13 for 24 h (n = 4 independent experiments).

(G) RT-qPCR of *SP5* mRNA expression in H1 hESC treated with 30 nM of F2L6.13 and F7L6.13 for 24 h in FZD2 and FZD7 KO hESC lines (n = 3 independent experiments).

Data are represented as mean ± SEM. Statistical analysis was performed using a two-tailed t test or one-way ANOVA followed by Tukey's *post hoc* test. $^*p \leq 0.05$ was considered significant.

2022; Nabhan et al., 2023; Tao et al., 2019; Yang et al., 2024). Leveraging this platform, we first compared FLAgs targeting the FZD2:LRP6 (F2L6.13) and FZD7:LRP6 (F7L6.13) receptor complexes (Tao et al., 2019) for their ability to activate β-catenin signaling. Treatment of HEK293T cells expressing the β-catenin-activated reporter (pBAR) (Biechele and Moon, 2008), which monitors lymphoid enhancer factor/T cell factor (LEF/TCF)-mediated transcription, showed that F2L6.13

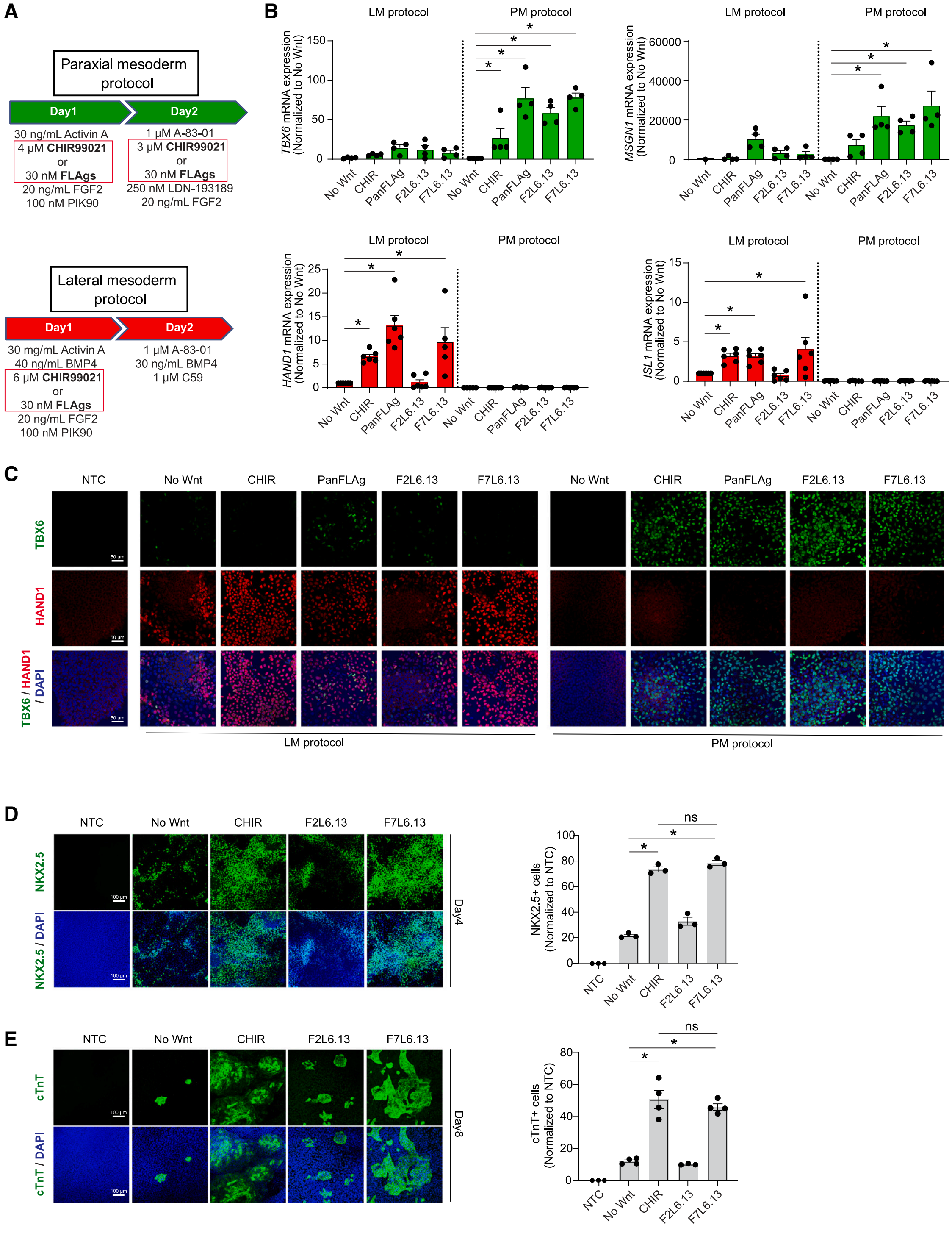

Figure 2. FZD2 and FZD7 activation promote distinct bifurcation choices into paraxial or lateral mesoderm

(A) Paraxial versus lateral mesoderm differentiation protocol adapted from Loh et al., 2016. In each differentiation protocol, CHIR99021 treatment was replaced by 30 nM of isotype control, PanFLAg, F2L6.13, or F7L6.13 treatment.

(B) RT-qPCR of hESC-derived paraxial mesoderm markers (*TBX6* and *MSGN1*) or lateral mesoderm markers (*HAND1* and *ISL1*) at day 2 of differentiation (n = 4–5 independent differentiation experiments).

(C) TBX6 (green) and HAND1 (red) immunostaining staining in paraxial versus lateral mesoderm differentiation protocol in H1 hESCs. Images are representative of three independent differentiation experiments.

(legend continued on next page)

(half maximal effective concentration (EC50) = 0.12 nM) is more potent than F7L6.13 (EC50 = 10.79 nM) (Figure 1E). To study the role of FZD2 and FZD7 in hPSC differentiation, we used 30 nM of F2L6.13 and F7L6.13 since near-maximal levels of Wnt-β-catenin signaling were observed at this dose (Figure 1E). As expected, treatment of H1 hESCs with 30 nM of F2L6.13 and F7L6.13 for 24 h increased *SP5* mRNA levels and AXIN2-Citrine expression to comparable levels (Figure 1F). To confirm the specificity of the agonists, we used CRISPR-Cas9 gene editing to generate clonal FZD2 and/or FZD7 KOs in H1 cells and confirmed the KO efficiency by sequencing (data not shown) and flow cytometry (Figure S1I). These KO clones remain pluripotent as shown by the retention of OCT4 expression (Figure S1J). Confirming the specificity of the antibody agonists, F2L6.13- or F7L6.13-mediated activation of β-catenin signaling was blunted in FZD2 and FZD7 KO lines, respectively (Figure 1G). Deletion of each individual FZD did not affect the expression or the activity of the other receptor (Figures 1G and S1I). We conclude that F2L6.13 and F7L6.13 specifically and efficiently activate β-catenin signaling in hPSCs through the respective engagement of FZD2:LRP6 and FZD7:LRP6 receptor complexes.

Activation of FZD7 but not FZD2 receptor induces lateral mesoderm

Previously published differentiation protocols used Activin-A, BMP4, FGF2, and a GSK3i such as CHIR99021 to induce PS formation and subsequent specification of cells into different mesodermal subtypes (Loh et al., 2016). To study the role of FZD2 and FZD7 during mesoderm differentiation, we replaced CHIR99021 with (1) a FLAg that binds to FZD1, 2, 4, 5, 7, and 8 (PanFLAg); (2) F2L6.13; or (3) F7L6.13 during differentiation (Figure 2A). Similar to CHIR99021 treatment, the addition of each FLAg induced PS formation as shown by increased BRACHYURY expression and reduction of OCT4 and SOX2 after one day of differentiation (Figures S2A–S2C). We next monitored mesoderm specification under conditions where CHIR99021 was substituted by F2L6.13 or F7L6.13 treatments using a panel of paraxial and lateral mesoderm markers. Interestingly, when normalized to the absence of Wnt activation (No Wnt condition), F2L6.13 significantly increased paraxial mesoderm markers *TBX6* and *MSGN1* but not lateral/cardiac mesoderm markers *HAND1* and *ISL1* (Figures 2B, 2C, and S2D). On the other hand, PanFLAg and F7L6.13 treatment increased both paraxial and lateral mesoderm markers (Figures 2B, 2C, and S2D). This result suggests that, although early activation of FZD2 and FZD7 receptors can induce Wnt/β-catenin signaling and PS formation, subsequent activation of these two FZD receptor complexes leads to distinct mesodermal cell fates. Since only treatment with F7L6.13 promoted lateral mesoderm specification, we predicted that F2L6.13 would be unable to support cardiac mesoderm differentiation and cardiomyocyte production. As expected, F7L6.13—but not F2L6.13—treatment increased cardiac mesoderm and cardiomyocyte formation above the “No Wnt” control condition, as shown by an increase in NKX2.5 and cardiac troponin (cTnT) expression, respectively (Figures 2D and 2E). We conclude that, while stimulation of FZD7 leads to both paraxial and lateral mesoderm fate specification, FZD2 activation preferentially promotes paraxial mesoderm differentiation.

To probe mechanisms and eliminate confounding effects, we assessed whether β-catenin activation using FLAg, Wnt proteins, or CHIR99021 treatments was sufficient to induce mesoderm specification independently of exogenous FGF2, ACTIVIN, and BMP4 growth factors. To do this, we treated H1 hESC with CHIR99021 (3 μM or 6 μM), rWnt3a purified protein (300 ng/mL), PanFLAg, F2L6.13, or F7L6.13 (30 nM) alone, in the absence of any exogenous factors used in standard mesoderm differentiation protocols (Figure 3A). After 4 days of PanFLAg, F2L6.13, or F7L6.13 treatment alone, we observed an increase in BRACHYURY expression to levels comparable to or higher than those after treatment with 6 μM CHIR99021 that was accompanied by a significant decrease of self-renewal markers OCT4 and SOX2 (Figures 3B and S3A–S3E). FZD4 and FZD5 receptors are not expressed in hPSCs, and therefore F4L6.13 and F5L6.13 (antibodies previously shown to activate β-catenin signaling through FZD4 and FZD5 receptors, respectively [Chidiac et al., 2021; Yang et al., 2024]) were used as negative controls. We found that stimulation of hPSCs with F2L6.13 or F7L6.13 was sufficient to induce PS (Figures 3B and 3C). We then asked if F2L6.13 or F7L6.13 treatment can derive specific mesoderm subtypes. Treating with 3 μM of CHIR99021 or 300 ng/mL of purified Wnt3a protein alone increased BRACHYURY expression slightly but did not

(D) NKX2.5 immunostaining staining (left) and flow cytometry quantification of NKX2.5 expression (right) of hESC-derived cardiac mesoderm (day 4) (*n* = 3 independent differentiation experiments). Images are representative of three independent differentiation experiments.
(E) cTnT immunostaining staining (left) and image quantification (right) of hESC-derived cardiomyocytes (day 8) (*n* = 3 independent differentiation experiments). Images are representative of three independent differentiation experiments.
Data are represented as mean ± SEM. Statistical analysis was performed using a one-way ANOVA followed by Tukey’s *post hoc* test. *$p \leq 0.05$ was considered significant.

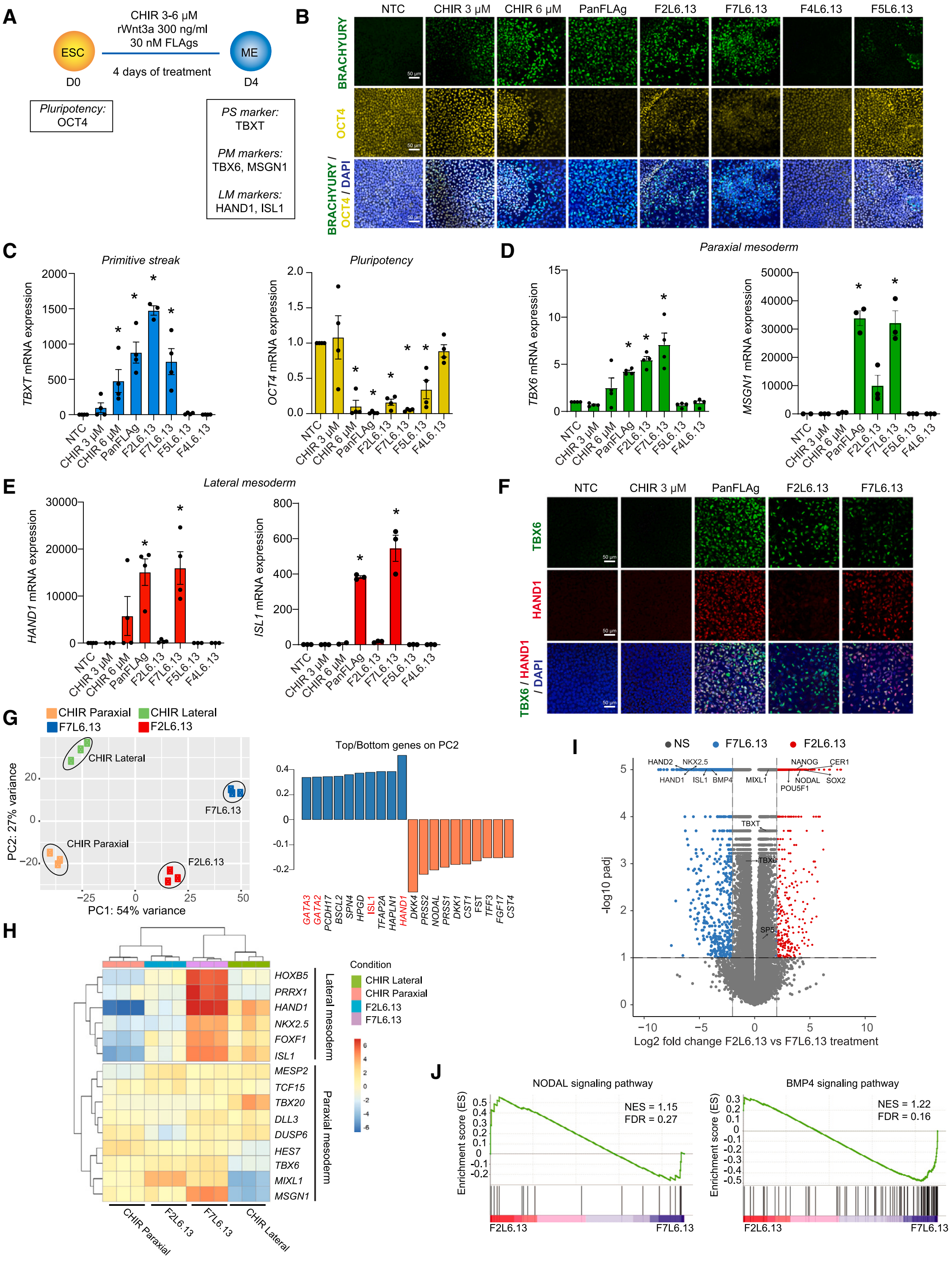

Figure 3. F2L6.13 and F7L6.13 differentially alter the transcriptome of hESCs

(A) Schematic for mesoderm (ME) differentiation following treatment of hESCs with FLAgs or CHIR99021 without any other external signaling cues.

(legend continued on next page)

induce any mesoderm specification as shown by the absence of induction of paraxial (*TBX6* and *MSGN1*) and lateral (*HAND1* and *ISL1*) mesoderm markers (Figures 3A–3F and S3A). This suggests that lower levels of β-catenin signaling can lead to PS but are not sufficient to generate mesoderm subtypes. Indeed, when cells were treated with higher doses (6 μM) of CHIR99021, an increase in paraxial and lateral mesoderm markers was observed. Notably, we found that treating hPSCs with 30 nM of F2L6.13 for 4 days significantly increased paraxial mesoderm markers expression (*TBX6* and *MSGN1*) but did not induce lateral mesoderm markers expression (*HAND1* and *ISL1*) (Figures 3D–3F). In contrast, PanFLAg and F7L6.13 treatment significantly increased the expression of both lateral and paraxial mesoderm markers (Figures 3D–3F). PS is known to form definitive endoderm and mesoderm. In the absence of other signaling cues used in directed differentiation protocols, F2L6.13 and F7L6.13 treatment also increased SOX17 (endoderm marker) indicating the formation of a heterogeneous mesendoderm population (Figures S3C and S3E). Overall, these results suggest that FZD2 activation drives hPSCs differentiation only toward paraxial mesoderm, whereas FZD7 activation promotes the formation of paraxial and lateral mesoderm.

Bifurcation of paraxial versus lateral mesoderm using F2L6.13 and F7L6.13 treatment

To understand the mesodermal lineage differentiation bias between the two selective FLAgs targeting FZD2 and FZD7 receptors, we performed bulk RNA sequencing in H1 hESCs treated with either F2L6.13 or F7L6.13 for 4 days. We compared these treatments to the standard protocols used to derive paraxial or lateral mesoderm. Principal-component analysis (PCA) showed clustering of the F7L6.13-treated population with the lateral mesoderm protocol (CHIR_Lateral) and the F2L6.13-treated population with the paraxial mesoderm protocol (CHIR_Paraxial) on the principal component 2 (PC2) axis (Figure 3G). Genes involved in lateral mesoderm formation (e.g., *GATA2*, *GATA3*, *ISL1*, and *HAND1*) were among the top 10 genes contributing to the variance explained by PC2 (Figure 3G), further confirming that PC2 represents the paraxial-lateral mesoderm axis of variation. Using a curated list of paraxial and lateral mesoderm markers, we found that F7L6.13 treatment induced both mesoderm progenitor lineages whereas F2L6.13 preferentially promoted paraxial mesoderm (Figure 3H). We next performed differential gene expression analysis comparing F2L6.13 and F7L6.13 treatments (Figure 3I and Table S2). Paraxial mesoderm genes were not differentially regulated reflecting that both treatments increase paraxial mesoderm markers. However, lateral/cardiac mesoderm genes (e.g., *HAND1*, *ISL1*, and *NKX2.5*) were significantly enriched in the F7L6.13 treatment. Interestingly, *BMP4* expression was significantly increased with F7L6.13 treatment and *NODAL*, *LEFTY1*, and *CER1* were significantly upregulated with F2L6.13 treatment. These genes are known to play different/opposing roles in cell fate specification during PS formation (Kattman et al., 2011; Nostro et al., 2008; Sumi et al., 2008; Xu et al., 2014). For instance, BMP4 plays an important role in posteriorizing and lateral mesoderm formation and NODAL is increased during anterior cell specification (Martyn et al., 2018; Tsaytler et al., 2023). Accordingly, gene set enrichment analysis (GSEA) comparing the transcriptome of the two treatments revealed that BMP4 signaling was enriched in the F7L6.13-treated cells whereas NODAL signaling was enriched in the F2L7.13-treated cells (Figure 3J). Using qPCR, we validated that *BMP4* mRNA levels were highly upregulated following F7L6.13—but not F2L6.13—treatment. BMP4 signaling inhibition using LDN193189 significantly reduced the potency of F7L6.13 in deriving lateral mesoderm (Figure S3F). These results

(B and C) Immunostaining (B) and RT-qPCR (C) of the primitive streak (*TBXT*) and pluripotency (*OCT4*) markers after 4 days of treatment with the indicated doses of CHIR99021 or 30 nM of different FLAgs ($n = 4$ independent differentiation experiments). Images in (B) are representative of three independent differentiation experiments.
(D and E) RT-qPCR of H1 hESC-derived paraxial mesoderm (*TBX6* and *MSGN1*) (D) and lateral mesoderm (*HAND1* and *ISL1*) (E) markers after 4 days of treatment with the indicated doses of CHIR99021 or 30 nM of different FLAgs ($n = 3$–4 independent differentiation experiments).
(F) Immunostaining of TBX6 and HAND1 in CHIR99021-, PanFLAg-, F2L6.13-, or F7L6.13-treated H1 hESCs. Images are representative of three independent differentiation experiments.
(G) (Left) Principal-component analysis of RNA sequencing comparing F2L6.13 and F7L6.13 treatment to the standard differentiation protocol in which CHIR99021 treatment was used to drive specifically paraxial or lateral mesoderm. (Right) Top 10 genes defining PC1 and PC2 were listed. Lateral mesoderm markers were highlighted in red. For RNA sequencing, three independent differentiation experiments per condition were performed.
(H) Heatmap of hierarchical clustering of paraxial and lateral mesoderm makers.
(I) Volcano plot of gene enrichment in F2L6.13- versus F7L6.13-treated H1 hESCs (30 nM, 4 days). F2L6.13-enriched genes (red circles) and F7L6.13-enriched genes (blue circles) were highlighted.
(J) GSEA enrichment analysis shows NODAL and BMP4 gene sets enriched in F2L6.13 and F7L6.13 treatment, respectively.
Data are represented as mean ± SEM. Statistical analysis was performed using a one-way ANOVA followed by Tukey's *post hoc* test. $^{*}p \leq 0.05$ was considered significant.

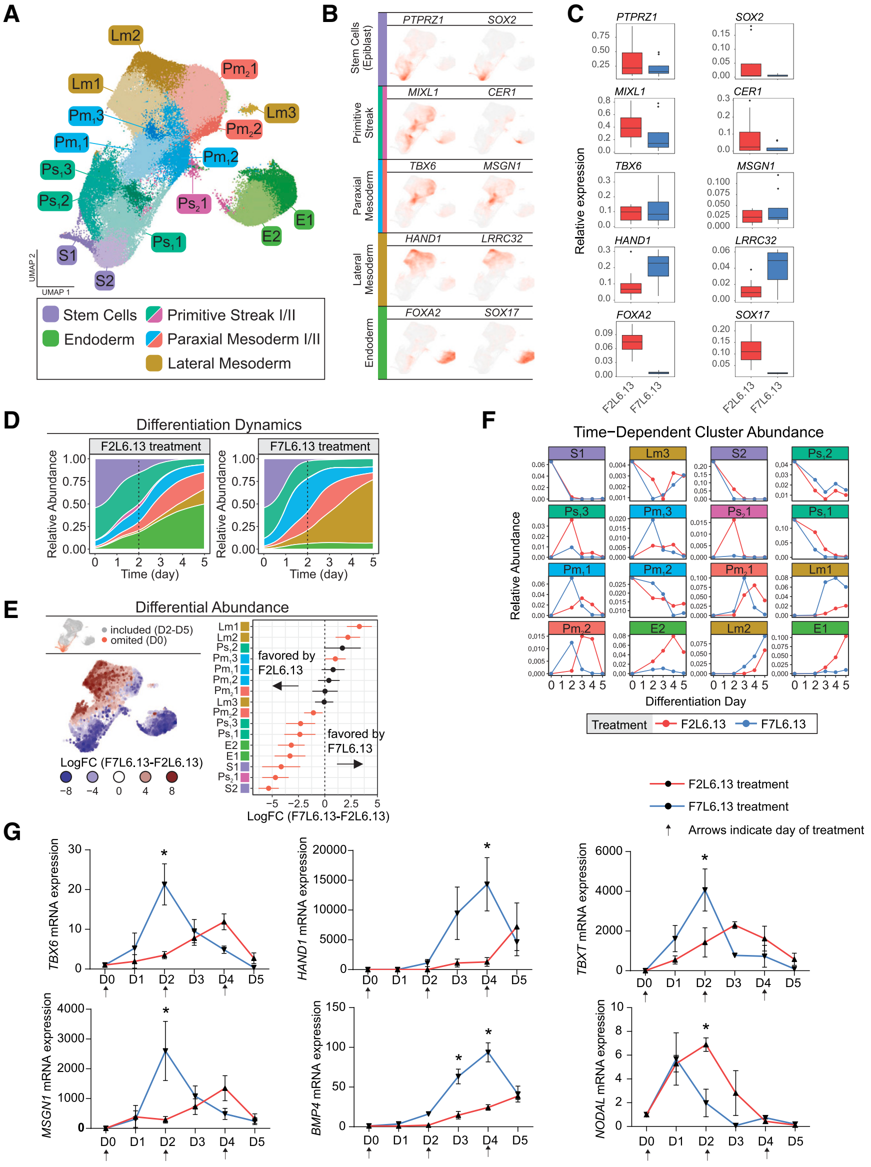

Figure 4. Single-nucleus transcriptome map of F2L6.13- and F7L6.13-induced mesoderm differentiation from hESCs

(A) Uniform manifold approximation and projection (UMAP) of integrated single-cell populations profiled over differentiation time course.

(B) UMAPs showing expression of cell-type-specific markers.

(C) Boxplots of cell-type-specific markers in F2L6.13- vs. F7L6.13-treated hESCs. Data represent sample-level average expression for each day and treatment condition. D0 samples were omitted from this analysis.

(D) Stream plot of relative abundances of each cell type as a function of time treated with F2L6.13 (left) or F7L6.13 (right). The same colors are used to indicate cell types as in (A).

(E) Differential abundance analysis between F2L6.13- and F7L6.13-treated cells represented as UMAP (*left*) and forest plot (*right*). UMAP nodes represent cellular neighborhoods. Differentially abundant populations are indicated in red on forest plots (5% FDR, Wilcoxon test).

(F) Relative abundance of cell subpopulations across five differentiation days (n = 2 independent differentiation experiments/time point/treatment).

(G) Time course RT-qPCR analysis of stage-specific markers including primitive streak (*TBXT*), paraxial mesoderm (*TBX6* and *MSGN1*), lateral mesoderm (*HAND1* and *BMP4*), and endoderm (*NODAL*) in H1 hESCs treated with 30 nM of F2L6.13 or F7L6.13 for 5 days. Arrows indicate the day of treatment (n = 4 independent differentiation experiments). Data are represented as mean ± SEM. Statistical analysis was performed using a one-way ANOVA followed by Tukey's *post hoc* test. $*p \leq 0.05$ was considered significant.

suggest that F7L6.13 mediates lateral mesoderm differentiation in part through the activation of BMP4 signaling.

snRNA-seq reveals temporal differentiation dynamics in response to F2L6.13 and F7L6.13 treatment

We next used single-nucleus RNA sequencing (snRNA-seq; sci-RNA-seq3 protocol) to profile the temporal transcriptomic changes and differentiation kinetics of F2L6.13- and F7L6.13-treated H1 hESCs during days 2–5 of mesoderm induction. After stringent quality control, filtering and batch-effect correction, 81,294 nuclei were included for downstream analysis with a median of 2,097 genes detected per nucleus. We identified 16 distinct cell clusters, representing subpopulations of stem cells (S1-2), PS ($Ps_1$1-3 and $Ps_2$1), paraxial ($Pm_1$1-3 and $Pm_2$1-2) and lateral (Lm1-3) mesoderm, and endoderm (E1-2) (Figures 4A and S4A). Cell-type annotations were based on cell-type marker profiles (Figures 4B and S4B) and reference atlas-based transfer learning (see supplemental experimental procedures). Each temporal dynamic class was dissected to ensure cell-type compositions were well resolved. In line with our previous data, F7L6.13 but not F2L6.13 treatment induced increased lateral mesoderm markers *HAND1*, while F2L6.13 treatment increased endoderm markers *SOX17* and *FOXA2*

(Figure 4C). We also performed differential expression analyses based on the time-dependent relative abundance similarities of each cluster and their lineage relationships (Figures 4D–4F). As expected, the abundance of stem cell states decreased over time as shown by pluripotency markers such as *SOX2* and *PTPRZ1*, whereas mesodermal and endodermal progenitors expressing specific markers such as *TBX6*, *MSGN1*, *HAND1*, and *SOX17* emerged as early as day 2 (Figure 4D). Next, we compared the relative abundance between F2L6.13- and F7L6.13-treated cells during differentiation to understand the identity of differentially regulated cell populations between the two treatments. Lateral mesoderm subpopulations (Lm1 and Lm2; defined by *HAND1*, *ISL1*, and *LRRC32* expression) were significantly enriched in F7L6.13- but not F2L6.13-treated hPSCs. We also found that the paraxial mesoderm subpopulations ($Pm_1$1-3, defined by *TBX6*, *MSGN1*, *DLL1*, and *DLL3*) were equally represented in F2L6.13- and F7L6.13-treated conditions (Figures 4E, 4F, and S4C). Endoderm subpopulations (E1 and E2; defined by *SOX17* and *FOXA2*) were significantly enriched by F2L6.13 treatment. Interestingly, the endoderm lineage was preceded by a rare and distinct DKK4+/CER1+ PS subpopulation $PS_2$1 that was only detected in F2L6.13- but not F7L6.13-treated cells (Figures 4E, S4B, and S4C). This is in agreement with the finding that F2L6.13 treatment significantly increased *NODAL* expression after 2 days of treatment when compared to F7L6.13 treatment (Figure 4G). In addition, pluripotent cell state markers and early PS markers were also significantly favored by F2L6.13 treatment indicating that cells treated with F2L6.13 are slower to exit the pluripotency state when compared to F7L6.13 treatment (Figures 4E, 4F, and S4C). The kinetics difference in the lateral and paraxial mesoderm formation was further validated by qPCR analysis using specific markers (Figure 4G).

Taken together, our bulk and snRNA-seq expression analyses support the conclusion that β-catenin signaling activation through selective FZD7 or FZD2 activation leads cells to adopt a paraxial mesoderm fate and that FZD7 activation additionally promotes lateral mesoderm specification.

Kinetics of Wnt signaling activation governs mesodermal fate induction

A Wnt signaling gradient is known to modulate cell fate specification during gastrulation, but the contribution of different FZD receptors remains largely unexplored. Our snRNA-seq findings led us to hypothesize that the distinct kinetics of Wnt-β-catenin signaling downstream of FZD2 and FZD7 promote unique mesoderm fate specification. To test this, we examined the activation kinetics of the Wnt target gene *SP5* in response to F2L6.13 and F7L6.13 treatment (Figure 5A). Interestingly, we found that both FZD agonists caused similar steady increases in *SP5* expression during the early phase of treatment (days 0–2). However, after day 2, the activation kinetics of the Wnt-β-catenin pathway began to diverge (Figure 5A). Whereas F2L6.13 treatment resulted in sustained *SP5* expression for 4–5 days, a rapid attenuation of *SP5* expression was observed following 2 days of F7L6.13 treatment (Figure 5A). CHIR99021 treatment at a high concentration (12 μM) mirrored the kinetics of F7L6.13-mediated Wnt activation (Figure S4D).

To evaluate temporal Wnt pathway activity at a single-cell level, we generated a clonal WTC11 SP5-mCherry reporter cell line (Figure 5B). Flow cytometry analysis indicated that all cells were SP5-mCherry+ after just one day of treatment with CHIR99021 (12 μM), F2L6.13, or F7L6.13 (30 nM), and this was sustained until at least day 4 (Figure 5C). To directly monitor the activation kinetics in real time, we performed live-cell imaging experiments and measured SP5-mCherry intensity in individual cells. Consistent with our mRNA results, F2L6.13 treatment was associated with sustained SP5 upregulation compared to F7L6.13 treatment (Figure 5D). Similar results were observed in the H1 AXIN2-Citrine reporter line (Figure S4E).

Next, we investigated whether lower concentrations of F2L6.13 would enhance lateral mesoderm formation by treating cells with a maximal dose of 30 nM F2L6.13 or F7L6.13 for 2 days followed by a dose-response treatment starting on day 2. Decreasing concentration of F2L6.13 showed lower Wnt activation and increased lateral formation on day 4 (Figure S5A). Moreover, we used the Wnt-β-catenin inhibitor XAV939 to block F2L6.13-induced Wnt signaling after day 2 of differentiation. We found that lateral mesoderm formation was significantly enhanced in F2L6.13-treated cells reaching levels similar to F7L6.13-treated cells (Figure S5B). No difference in paraxial mesoderm formation was observed. These results support our findings that the differences in F2L6.13- and F7L6.13-mediated differentiation are rooted in their different ability to activate Wnt-β-catenin signaling over time, which is known to influence cell fate determination. Interestingly, when mining our transcriptomic data, we observed that FZD7 expression decreased in F7L6.13-treated cells while FZD2 expression increased in F2L6.13-treated cells, possibly offering a mechanism underlying sustained β-catenin engagement by the FZD2 agonist antibody (Figure S5C). The complete mechanisms regulating FZDs expression and activation during differentiation are therefore complex and will require further studies.

Finally, we sought to confirm that the observed differences in signaling kinetics and cell specification outcomes following F2L6.13 and F7L6.13 treatments were a property of the activated receptor and signaling network and not due to different properties of the antibodies. To do this,

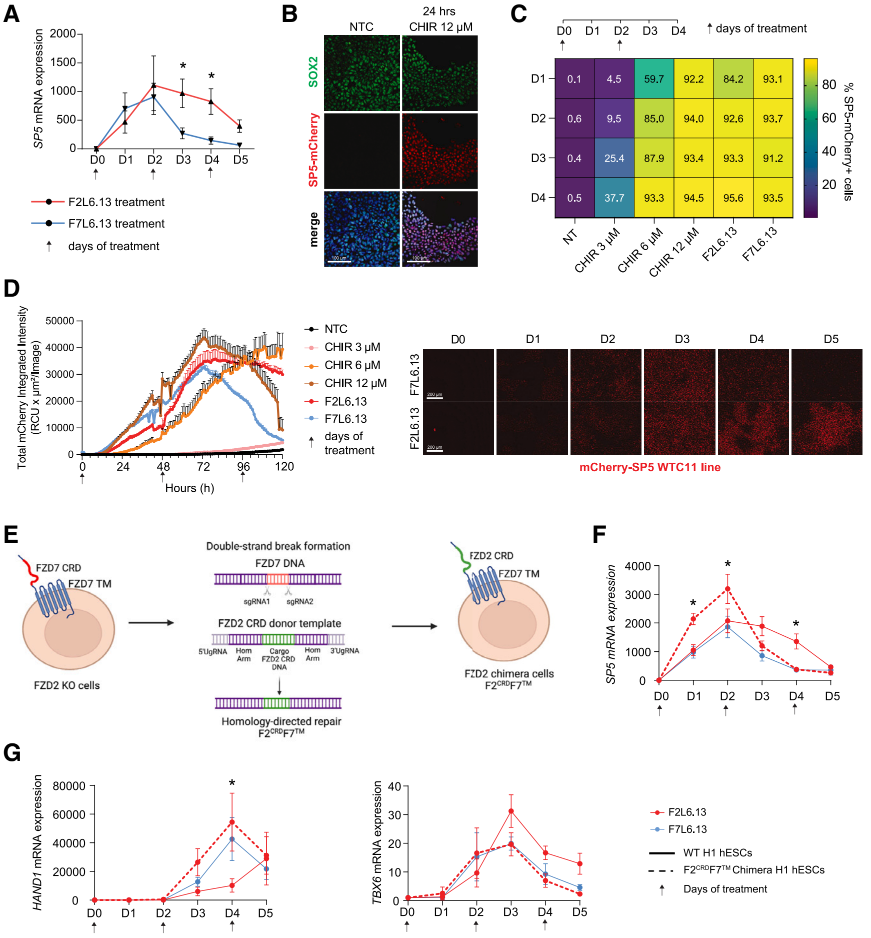

Figure 5. Wnt activation kinetics dictates the fate of mesoderm differentiation

(A) Time course RT-qPCR analysis of *SP5* mRNA expression in H1 hESCs treated with 30 nM F2L6.13 and F7L6.13 for 5 days. Arrows indicate the day of treatment (n = 4 independent differentiation experiments).

(B) Immunostaining against SOX2 and mCherry expression in the SP5-mCherry H1 hESC reporter line following 24 h treatment with 12 μM CHIR99021. Images are representative of two independent experiments.

(C) Heatmap of flow cytometry quantification of the percentage of SP5-mCherry-positive cells in WTC SP5-mCherry reporter line treated for 5 days with different doses of CHIR99021 or 30 nM of F2L6.13 or F7L6.13. Cells were harvested for flow cytometry every day (n = 3 independent experiments).

(D) (Left) Quantification of time-lapse imaging of SP5-mCherry fluorescence intensity over 5 days of differentiation. (Right) Representative images of SP5-mCherry cells treated with either F2L6.13 or F7L6.13 for 5 days (mean ± SEM of 9 different images per treatment). Experiment was repeated twice with similar results.

(E) Diagram showing the CRISPR-Cas9 gene editing engineering of hPSC H1 chimera cell line. Using specific sgRNA, the FZD7 CRD is cut out and replaced by the FZD2 CRD domain on a FZD7 transmembrane domain creating FZD2 chimera cells ($F2^{CRD}F7^{TM}$).

(F and G) Time course RT-qPCR analysis of SP5 (F) and HAND1 or TBX6 (G) mRNA expression in H1 hESCs treated with F2L6.13 and F7L6.13 or $F2^{CRD}F7^{TM}$ chimera cells treated with F2L6.13 for 5 days (n = 3 independent differentiation experiments). Data are represented as mean ± SEM. Statistical analysis was performed using a one-way ANOVA followed by Tukey's *post hoc* test. *$p \leq 0.05$ was considered significant.

we generated a chimeric receptor $F2^{CRD}F7^{TM}$ in which the N-terminal cysteine-rich domain (CRD) of FZD7 is replaced by the FZD2 CRD while preserving the transmembrane domain (TM) of FZD7. We used our clonal FZD2 KO H1 lines and precisely swapped the FZD7 CRD within the FZD7 open reading frame with the coding sequence of the FZD2 CRD (Figures 5E and S5D). $F2^{CRD}F7^{TM}$ chimera line was confirmed by sequencing and flow cytometry (Figures S5D and S5E). Strikingly, $F2^{CRD}F7^{TM}$ cells treated with F2L6.13 did not show sustained Wnt-β-catenin activation observed in the H1 parental cells. In contrast, F2L6.13 treatment in the $F2^{CRD}F7^{TM}$ cells led to an early stimulation of the Wnt target genes *SP5* that was rapidly dampened, mimicking F7L6.13 stimulation in the parental cells (Figure 5F). In agreement with the observed Wnt-β-catenin activation kinetics, stimulation of hESCs expressing the $F2^{CRD}F7^{TM}$ chimeric receptor with F2L6.13 led to robust expression of *HAND1* (lateral mesoderm) and to faster dampening of *TBX6* (paraxial mesoderm), both mimicking the response obtained following FZD7 stimulation (Figure 5G).

Collectively, our data show that sustained Wnt pathway activation downstream of the FZD2 receptor promotes paraxial mesoderm and represses lateral mesoderm. In contrast, transient activation of the Wnt-β-catenin pathway through the FZD7 receptor promotes the lateral mesoderm. Therefore, our results reveal that activation of different FZD-containing receptor complexes leads to distinct Wnt-β-catenin signaling kinetics that influences cell differentiation outcomes such as mesodermal cell fate bifurcation choices.

DISCUSSION

Wnt-β-catenin signaling governs mesendoderm lineage specification; however, the role of individual FZD receptors

in regulating hPSC differentiation is poorly defined. Here, using selective FZD antibody agonists, we precisely controlled the activation of Wnt signaling at the receptor level during mesoderm cell fate specification and uncovered that stimulation of FZD2 and FZD7 led to different differentiation outcomes. Mechanistically, we demonstrated that stimulation of FZD2 and FZD7 activates β-catenin signaling with different kinetics and transcriptional changes promoting distinct mesodermal lineage bifurcation choices.

Selective activation of FZD receptor complexes using antibody-based molecules that mimic Wnt proteins and efficiently stimulate the pathway has only recently been developed (Janda et al., 2017; Tao et al., 2019). In previous studies, we showed that a pan-acting agonist antibody that binds to FZD1, 2, 4, 5, 7, 8, and LRP6 can induce the differentiation of hPSCs into mesoderm (Tao et al., 2019), whereas a selective FZD5:LRP6 agonist was shown to pattern neural progenitors into midbrain fate (Yang et al., 2024). A FZD7:LRP6 tetravalent antibody agonist was also shown to induce mesendodermal differentiation of hESCs (Gumber et al., 2020). The access to highly selective Wnt surrogates therefore provides an emerging option for optimal spatiotemporal control of Wnt-β-catenin signaling to enhance directed differentiation of hPSCs.

Our transcriptomic data reveal that H1 hESCs express FZD2, FZD3, FZD5, and FZD7, consistent with findings from other studies (Fernandez et al., 2014). However, when examining protein levels using the selective FZD antibodies, we observed that FZD2 and FZD7 receptors are predominantly expressed at the membrane among the ten different FZDs. This discrepancy underscores the importance of assessing FZD receptor protein levels at the membrane, as they may differ from their transcriptional expression. FZD2 and FZD7 share strong sequence homology and were suggested to play redundant roles during development (Sagara et al., 1998; Yu et al., 2012). FZD7 is known to be the most abundant FZD receptor in undifferentiated hPSCs (Fernandez et al., 2014). Our genome-wide CRISPR screen designed to identify regulatory mechanisms of Wnt-β-catenin signaling during PS formation suggested a unique and non-redundant function for FZD2 and FZD7 receptors in hESCs. This also suggests that, during CHIR-driven hPSC differentiation, β-catenin signaling contributes to the upregulation and secretion of Wnt proteins that engage FZD2 and FZD7, consistent with the literature demonstrating a positive feedback mechanism where Wnt pathway activation enhances Wnt ligand expression (Nakamura et al., 2011).

Although both *AXIN2* and *SP5* are target genes of Wnt signaling, *SP5* is a more robust marker for pathway activation in stem cells and provides a more accurate measure than *AXIN2*, which shows a transient and weaker response following Wnt3a and GSK3β inhibitor treatments (Gumber et al., 2020; Huggins et al., 2017; Söderholm et al., 2023). We showed that, while the Wnt target gene *SP5* was equally upregulated 24 h and 48 h following F2L6.13 and F7L6.13 treatments in hPSCs, F7L6.13 induced a faster exit from the pluripotency state when compared to F2L6.13. This result suggests early differences in gene expression regulation following activation of FZD2 and FZD7. Whole and single-cell transcriptomic analysis further revealed that F2L6.13 and F7L6.13 elicit distinct transcriptional responses associated with mesoderm specification. FZD7 but not FZD2 activation showed strong induction of lateral/cardiac mesoderm markers such as *HAND1*, *ISL1*, *LRRC32*, *NKX2.5*, *BMP4*, *GATA2*, and *GATA3* (Loh et al., 2016). Our snRNA-seq data show that F2L6.13 treatment forms unique PS subtypes ($Ps_1 3$ and $Ps_2 1$) characterized by the expression of anterior PS genes such as *DKK4*, *CER1*, and *FOXA2*. This suggests that $Ps_1 3$ and $Ps_2 1$ cell populations might represent the anterior PS generated by F2L6.13, potentially acting as a transitional population toward paraxial mesoderm and endoderm populations while restricting lateral mesoderm formation.

Highlighting the engagement of regulatory loops supporting lineage commitment, F2L6.13 and F7L6.13 differentially trigger the expression of distinct growth factors such as *NODAL* and *BMP4*, respectively. FZD2 activation favors endoderm and paraxial mesoderm, whereas FZD7 activation promotes paraxial and lateral mesoderm, consistent with the role of NODAL in anterior PS patterning for endoderm and the role of BMP4 in mesoderm specification (Kim et al., 2015). In addition, the transcriptional hierarchy of BMP4 to Wnt to NODAL is conserved in hESCs and during gastrulation (Chhabra et al., 2019; Martyn et al., 2018). Practically, BMP4 has been harnessed to form posterior PS-like cells and induce lateral plate mesoderm patterning and cardiac mesoderm differentiation (Rojas et al., 2005; Tsaytler et al., 2023) while Activin/NODAL pathway maintains pluripotency and drives definitive endoderm specification (Brown et al., 2011; Osnato et al., 2021).

Wnt-β-catenin signaling dynamics vary dramatically in response to Wnt ligands depending on contextual factors such as the stage of differentiation or the cell type (Massey et al., 2019). A precise Wnt gradient governs the anteroposterior patterning of PS (anterior vs. posterior PS) and mesoderm specification (paraxial vs. lateral/cardiac mesoderm) (Wu et al., 2024; Zhao et al., 2019). We showed that, although activation of FZD2 and FZD7 leads to similar levels of early β-catenin target gene activation, the kinetics of activation differs between the two receptors and

correlates with the observed differentiation outcomes. The robust and strong activation of Wnt signaling obtained using high doses of CHIR00921 treatment (higher than 6 μM) followed by the use of Wnt inhibitors such as the porcupine inhibitor (IWP2) is commonly used to generate cardiac mesoderm differentiation (Lian et al., 2013). We observed that FZD7 activation stimulates a strong early phase of Wnt-β-catenin activation that is inhibited on its own within 48 h similar to what is observed following treatment with 12 μM of CHIR99021. In contrast, FZD2 activation led to sustained activation kinetics compared to FZD7 activation or CHIR99021-mediated β-catenin signaling. This sustained activation of Wnt target genes is also associated with a slower emergence of paraxial mesoderm while lateral mesoderm is blocked. It has been shown that the continuous expression of *TBX6* can suppress cardiac differentiation and induce paraxial mesoderm and somite lineage specification (Sadahiro et al., 2018). *TBX6* and *MGSN1* expression slowly and continuously increase in F2L6.13-treated cells whereas F7L6.13 treatment is faster in increasing *TBX6* expression. This kinetics profile is in correlation with a slower exit of pluripotency induced by the FZD2 antibody agonist.

One important question raised by our findings is how activation of two structurally related receptors (FZD2 and FZD7) leads to distinct β-catenin activation profiles. Our results demonstrate that the kinetics of β-catenin transcriptional activity differs following activation of FZD2 and FZD7. Mechanistically how this is achieved remains to be determined but could be a result of different rates of receptor endocytosis or recycling to the plasma membrane or ligand-dependent receptor degradation leading to dampening of the response. Alternatively, these receptors could localize to different plasma membrane subdomains, interact with different effectors, or transit to separate endocytic routes influencing the extent of β-catenin stabilization. Importantly, these differences are intrinsic to the individual FZD receptors, and not a property of the ligand, since swapping the CRD of FZD7 for the CRD of FZD2 was found to change β-catenin activation kinetics in response to the FZD2 antibody agonist F2L6.13 when compared to wild-type cells.

Altogether, our work uncovered that activation of FZD2 and FZD7 in hES cells leads to distinct kinetics of ß-catenin-mediated transcriptional response that influences cell differentiation. Given the complex network of 19 Wnt and 10 FZD receptors, these differences are likely to pervasively influence lineage commitment during development and tissue homeostasis. This therefore constitutes a previously underappreciated layer of regulation in addition to the spatiotemporal control of Wnt proteins and FZD receptor expression. Whether these differences in β-catenin activation kinetics play important roles *in vivo* remains to be determined but could, as we demonstrated, be leveraged to improve directed differentiation of hPSCs into more homogenous or functional cell types.

EXPERIMENTAL PROCEDURES

For additional information, see supplemental experimental procedures.

Maintenance of hPSCs

The hPSC line H1 hESCs (male) were obtained from the WiCell Research Institute (WAe001-A). WTC11 hiPSC line (male) was obtained from the Conklin Lab at Gladstone Institutes, UCSF (UCSFi001-A). H1 hESCs and WTC11 hiPSCs were cultured on Geltrex-coated plates in a StemFlex basal medium. hPSCs were maintained and expanded using established procedures and detailed in the supplemental information.

Cell differentiation

hPSCs were differentiated into mesoderm and downstream lineages as previously described (Loh et al., 2016). Alternatively, mesoderm differentiation was also examined in response to only Wnt activation in the absence of any external cues using specific concentrations of CHIR99021 or FLAg molecules every 2 days for 5 days total.

Generation of cell lines

H1 AXIN2*-Citrine reporter cell line*

H1 iCas9 *AXIN2*-Citrine reporter hESC line was generated by replacing the first exon of *AXIN2* with a cDNA coding for histone H2B fused to the Citrine fluorescent protein into H1 hESCs that were previously engineered to express doxycycline-inducible Cas9 (see supplemental information, Table S3).

WTC11 SP5 reporter cell line

The pGTag-NLS-eGFP-SV40 vector (Addgene #117811) was adapted to apply the GeneWeld method (Wierson et al., 2020) for generating the SP5-2A-mCherry reporter allele using the H1 hESCs. The knockin vector contains a 2A-mCherry reporter cassette, a puromycin selection marker, and *LoxP* sites for marker removal. The vector also features universal sgRNA sites for Cas9-induced double-strand breaks, releasing the knockin sequence for integration. Electroporation was used to deliver the CRISPR components, and cells were selected with puromycin before isolating monoclonal lines, with the undifferentiated state confirmed by OCT4 and SOX2 staining (see supplemental information, Table S3).

$F2^{CRD}F7^{TM}$ chimera line

FZD7 CRD was replaced by FZD2 CRD using the GeneWeld CRISPR-Cas9 knockin strategy. Two sgRNAs were designed to induce double-strand breaks enabling the removal of the FZD7 CRD. A donor vector, containing an FZD2 CRD with 48 bp homology arms, was designed with silent mutations to prevent further cleavage by Cas9. H1 hESCs FZD2 KO cells were transfected with the donor vector and universal sgRNAs to facilitate the integration of FZD2 CRD on the FZD7 receptor. Cells were sorted by fluorescence-activated cell sorting (FACS), and genotyping confirmed correct integration.

The undifferentiated state was validated by OCT4 and SOX2 staining (see supplemental information, Table S3).

CRISPR-Cas9 screen

H1 iCas9 *AXIN2*-Citrine cells were infected with the TKOv3 sgRNA library (Addgene #90924) at an MOI of 0.3, with 400-fold coverage. After 48 h of puromycin selection, cells were treated with doxycycline and split into 3 biological replicates, each treated with 3 μM of CHIR99021, and then sorted by citrine expression (top and bottom 15%) using FACS. Genomic DNA was extracted, sequenced, and analyzed for sgRNA representation. Hits were ranked by false discovery rate (FDR), and KEGG pathway enrichment was performed on top and bottom 15% gene hits (see supplemental information, Table S1).

Flow cytometry

Cells were harvested and stained with viability dyes or antibodies (Table S3) and then fixed before being run on the CytoFLEX S flow cytometer. Flow cytometry data were analyzed using the FlowJo software (see supplemental information).

Bulk RNA sequencing and snRNA-seq analysis

See supplemental information for processing and analysis methods.

Immunofluorescence

H1 hESCs treated with FLAgs and CHIR99021 were fixed, permeabilized, and blocked before incubation with primary antibodies (Table S3) and Alexa Fluor-labeled secondary antibodies. Cells were then imaged on a Zeiss LSM700 confocal microscope, and images were processed using ImageJ and Photoshop (see supplemental experimental procedures).

Reverse-transcription PCR

Detailed methods, including primer sequences, can be found in the supplemental information as well as Table S3.

Statistics

Statistical analyses were performed with the Prism 8 software (GraphPad, San Diego, CA, USA). The number of independent experiments is indicated in the figure legends. Graphs represent the mean ± SEM. Statistical significance was determined using a two-tailed Student's t test to compare the means of two groups or a one-way ANOVA followed by *post hoc* Tukey's multiple comparisons test to compare different groups with one variable. $^{*}p \leq 0.05$ was considered significant.

RESOURCE AVAILABILITY

Lead contact

Further information and requests for resources and reagents should be directed to and will be fulfilled by the lead author, Stephane Angers (stephane.angers@utoronto.ca).

Materials availability

Cell lines generated in this study are available from the lead contact upon request.

Data and code availability

The accession number for the RNA-seq data reported in this paper is deposited at the Gene Expression Omnibus (GEO) Database: GSE267334 and the snRNA-seq data is deposited to Figshare: https://doi.org/10.6084/m9.figshare.27228831.v1.

ACKNOWLEDGMENTS

The Center for Pharmaceutical Oncology provided the support and instruments that were used in these experiments. We also thank the Temerty Faculty of Medicine flow cytometry facility for assisting us with cell sorting experiments. We would also like to thank all members of the Angers labs for helpful discussion throughout this study. This work was supported by the University of Toronto Medicine by Design program (MBDC2-2019-03 to J.M. and S.A.), which receives funding from the Canada First Research Excellence Fund. Figure 5E and the graphical abstract were created with Biorender.com.

AUTHOR CONTRIBUTIONS

Conceptualization, R.C. and S.A.; methodology, R.C., A.Y., E.K., N.M., H.H., M.P.A., P.E.T., S.L., J.M., and S.A.; formal analysis, R.C., A.Y., E.K., N.M., H.H., and S.A.; investigation, R.C., A.Y., E.K., N.M., H.H., M.P.A., and G.M.; resources, J.-P.G., J.M., and S.A.; writing – original draft, R.C.; writing – review and editing, R.C., A.Y., N.M., P.E.T., G.M., and S.A.; funding acquisition and supervision, J.M. and S.A.

DECLARATION OF INTERESTS

S.A. is an inventor on patents for the antibodies described in the manuscript.

SUPPLEMENTAL INFORMATION

Supplemental information can be found online at https://doi.org/10.1016/j.stemcr.2024.102391.

Received: June 6, 2024
Revised: December 11, 2024
Accepted: December 12, 2024
Published: January 16, 2025

REFERENCES

Ang, L.T., Nguyen, A.T., Liu, K.J., Chen, A., Xiong, X., Curtis, M., Martin, R.M., Raftry, B.C., Ng, C.Y., Vogel, U., et al. (2022). Generating human artery and vein cells from pluripotent stem cells highlights the arterial tropism of Nipah and Hendra viruses. Cell *185*, 2523–2541.e30. https://doi.org/10.1016/j.cell.2022.05.024.

Biechele, T.L., and Moon, R.T. (2008). Assaying beta-catenin/TCF transcription with beta-catenin/TCF transcription-based reporter constructs. Methods Mol. Biol. *468*, 99–110. https://doi.org/10.1007/978-1-59745-249-6_8.

Brown, S., Teo, A., Pauklin, S., Hannan, N., Cho, C.H.-H., Lim, B., Vardy, L., Dunn, N.R., Trotter, M., Pedersen, R., and Vallier, L. (2011). Activin/Nodal signaling controls divergent transcriptional networks in human embryonic stem cells and in endoderm

progenitors. Stem Cell. *29*, 1176–1185. https://doi.org/10.1002/stem.666.

Chalamalasetty, R.B., Dunty, W.C., Jr., Biris, K.K., Ajima, R., Iacovino, M., Beisaw, A., Feigenbaum, L., Chapman, D.L., Yoon, J.K., Kyba, M., and Yamaguchi, T.P. (2011). The Wnt3a/β-catenin target gene Mesogenin1 controls the segmentation clock by activating a Notch signalling program. Nat. Commun. *2*, 390. https://doi.org/10.1038/ncomms1381.

Chen, H., Lu, C., Ouyang, B., Zhang, H., Huang, Z., Bhatia, D., Lee, S.-J., Shah, D., Sura, A., Yeh, W.-C., and Li, Y. (2020). Development of potent, selective surrogate WNT molecules and their application in defining frizzled requirements. Cell Chem. Biol. *27*, 598–609.e4. https://doi.org/10.1016/j.chembiol.2020.02.009.

Chhabra, S., Liu, L., Goh, R., Kong, X., and Warmflash, A. (2019). Dissecting the dynamics of signaling events in the BMP, WNT, and NODAL cascade during self-organized fate patterning in human gastruloids. PLoS Biol. *17*, e3000498. https://doi.org/10.1371/journal.pbio.3000498.

Chidiac, R., and Angers, S. (2023). Wnt signaling in stem cells during development and cell lineage specification. Curr. Top. Dev. Biol. *153*, 121–143. https://doi.org/10.1016/bs.ctdb.2023.01.005.

Chidiac, R., Abedin, M., Macleod, G., Yang, A., Thibeault, P.E., Blazer, L.L., Adams, J.J., Zhang, L., Roehrich, H., Jo, H.-N., et al. (2021). A Norrin/Wnt surrogate antibody stimulates endothelial cell barrier function and rescues retinopathy. EMBO Mol. Med. *13*, e13977. https://doi.org/10.15252/emmm.202113977.

Cohen, D.E., and Melton, D. (2011). Turning straw into gold: directing cell fate for regenerative medicine. Nat. Rev. Genet. *12*, 243–252. https://doi.org/10.1038/nrg2938.

Fernandez, A., Huggins, I.J., Perna, L., Brafman, D., Lu, D., Yao, S., Gaasterland, T., Carson, D.A., and Willert, K. (2014). The WNT receptor FZD7 is required for maintenance of the pluripotent state in human embryonic stem cells. Proc. Natl. Acad. Sci. USA *111*, 1409–1414. https://doi.org/10.1073/pnas.1323697111.

Fowler, J.L., Ang, L.T., and Loh, K.M. (2020). A critical look: Challenges in differentiating human pluripotent stem cells into desired cell types and organoids. Wiley Interdiscip. Rev. Dev. Biol. *9*, e368. https://doi.org/10.1002/wdev.368.

Gumber, D., Do, M., Suresh Kumar, N., Sonavane, P.R., Wu, C.C.N., Cruz, L.S., Grainger, S., Carson, D., Gaasterland, T., and Willert, K. (2020). Selective activation of FZD7 promotes mesendodermal differentiation of human pluripotent stem cells. Elife *9*, e63060. https://doi.org/10.7554/eLife.63060.

Hu, S., Liu, S., Bian, Y., Poddar, M., Singh, S., Cao, C., McGaughey, J., Bell, A., Blazer, L.L., Adams, J.J., et al. (2022). Single-cell spatial transcriptomics reveals a dynamic control of metabolic zonation and liver regeneration by endothelial cell Wnt2 and Wnt9b. Cell Rep. Med. *3*, 100754. https://doi.org/10.1016/j.xcrm.2022.100754.

Huggins, I.J., Bos, T., Gaylord, O., Jessen, C., Lonquich, B., Puranen, A., Richter, J., Rossdam, C., Brafman, D., Gaasterland, T., and Willert, K. (2017). The WNT target SP5 negatively regulates WNT transcriptional programs in human pluripotent stem cells. Nat. Commun. *8*, 1034. https://doi.org/10.1038/s41467-017-01203-1.

Janda, C.Y., Dang, L.T., You, C., Chang, J., de Lau, W., Zhong, Z.A., Yan, K.S., Marecic, O., Siepe, D., Li, X., et al. (2017). Surrogate Wnt agonists that phenocopy canonical Wnt and β-catenin signalling. Nature *545*, 234–237. https://doi.org/10.1038/nature22306.

Kattman, S.J., Witty, A.D., Gagliardi, M., Dubois, N.C., Niapour, M., Hotta, A., Ellis, J., and Keller, G. (2011). Stage-specific optimization of activin/nodal and BMP signaling promotes cardiac differentiation of mouse and human pluripotent stem cell lines. Cell Stem Cell *8*, 228–240. https://doi.org/10.1016/j.stem.2010.12.008.

Kim, M.-S., Horst, A., Blinka, S., Stamm, K., Mahnke, D., Schuman, J., Gundry, R., Tomita-Mitchell, A., and Lough, J. (2015). Activin-A and Bmp4 levels modulate cell type specification during CHIR-induced cardiomyogenesis. PLoS One *10*, e0118670. https://doi.org/10.1371/journal.pone.0118670.

Kispert, A., and Herrmann, B.G. (1994). Immunohistochemical analysis of the Brachyury protein in wild-type and mutant mouse embryos. Dev. Biol. *161*, 179–193. https://doi.org/10.1006/dbio.1994.1019.

Lian, X., Zhang, J., Azarin, S.M., Zhu, K., Hazeltine, L.B., Bao, X., Hsiao, C., Kamp, T.J., and Palecek, S.P. (2013). Directed cardiomyocyte differentiation from human pluripotent stem cells by modulating Wnt/β-catenin signaling under fully defined conditions. Nat. Protoc. *8*, 162–175. https://doi.org/10.1038/nprot.2012.150.

Loh, K.M., Ang, L.T., Zhang, J., Kumar, V., Ang, J., Auyeong, J.Q., Lee, K.L., Choo, S.H., Lim, C.Y.Y., Nichane, M., et al. (2014). Efficient endoderm induction from human pluripotent stem cells by logically directing signals controlling lineage bifurcations. Cell Stem Cell *14*, 237–252. https://doi.org/10.1016/j.stem.2013.12.007.

Loh, K.M., Chen, A., Koh, P.W., Deng, T.Z., Sinha, R., Tsai, J.M., Barkal, A.A., Shen, K.Y., Jain, R., Morganti, R.M., et al. (2016). Mapping the Pairwise Choices Leading from Pluripotency to Human Bone, Heart, and Other Mesoderm Cell Types. Cell *166*, 451–467. https://doi.org/10.1016/j.cell.2016.06.011.

Martyn, I., Kanno, T.Y., Ruzo, A., Siggia, E.D., and Brivanlou, A.H. (2018). Self-organization of a human organizer by combined Wnt and Nodal signalling. Nature *558*, 132–135. https://doi.org/10.1038/s41586-018-0150-y.

Massey, J., Liu, Y., Alvarenga, O., Saez, T., Schmerer, M., and Warmflash, A. (2019). Synergy with TGFβ ligands switches WNT pathway dynamics from transient to sustained during human pluripotent cell differentiation. Proc. Natl. Acad. Sci. USA *116*, 4989–4998. https://doi.org/10.1073/pnas.1815363116.

Murry, C.E., and Keller, G. (2008). Differentiation of embryonic stem cells to clinically relevant populations: lessons from embryonic development. Cell *132*, 661–680. https://doi.org/10.1016/j.cell.2008.02.008.

Nabhan, A.N., Webster, J.D., Adams, J.J., Blazer, L., Everrett, C., Eidenschenk, C., Arlantico, A., Fleming, I., Brightbill, H.D., Wolters, P.J., et al. (2023). Targeted alveolar regeneration with Frizzled-specific agonists. Cell *186*, 2995–3012.e15. https://doi.org/10.1016/j.cell.2023.05.022.

Nakamura, Y., Tsiairis, C.D., Özbek, S., and Holstein, T.W. (2011). Autoregulatory and repressive inputs localize Hydra Wnt3 to the head organizer. Proc. Natl. Acad. Sci. USA *108*, 9137–9142. https://doi.org/10.1073/pnas.1018109108.

Nostro, M.C., Cheng, X., Keller, G.M., and Gadue, P. (2008). Wnt, activin, and BMP signaling regulate distinct stages in the developmental pathway from embryonic stem cells to blood. Cell Stem Cell *2*, 60–71. https://doi.org/10.1016/j.stem.2007.10.011.

Osnato, A., Brown, S., Krueger, C., Andrews, S., Collier, A.J., Nakanoh, S., Quiroga Londoño, M., Wesley, B.T., Muraro, D., Brumm, A.S., et al. (2021). TGFβ signalling is required to maintain pluripotency of human naïve pluripotent stem cells. Elife *10*, e67259. https://doi.org/10.7554/eLife.67259.

Pavlovic, Z., Adams, J.J., Blazer, L.L., Gakhal, A.K., Jarvik, N., Steinhart, Z., Robitaille, M., Mascall, K., Pan, J., Angers, S., et al. (2018). A synthetic anti-Frizzled antibody engineered for broadened specificity exhibits enhanced anti-tumor properties. mAbs *10*, 1157–1167. https://doi.org/10.1080/19420862.2018.1515565.

Rao, J., Pfeiffer, M.J., Frank, S., Adachi, K., Piccini, I., Quaranta, R., Araúzo-Bravo, M., Schwarz, J., Schade, D., Leidel, S., et al. (2016). Stepwise clearance of repressive roadblocks drives cardiac induction in human ESCs. Cell Stem Cell *18*, 554–556. https://doi.org/10.1016/j.stem.2016.03.008.

Rojas, A., De Val, S., Heidt, A.B., Xu, S.-M., Bristow, J., and Black, B.L. (2005). Gata4 expression in lateral mesoderm is downstream of BMP4 and is activated directly by Forkhead and GATA transcription factors through a distal enhancer element. Development *132*, 3405–3417. https://doi.org/10.1242/dev.01913.

Sadahiro, T., Isomi, M., Muraoka, N., Kojima, H., Haginiwa, S., Kurotsu, S., Tamura, F., Tani, H., Tohyama, S., Fujita, J., et al. (2018). Tbx6 induces nascent mesoderm from pluripotent stem cells and temporally controls cardiac versus somite lineage diversification. Cell Stem Cell *23*, 382–395.e5. https://doi.org/10.1016/j.stem.2018.07.001.

Sagara, N., Toda, G., Hirai, M., Terada, M., and Katoh, M. (1998). Molecular cloning, differential expression, and chromosomal localization of human frizzled-1, frizzled-2, and frizzled-7. Biochem. Biophys. Res. Commun. *252*, 117–122. https://doi.org/10.1006/bbrc.1998.9607.

Söderholm, S., Jauregi-Miguel, A., Pagella, P., Ghezzi, V., Zambanini, G., Nordin, A., and Cantù, C. (2023). Single-cell response to Wnt signaling activation reveals uncoupling of Wnt target gene expression. Exp. Cell Res. *429*, 113646. https://doi.org/10.1016/j.yexcr.2023.113646.

Solnica-Krezel, L., and Sepich, D.S. (2012). Gastrulation: making and shaping germ layers. Annu. Rev. Cell Dev. Biol. *28*, 687–717. https://doi.org/10.1146/annurev-cellbio-092910-154043.

Steinhart, Z., and Angers, S. (2018). Wnt signaling in development and tissue homeostasis. Development *145*, dev146589. https://doi.org/10.1242/dev.146589.

Steinhart, Z., Pavlovic, Z., Chandrashekhar, M., Hart, T., Wang, X., Zhang, X., Robitaille, M., Brown, K.R., Jaksani, S., Overmeer, R., et al. (2017). Genome-wide CRISPR screens reveal a Wnt–FZD5 signaling circuit as a druggable vulnerability of RNF43-mutant pancreatic tumors. Nat. Med. *23*, 60–68. https://doi.org/10.1038/nm.4219.

Sumi, T., Tsuneyoshi, N., Nakatsuji, N., and Suemori, H. (2008). Defining early lineage specification of human embryonic stem cells by the orchestrated balance of canonical Wnt/beta-catenin, Activin/Nodal and BMP signaling. Development *135*, 2969–2979. https://doi.org/10.1242/dev.021121.

Tan, J.Y., Sriram, G., Rufaihah, A.J., Neoh, K.G., and Cao, T. (2013). Efficient derivation of lateral plate and paraxial mesoderm subtypes from human embryonic stem cells through GSKi-mediated differentiation. Stem Cells Dev. *22*, 1893–1906. https://doi.org/10.1089/scd.2012.0590.

Tani, S., Chung, U.-I., Ohba, S., and Hojo, H. (2020). Understanding paraxial mesoderm development and sclerotome specification for skeletal repair. Exp. Mol. Med. *52*, 1166–1177. https://doi.org/10.1038/s12276-020-0482-1.

Tao, Y., Mis, M., Blazer, L., Ustav, M., Jnr, Steinhart, Z., Chidiac, R., Kubarakos, E., O'Brien, S., Wang, X., Jarvik, N., et al. (2019). Tailored tetravalent antibodies potently and specifically activate Wnt/Frizzled pathways in cells, organoids and mice. Elife *8*, e46134. https://doi.org/10.7554/eLife.46134.

Tsaytler, P., Liu, J., Blaess, G., Schifferl, D., Veenvliet, J.V., Wittler, L., Timmermann, B., Herrmann, B.G., and Koch, F. (2023). BMP4 triggers regulatory circuits specifying the cardiac mesoderm lineage. Development *150*, dev201450. https://doi.org/10.1242/dev.201450.

Wierson, W.A., Welker, J.M., Almeida, M.P., Mann, C.M., Webster, D.A., Torrie, M.E., Weiss, T.J., Kambakam, S., Vollbrecht, M.K., Lan, M., et al. (2020). Efficient targeted integration directed by short homology in zebrafish and mammalian cells. eLife *9*, e53968. https://doi.org/10.7554/eLife.53968.

Wu, Z., Shen, S., Mizikovsky, D., Cao, Y., Naval-Sanchez, M., Tan, S.Z., Alvarez, Y.D., Sun, Y., Chen, X., Zhao, Q., et al. (2024). Wnt dose escalation during the exit from pluripotency identifies tranilast as a regulator of cardiac mesoderm. Dev. Cell *59*, 705–722.e8. https://doi.org/10.1016/j.devcel.2024.01.019.

Xu, P.-F., Houssin, N., Ferri-Lagneau, K.F., Thisse, B., and Thisse, C. (2014). Construction of a vertebrate embryo from two opposing morphogen gradients. Science *344*, 87–89. https://doi.org/10.1126/science.1248252.

Yamaguchi, T.P., Takada, S., Yoshikawa, Y., Wu, N., and McMahon, A.P. (1999). T (Brachyury) is a direct target of Wnt3a during paraxial mesoderm specification. Genes Dev. *13*, 3185–3190. https://doi.org/10.1101/gad.13.24.3185.

Yang, A., Chidiac, R., Russo, E., Steenland, H., Pauli, Q., Bonin, R., Blazer, L.L., Adams, J.J., Sidhu, S.S., Goeva, A., et al. (2024). Exploiting spatiotemporal regulation of FZD5 during neural patterning for efficient ventral midbrain specification. Development *151*, dev202545. https://doi.org/10.1242/dev.202545.

Yu, H., Ye, X., Guo, N., and Nathans, J. (2012). Frizzled 2 and frizzled 7 function redundantly in convergent extension and closure of the ventricular septum and palate: evidence for a network of interacting genes. Development *139*, 4383–4394. https://doi.org/10.1242/dev.083352.

Zhao, M., Tang, Y., Zhou, Y., and Zhang, J. (2019). Deciphering Role of Wnt Signalling in Cardiac Mesoderm and Cardiomyocyte Differentiation from Human iPSCs: Four-dimensional control of Wnt pathway for hiPSC-CMs differentiation. Sci. Rep. *9*, 19389. https://doi.org/10.1038/s41598-019-55620-x.

Stem Cell Reports, Volume *20*

Supplemental Information

Selective activation of FZD2 and FZD7 reveals non-redundant function during mesoderm differentiation

Rony Chidiac, Andy Yang, Elli Kubarakos, Nicholas Mikolajewicz, Hong Han, Maira P. Almeida, Pierre E. Thibeault, Sichun Lin, Graham MacLeod, Jean-Philippe Gratton, Jason Moffat, and Stephane Angers

Supplementary Figure 1

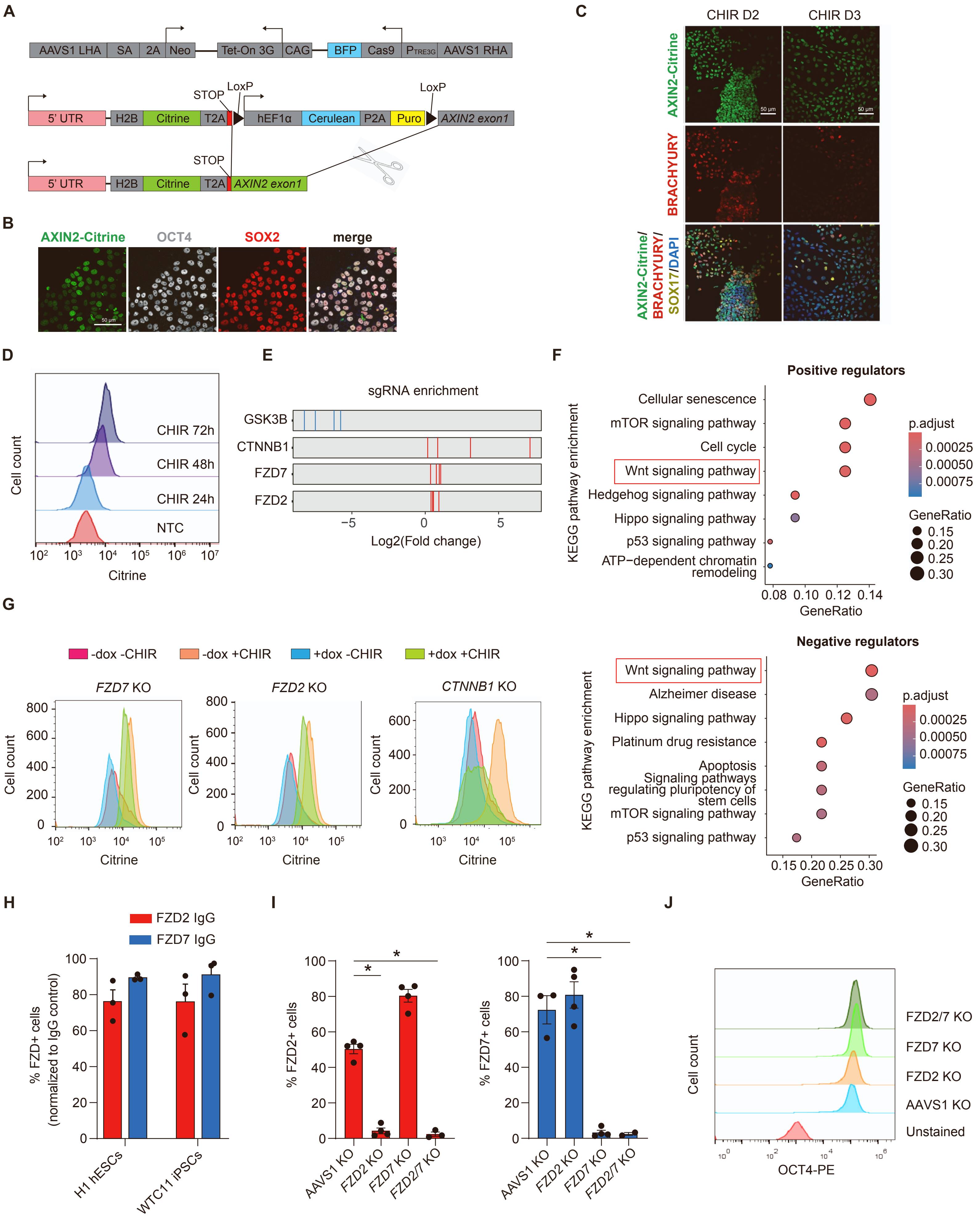

Supplementary Figure 2

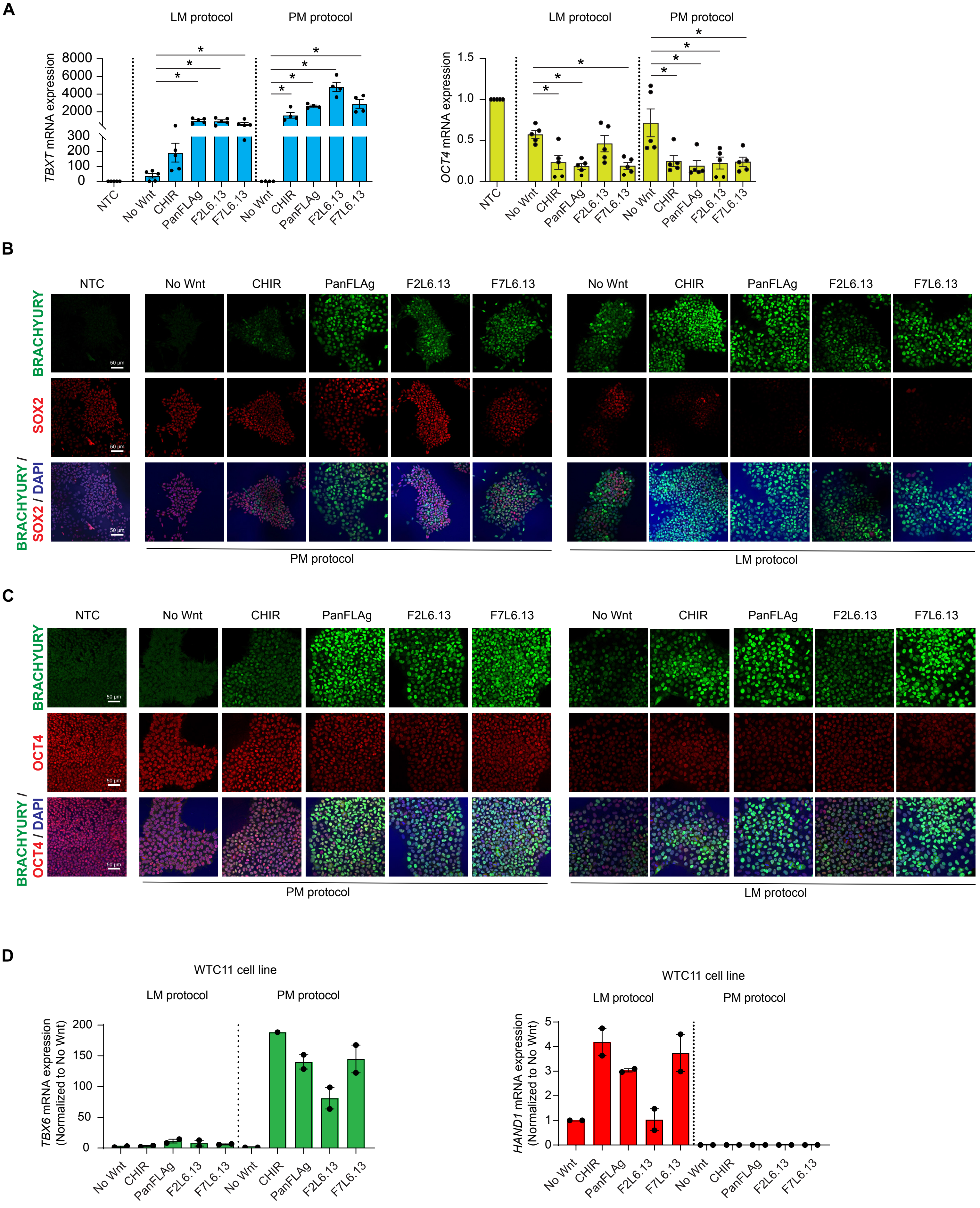

Supplementary Figure 3

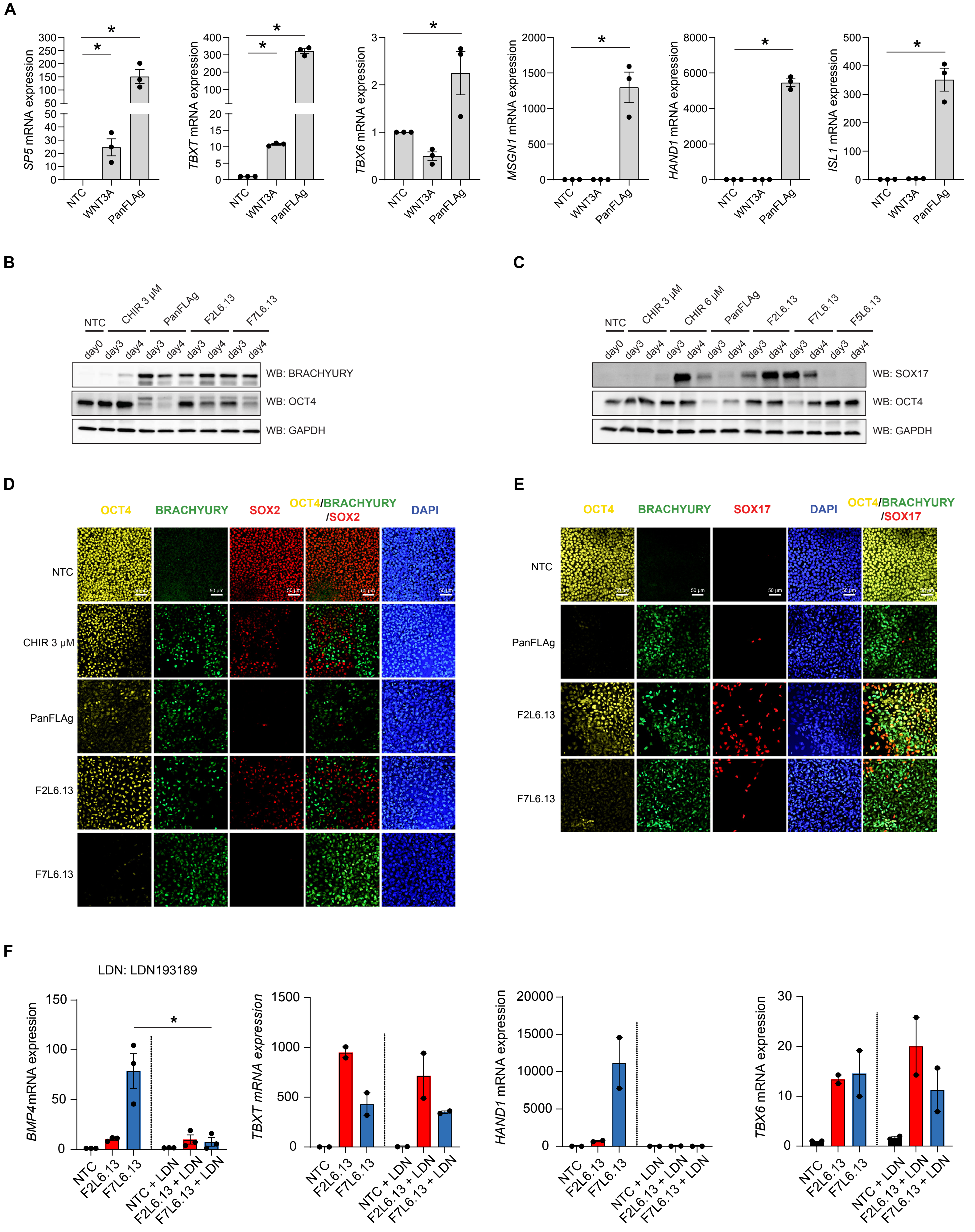

Supplementary Figure 4

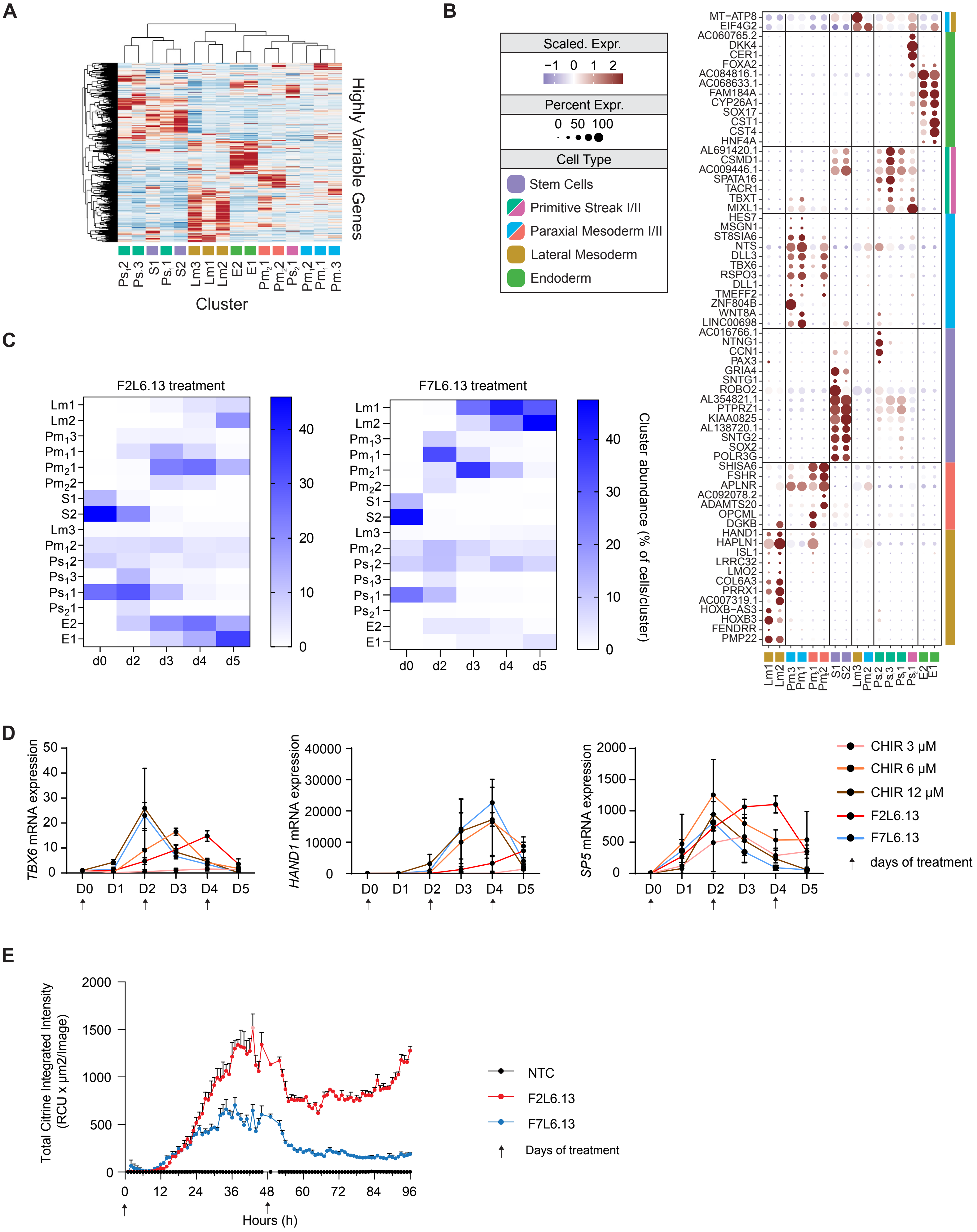

Supplementary Figure 5

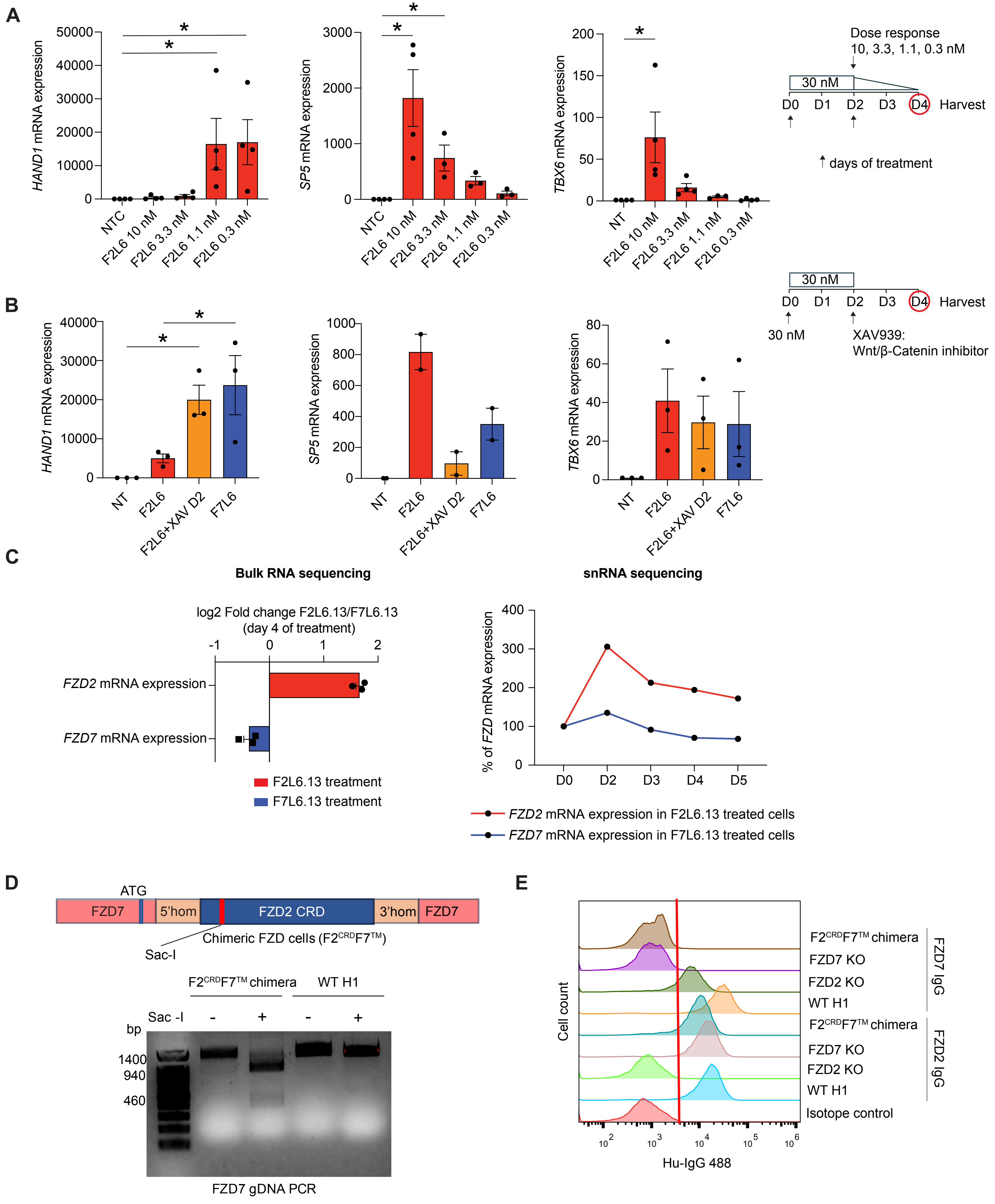

Supplementary Figures legends

Figure S1: Genome-wide CRISPR screen showing non-redundant functions for FZD2 and FZD7 during Wnt activation in hPSCs, related to Figure 1.

A) Left and right homology arms (LHA and RHA, respectively) targeting the AAVS1 safe-harbour locus allowed for integration of a Cas9-Blue fluorescent protein (BFP) cassette driven by an inducible Tet-On 3G system, coupled with a neomycin (Neo) selection cassette (cell line commissioned by the Centre for Commercialization of Regenerative Medicine, CCRM). Left and right homology arms (LHA and RHA, respectively) were cloned using restriction enzymes into a pre-constructed vector containing a nuclear H2B-tagged citrine fluorescent protein, with a constitutive hEF1α-driven cerulean fluorescent protein and puromycin (Puro) resistant cell selection cassette flanked with LoxP excision sites.

B) Immunostaining against Citrine, OCT4 and SOX2 expression in the H1 iCas9 *AXIN2*-Citrine reporter line. Images are representative of two independent experiments.

C) Immunostaining against AXIN2-Citrine, BRACHYURY and SOX17 in the H1 iCas9 *AXIN2*-Citrine cells treated with 3 μM CHIR99021 for 3-4 days. Images are representative of two independent experiments.

D) Flow cytometry profiles of H1 iCas9 *AXIN2*-citrine reporter treated with 3 μM CHIR99021 for 24-72 hrs validating the responsiveness to Wnt activation. Data are representative histogram plots from two independent experiments.

E) The individual gRNA enrichment of GSK3β, CTNNB1, FZD2 and FZD7 genes is shown.

F) Gene ontology (GO) enrichment analysis plots of negative and positive regulators identified in our CRISPR screen.

G) Flow cytometry density plots of AXIN2-Citrine expression in H1 iCas9 *AXIN2*-Citrine knockout of positive regulators *FZD2*, *FZD7*, and *CTNNB1* (βcatenin) treated with 3 μM CHIR99021 for 72 hrs. Data are representative histogram plots from two independent experiments.

H) FZDs expression profiling by flow cytometry in H1 hESCs or WTC11 iPSCs using specific FZD2 or FZD7 specific IgG antibodies. Data are presented as mean ± SEM, n = 3 independent experiments.

I) Knockout FZD2, FZD7 or both in H1 hESCs. Flow cytometry shows the expression of FZD2 and FZD7 in each KO line. Data are presented as mean ± SEM, n = 4 independent experiments. Statistical analysis was performed using one-way ANOVA followed by Tukey's post hoc test. $*P \leq 0.05$ was considered significant.

J) Flow cytometry profile of OCT4 expression showing that KO lines maintain their undifferentiated state. Data are representative histogram plots from two independent experiments.

Figure S2: Mesoderm differentiation using F2L6.13 and F7L6.13 agonists, related to figure 2.

A) RT-qPCR of primitive streak (Left, *TBXT*) or pluripotency (Right, *OCT4*) marker at day 1 of differentiation protocol adapted from Loh et al. 2016. Data are presented as mean ± SEM, n = 5 independent differentiation experiments. Statistical analysis was performed using one-way ANOVA followed by Tukey's post hoc test. $*P \leq 0.05$ was considered significant.

B, C) Immunostaining against BRACHYURY (B-C), SOX2 (B) and OCT4 (C) in paraxial versus lateral mesoderm differentiation protocol (day 1) in hESCs. Images are representative of three independent differentiation experiments.

D) RT-qPCR of WTC11-derived paraxial mesoderm markers (*TBX6*) or lateral mesoderm markers (*HAND1*) at day 2 of differentiation. Data are presented as mean ± SEM, n = 2 independent differentiation experiments.

Figure S3: Examining Wnt-mediated mesoderm differentiation using Wnt ligands, CHIR99021 or various FLAgs, related to figure 3.

A) RT-qPCR of a panel of differentiation markers in H1 hESCs after 4 days of treatment with 300 ng/ml of purified Wnt3a protein or 30 nM of PanFLAg. Data are presented as mean ± SEM, n=3 independent differentiation experiments. Statistical analysis was performed using one-way ANOVA followed by Tukey's post hoc test. $*P \leq 0.05$ was considered significant.

B) Western blot analysis of BRACHYURY and OCT4 expression in H1 hESCs treated with CHIR99021 or FLAgs. GAPDH was used as a loading control. Representative blots of three independent experiments are shown.

C) Western blot analysis of SOX17 and OCT4 expression in H1 hESCs treated with CHIR99021 or FLAgs. GAPDH was used as a loading control. Representative blots of two independent experiments are shown.

D) Immunofluorescence of BRACHYURY, OCT4 and SOX2 in H1 hESCs treated with CHIR99021 or FLAgs for 4 days. Images are representative of three independent differentiation experiments.

E) Immunofluorescence of BRACHYURY, OCT4 and SOX17 in H1 hESCs treated with CHIR99021 or FLAgs for 4 days. Images are representative of three independent differentiation experiments.

F) RT-qPCR of *BMP4*, *TBXT*, *TBX6,* and *HAND1* mRNA expression in H1 hESC treated with 30 nM of F2L6.13 and F7L6.13 in the presence or absence of BMP4 inhibitor LDN193189 (LDN) for 4 days. Data are presented as mean ± SEM, n = 2-3 independent experiments. For *BMP4* mRNA expression (n = 3), statistical analysis was performed using one-way ANOVA followed by Tukey's post hoc test. *$P \leq 0.05$ was considered significant.

Figure S4: Single-nucleus RNA sequencing analysis and kinetic profiles of Wnt target genes in F2L6.13 and F7L6.13 treated hESCs, related to figures 4 and 5.

A) Hierarchical clustering of highly variable gene expression in each cellular subpopulation. Stem cells (S1-2), primitive streak ($Ps_1$1-3 and $Ps_2$1), paraxial ($Pm_1$1-3 and $Pm_2$1-2) and lateral (Lm1-3) mesoderm, and endoderm (E1-2).

B) Dot plot of differentially expressed gene markers. Scaled average expression and percent of cells expressing the indicated gene were indicated by dot color and size, respectively. Temporal classes with colors corresponding to the identity of each cluster.

C) Heatmap of cluster abundance measuring the percentage of cells in each cluster of F2L6.13 (Left) versus F7L6.13 (Right) treated H1 hESCs. Data are presented as means, n = 2 independent differentiation experiments/timepoint/treatment.

D) Time course gene expression of *SP5*, *TBX6*, and *HAND1* markers in hESCs treated with the indicated concentration of CHIR99021 or 30 nM of F2L6.13 and F7L6.13 for 5 days. Data are presented as mean ± SEM. Experiment was repeated twice with similar results.

E) Quantification of time-lapse imaging of AXIN2-Citrine fluorescence intensity over 4 days of differentiation (mean ± SEM of 3 different images per treatment). Experiment was repeated twice with similar results

Figure S5: Effect of modulating Wnt activation kinetics on mesoderm formation, related to figure 5.

A) Dose-response RT-qPCR analysis of *SP5*, *TBX6*, and *HAND1* markers in hESCs treated with a maximal dose of 30 nM F2L6.13 followed with different concentrations of F2L6.13 starting on day 2 and harvested at day 4 of treatment. Data are presented as mean ± SEM, n = 4 independent experiments. Statistical analysis was performed using one-way ANOVA followed by Tukey's post hoc test. *$P \leq 0.05$ was considered significant.

B) RT-qPCR analysis of *SP5*, *TBX6*, and *HAND1* markers in hESCs treated with a maximal dose of 30 nM F2L6.13 followed with 2 μM XAV939 on day 2 and harvested at day 4 of treatment. Data are presented as mean ± SEM, n = 2-3 independent experiments. For *HAND1* mRNA expression (n = 3), statistical analysis was performed using one-way ANOVA followed by Tukey's post hoc test. *$P \leq 0.05$ was considered significant.

C) *FZD2* and *FZD7* expression in bulk and snRNA sequencing data. (Right) *FZD2* and *FZD7* mRNA expression in F2L6.13 and F7L6.13-treated cells were determined by calculating the fold

change between F2L6.13 and F7L6.13 treatment. (Left) *FZD2* and *FZD7* mRNA expression is shown as a percentage relative to baseline expression at day 0.

D) Schematic of $F2^{CRD}F7^{TM}$ chimera design (top) in which the coding sequence for the FZD2 CRD replaced that of the FZD7 CRD at the *FZD7* locus. SacI is a restriction enzyme that cuts uniquely in the FZD2 CRD. PCR (bottom) using either WT H1 or $F2^{CRD}F7^{TM}$ chimeric cells in which SacI enzyme was used to cut inside the FZD2 CRD confirming the chimeric line and showing that the line is homozygous.

E) Flow cytometry showing FZD2 or FZD7 expression in WT H1 or $F2^{CRD}F7^{TM}$ chimeric cells using specific FZD2 or FZD7 IgGs. Data are representative histogram plots from two independent experiments.

Supplemental experimental procedures

Maintenance of hPSCs

H1 hESCs and WTC11 hiPSCs were cultured on Geltrex-coated (Gibco, A1413302, 1:100 in DMEM/F12; Gibco, 11320082) plates and maintained in StemFlex basal medium (Gibco, A3349401) supplemented with 1% Penicillin-Streptomycin (Gibco 10,000 U/mL, 15-140-122) at 37 °C in a humidified CO2 incubator. For maintenance, cells were passaged every 3-4 days at a 1:10 split ratio with Versene (Gibco, 15040066). For experiments, cells were washed once with PBS (Gibco, 14-190-250) and dissociated using TrypLE Select enzyme (Gibco, 12-604-021) and neutralized using 10% FBS (Gibco, 12483020) in DMEM/F12. Cells were then plated onto Geltrex-coated plates in StemFlex supplemented with 1X RevitaCell (Gibco 100X, A2644501). RevitaCell was removed the next day and the media was changed every 2 days for cell maintenance. All differentiations were conducted on authenticated and G-banded karyotyped H1 or WTC11 cells grown in feeder-free and monolayer conditions in StemFlex media and experiments were conducted within 10 passages. Daily observation under the microscope was done to monitor morphology, infection or any sort of contamination. Cells were frozen using the PSC cryopreservation kit from ThermoFisher (A2644601). hPSC lines were tested routinely for mycoplasma contamination using the MycoAlert Plus detection kit (Lonza).

Mesoderm, cardiac and cardiomyocyte cell differentiation

hPSCs were differentiated into mesoderm and downstream lineages as previously described (Loh et al. 2016). For paraxial and lateral mesoderm-directed differentiation, hPSCs were briefly washed and then differentiated into either anterior primitive streak (30 ng/mL Activin A, 4 μM

CHIR99021, 20 ng/mL FGF2, 100 nM PIK90) or mid primitive streak (30 mg/mL Activin A, 40 ng/mL BMP4, 6 μM CHIR99021, 20 ng/mL FGF2, 100 nM PIK90) for 24 hrs. Subsequently, day 1 anterior primitive streak was differentiated towards day 2 paraxial mesoderm for 24 hrs (1 μM A-83-01, 3 μM CHIR99021, 250 nM LDN-193189, 20 ng/mL FGF2). Separately, day 1 mid-primitive streak was differentiated towards day 2 lateral mesoderm for 24 hrs (1 μM A-83-01, 30 ng/mL BMP4, 1 μM C59).

For cardiac mesoderm and cardiomyocytes differentiation, day 2 lateral mesoderm was differentiated into day 4 cardiac mesoderm by treating them with 1 μM A8301, 30 ng/mL BMP4, 1 μM C59, 20 ng/mL FGF2 for 48 hrs. Subsequently, day 4 cardiac mesoderm was treated with 30 ng/mL BMP4, 1 μM XAV939, 200 μg/mL 2-phospho-ascorbic acid (Sigma) for 96 hrs to yield day 8 cardiomyocyte-containing populations. Media was changed 24 hrs for all steps.

H1 *AXIN2*-Citrine reporter cell line

The H1 iCas9 cells were generated from the H1 hESC parental line Centre for Commercialization of Regenerative Medicine (Toronto, Canada) by insertion of a vector sequence containing the inducible TRE3G-Cas9-P2A-BFP2 and constitutive Tet-On 3G expression cassettes into the safe harbor locus AAVS1 using CRISPR (Supplementary Figure S1a). H1 iCas9 cells were engineered to incorporate an H2B-tagged Citrine fluorescent protein before the first exon of the *AXIN2* locus. To do so, we used the reporter backbone TNTDNA104-pKW1-H2B-Citrine, a generous gift from the Ajamete Kaykas Lab (Novartis) which has a constitutive hEF1α-driven cerulean fluorescent protein and puromycin (Puro) resistant cell selection cassette flanked with LoxP excision sites allowing for cells to be selected for successful integration which is shown by PCR (Supplementary Figure 1A, 1B). The nickase Cas9 (nuclease deficient Cas9 D10A) and two sgRNA were used to introduce a double-stranded break upstream of the first exon of the *AXIN2* locus. Electroporation

of the sgRNA and a donor template (containing the H2B-Citrine) into H1 iCas9 cells incurred a DSB and successful insertion of the Citrine cassette in the *Axin2* locus. $1x10^6$ H1 iCas9 hESCs were electroporated with 2 µg of each *Axin2* Nickase sgRNA (Table S3) and 2 µg of the donor vector (containing the H2B-Citrine). Cells were electroporated using the Neon Transfection System (ThermoFisher), 100 µL Neon Tips (ThermoFisher) and following the electroporation parameters: 1050 V, 30 ms pulse width, 2 pulses. Electroporated cells were plated in 6-well plates with prewarmed StemFlex media supplemented with Revitacell. Following 2 days of puromycin treatment (2 µg/mL), cell colonies were picked manually and re-plated in individual 24-wells with Revitacell and expanded with Versene (Gibco) until they could be harvested and frozen for characterization. PCR and randomly integrated clones were excluded from subsequent experiments. PCR also confirmed the integration was heterozygous as a WT allele was still intact (Supplementary Figure 1D), and upon manual clonal selection and expansion, cells displayed heterogenous citrine expression.

Following clonal selection, the hEF1α-Cerulean-Puromycin cassette was excised using the Cre-LoxP system with the electroporation of 2.5 µg of Cre-mRNA (TriLink Biotechnologies, Cat. L-7211) per 500,000 cells. We confirmed that the clone had resumed puromycin sensitivity, Then, to ensure the removal of the Cerulean-Puromycin cassette and single-cell clonality, cells were sorted via fluorescence-activated cell sorting (FACS Aria III, BD BioSciences) and clones were further expanded. To assess whether pluripotency had been affected in the editing process, we characterized the undifferentiated cell state using OCT4 and SOX2 markers.

sgRNA lentivirus production and infection

HEK 293T cells were transfected at approximately 60% confluency with 5 µg of a psPAX2 packaging plasmid, 2 µg of a VSV.G enveloping plasmid and 5 µg of the intended lentiviral

construct cloned with the gene of interest (using a pLCKO backbone; addgene, 73311) in 250 µL of OptiMEM (Gibco, 31985070) reduced serum media. A 3:1 ratio of PEI to DNA was prepared separately, diluted in OptiMEM and briefly vortexed. The transfection reagent was transferred to the tube containing DNA and incubated for 20 min, then added dropwise to cell culture plates. The following day, the media was changed to regular DMEM containing 10% FBS and 1% PenStrep, and 24 hrs later that media was harvested and spun down (2000 x g for 2 minutes). The supernatant was filtered with a 0.4 µm filter and Lenti-X Concentrator (Takara Bio, 631232) was added to the media in a 1:3 volume ratio. Tubes were rotated for 2 hrs at 4 °C then spun at 1500 x g for 45 min at 4 °C. The pellets were resuspended in 250 µL of DMEM/F12, aliquoted and stored at -80 °C for long-term storage. Infection of hESCs was done in regular StemFlex media, the day after seeding. Media was changed before infection with 3-5 µL of concentrated virus. Media was changed 24 hrs later, then cells were selected with puromycin (2 µg/ml) for 2 days of selection. Cas9 editing was then induced by adding doxycycline (1.5 µg/ml) (Biobasic, DB0889) to media for 3-4 days in infected lines. Samples were Sanger sequenced using respective forward TIDE primers and INDELs and sgRNA cutting efficiency was measured by Tracking of Indels by DEcomposition (TIDE) analysis. Primers and sgRNA sequences were added to Table S3.

CRISPR Cas9 Screen

H1 iCas9 *AXIN2*-Citrine cells (passage number 75) were seeded in 15 cm plates with RevitaCell-supplemented StemFlex media. The following day cells were infected with the TKOv3 virus (71,090 sgRNAs) (addgene pooled library #90924) (Hart et al. 2017) at a MOI of 0.3 and library coverage of 400-fold. After 24 hrs, media was changed and cells were subjected to 48 hrs of puromycin selection (2 µg/mL). After splitting cells, and maintaining fold coverage, cells were treated with doxycycline (1.5 µg/mL) for 4 days. Following doxycycline selection, cells were split

into 3 biological replicates and treated with a final concentration of 3 μM of CHIR99021 for 3 days. Cells were harvested, resuspended in FACS buffer (5 mM EDTA, 25 mM HEPES, 1% FractionV BSA, in DPBS) and then stained with live/dead stain eBioscience Fixable Viability Dye eFluor 450 (Invitrogen, 65-0863-18) for 30 min on ice in the dark, fixed with 1% paraformaldehyde (PFA 32%, EMD 15714-S) for 20 min., then resuspended in FACS buffer. Samples were stored in the dark at 4 °C in FACS buffer until sorted. Cells were sorted for the top 15% and bottom 15% of citrine-expressing cells per replicate on a BD Influx and BD FACS AriaIII (BD Biosciences). Genomic DNA was extracted, amplified and barcoded with Illumina TruSeq adapters. Samples were sent for next-generation sequencing. The analysis included filtering guides with low-read counts so there was no guide misrepresentation, normalization of read counts of sorted cells versus the unsorted control and setting the default to 200 reads per sgRNA. The false discovery rate (FDR) was calculated for each individual sgRNA hit, -log10 transformed and ranked. Top 15% and bottom 15% mean counts across replicates were pooled and plotted against each other (log2 fold change "top15/bottom15"). KEGG pathway enrichment analysis was performed by taking the top list of genes from the top 15% and bottom 15% ranked list of hits with an FDR < 0.1.

Generation of a WTC11 SP5 reporter cell line

The pGTag-NLS-eGFP-SV40 vector (Addgene #117811) was adapted to apply the GeneWeld method for generating the *SP5-2A-mCherry* reporter allele using the H1 hESCs (Wierson et al. 2020). This CRISPR/Cas9 precision targeted integration strategy provides short homology arms that drive homology-mediated end joining at the cut site (Welker et al. 2021). The knock-in vector has a reporter cassette, which is composed of a self-cleaving 2A peptide from porcine teschvirus-1, a red fluorescent protein (mCherry), and a bovine growth hormone transcription termination and

polyadenylation sequence (2A-mCherry-BGHpolyA). The vector also has a selection marker consisting of a human elongation factor-1 alpha promoter followed by a puromycin resistance gene, and a rabbit beta-globulin termination and polyadenylation sequence (huEF1α-Puro-RBGpolyA). The presence of LoxP sites allows the removal of the selection marker using Cre recombinase. Flanking the knock-in sequence there are 48 bp homology arms corresponding to the genomic target site. Externally, there are universal sgRNA (UgRNA) sites for Cas9-induced double-strand breaks and consequent release of the knock-in sequence from the vector backbone and exposure of the short homology arms, which are important for integration. 5' and 3' homology arms were designed and cloned into the vector as described (Wierson et al. 2020).

To generate the reporter allele, the knock-in cassette was inserted between the last amino acid and the stop codon to guarantee the maintenance of SP5 endogenous expression. A genomic sgRNA sequence upstream of the stop codon was selected. The bases that would have been lost after the double-strand break induction were added to the end of the 5' homology arm. sgRNA and homology arm oligonucleotide sequences are listed in Table S3. Since we could not mutate the PAM without changing the amino acid, we created four silent mutations within the spacer sequence to avoid a second Cas9-induced DSB after precise integration. For SP5 targeted integration, the Neon Transfection System (Invitrogen) was used, and the electroporation mix contained 1 µg of SP5 sgRNA, 1 µg of UgRNA, 1.2 µg of Cas9 mRNA, and 1 µg of vector. 300,000 H1-hESCs were transfected at a time using 1450 V, 10 ms, and 3x pulse. Later, the polyclonal population was selected with puromycin, and the surviving cells went through the process of monoclonal line isolation followed by genotyping. Their undifferentiated state was confirmed by OCT4 and SOX2 staining.

FZD7 CRD replacement and generation of chimeric receptor

For replacing FZD7 CRD with FZD2 CRD, we created a knock-in vector compatible with the GeneWeld CRISPR/Cas9 precision targeted integration strategy[70]. Initially, two sgRNAs outside FZD7 CRD were selected for induction of DNA double-strand breaks and complete deletion of the genomic sequence between the guides. Next, we designed the donor vector to contain the FZD2 CRD, which is flanked by the 5' and 3' 48 bp homology arms corresponding to the two FZD7 genomic targeting sites. Homology arms were designed and cloned into the vector as described[71]. The bases that would have been lost after double-strand break induction were added back to both homology arms. To avoid a second Cas9-induced DSB after CRD replacement, we created six silent mutations within the sgRNA spacer sequences (Table S3). Additionally, universal sgRNA (UgRNA) sites present in the donor vector were responsible for inducing Cas9 double-strand breaks, releasing the knock-in sequence from the backbone, and exposing the short homology arms for precise integration. The Neon Transfection System (Invitrogen) was used to deliver 1 µg of FZD7 sgRNA#1, 1 µg of FZD7 sgRNA#2, 1 µg of UgRNA, 1.2 µg of Cas9 mRNA, and 1 µg of vector. H1-hESCs FZD2$^{-/-}$ were transfected using 1450 V, 10 ms, and 3x pulse. Next, cells were sorted via fluorescence-activated cell sorting (FACS Aria III, BD BioSciences) using FZD2 antibodies and clones were further expanded. Precise integration was confirmed by genotyping and the undifferentiated state was validated by OCT4 and SOX2 staining.

Flow cytometry

Cells were washed with PBS once then harvested with TrypLE and STOP solution, and washed once with PBS following pelleting (1200 rpm, 4 min, 4°C). For endogenous fluorescence, cells were stained for 30 min on ice in the dark with a fixable viability dye eFluor 450 or eFluor 780, then fixed with 1% PFA or run directly on the CytoFLEX S (Beckman Coulter) flow cytometer. For intracellular staining, cells were harvested, washed once with PBS and stained with appropriate

eFluor. Following 1% PFA fixation (10 min on ice), cells were permeabilized with 0.3% Triton (Sigma) (5 min), washed once with PBS + 0.05% Triton, blocked for 1 h in 3% bovine serum albumin (BSA) (Gibco, Cat. 15260037) + 0.05% Triton, washed once, stained with the primary (or conjugated) antibody in 3% BSA + 0.05%Triton for 1 h in the dark on ice, washed twice with PBS. Secondary staining (if necessary) was done in 3% BSA + 0.05% Triton for 30 min on ice. Following 3 washes in PBS, cells were resuspended in the FACS buffer and run on the flow cytometer. For FZD membrane staining, cells were harvested, washed once with PBS and blocked with 3% BSA for 30 min on ice. Cells were stained with 100 nM of FZD IgGs in a blocking buffer for 1 h on ice. Cells were then washed 2 times and stained with secondary antibody AF488 anti-human in addition to eFluor780 for 30 min on ice in the dark. All FLOW cytometry FCS files were exported and analyzed on FlowJo Software.

RNA sequencing

H1 hESCs were differentiated as described above in three independent experiments and RNA was extracted using TRIzol reagent (Invitrogen) following the manufacturer's instructions. Sequencing libraries were prepared and run on an Illumina NextSeq-500 instrument at the Lunenfeld-Tanenbaum Research Institute Sequencing Facility (Toronto, ON) generating 75 bp single-read FASTQ files. The transcript reads from FASTQ files were aligned to the Homo Sapiens transcriptome and quantified using Kallisto (Bray et al. 2016), following default parameters. Differential expression analysis was performed using the R Bioconductor DESEq2 package (Bray et al. 2016; Love, Huber, and Anders 2014) using default parameters with the independent filtering option disabled, with thresholds of raw fold change > 1.5 and adjusted *P*-value < 0.1. Pathway enrichment analysis using a ranked gene list was performed using GSEA software (Subramanian

et al. 2005). Heatmaps and volcano plots of differentially expressed genes were generated using R packages.

Single-nucleus RNA-Seq Experiments and Analysis

Sample processing, sci-RNA-seq3 library generation, and sequencing: Cells were harvested with 0.25% trypsin-EDTA and neuron dissociation solution (Tian et al. 2019), respectively. Cell pellets were immediately snap-frozen in liquid nitrogen and then stored at -80 °C for sci-RNA-Seq3-based single-nucleus RNA-Seq processing. Samples from all conditions were processed together to minimize batch effects. Nuclei extraction and fixation were performed as previously described (Cao et al. 2019), except for the use of a modified CST lysis buffer (Slyper et al. 2020) plus 1% SUPERase In RNase Inhibitor (AM2696). Nuclei quality was checked with DAPI and Wheat Germ Agglutinin (WGA) staining. Sci-RNA-Seq3 libraries were generated as previously described (Cao et al. 2019) using three-level combinatorial indexing. The final libraries were sequenced on an Illumina NovaSeq 6000 using the following protocol: read 1: 34 bp, read 2: 69 bp, index 1: 10 bp, index 2: 10 bp. Raw sequencing reads were first demultiplexed based on i5/i7 PCR barcodes. FASTQ files were then processed using the sci-RNA-Seq3 pipeline (Cao et al. 2019). After barcodes and UMIs were extracted from the read1 FASTQ files, read alignment was performed using the STAR short-read aligner (v2.5.2b) with the human genome (hg38) and Gencode v25 gene annotations. After removing duplicate reads based on UMI, barcode, chromosome and alignment position, reads are summarized into a count matrix of M genes x N nuclei.

***Filtering*:** Raw single-cell gene count matrices were loaded into a *Seurat* object (version 4.0.4) (Butler et al. 2018; Hao et al. 2021; Satija et al. 2015; Stuart et al. 2019) and filtered to retain cells

with (*i*) 200 – 9000 recovered genes per cell, (*ii*) less than 60% mitochondrial content, and (*iii*) unmatched rate within 3 median absolute deviations of the median.

Normalization: To normalize expression values, we adopted the modeling framework previously described and implemented in the *sctransform* R Package (version 0.3.2) (Hafemeister and Satija 2019). In brief, count data were modelled by regularized negative binomial regression, using sequencing depth as a model covariate to regress out the influence of technical effects, and Pearson residuals were used as the normalized and variance stabilized biological signal for downstream analysis.

Integration: Cells from each treatment condition and differentiation day were integrated in *Seurat* using the reciprocal principal component analysis-based approach, using the top 3000 variable features.

Dimensional reduction: PCA was applied to normalized and scaled data, and the top components (accounting for 90% of variance observed in the first 50 PCs) were used for UMAP embedding using RunUMAP(max_components = 2, n_neighbours = 50, min_dist = 01, metric = cosine) in *Seurat*.

Clustering: To identify clusters, we performed Louvain clustering in *Seurat* using the *FindClusters* function (resolution = 0.6).

Differential-expression analysis: Differential expression analyses were performed using the *wilcoxauc* function implemented in the *presto* package (version 1.0.0) (Korsunsky et al. 2019). Differentially expressed genes were ranked by the area under the receiver operating characteristic (AUROC) and the top markers for each cluster were used for annotation and visualization. Cell type-specific genes were visualized using the *DotPlot* function in *Seurat*.

Cell-type annotation: Cell clusters were annotated using a combined approach if *i*) marker-based annotation (Stem cells: *PTPRZ1*, *SOX2*; Primitive Streak: *MIXL1*, *CER1*; Paraxial Mesoderm: *TBX6*, *MSGN1*; Lateral Mesoderm: *HAND1*, *LRRC32*; Endoderm: *FOXA2*, *SOX17*), and *ii*) reference atlas-based label transfer. For label transfer, developmental scRNAseq data was used as a reference atlas to annotate scRNAseq data (i.e., query) in the current study (Pijuan-Sala et al. 2019; Tyser et al. 2021). Using *Seurat's* label transfer pipeline, we identified transfer anchors for each query-reference pair using FindTransferAnchors(…, normalization.method = "SCT", reference.reudction = "pca", dims = 1:50) and then mapped the query samples onto the reference atlas using MapQuery(…, reference.reduction = "pca", reduction.model = "umap"). The resulting prediction scores were cross-validated with cluster-specific markers obtained from differential-expression analyses to inform cluster annotation. In cases where many clusters annotated to a common lineage (e.g., endoderm), they were subclassified into subtypes (e.g., E1, E2 cell types).

Cell-type composition analysis: To compare the relative abundance of each lineage across treatment conditions, counts for each lineage were tallied within each treatment condition, and divided by the total number of cells profiled for that condition.

Differential abundance analysis: To evaluate regional differential abundances of cells in UMAP space across treatment conditions, we adopted the Milo method (Dann et al. 2022). In brief, for each comparison between the FZD2 and FZD7 agonist-treated conditions, cells from each condition were first resampled to normalize cell counts, and then a KNN graph representing higher-dimensional relationships between single cells was constructed. The KNN graph was then used to define neighborhoods of cells using the refined sampling scheme (Dann et al. 2022). Finally, the number of cells belonging to each condition within each neighborhood was counted and the differential abundance was computed using a negative binomial generalized linear model.

The differential abundance estimates for each neighborhood were visualized in UMAP space, with each node representing a given neighborhood (comprised of 20-80 cells each), and the color representing the differential abundance expressed as log fold-change (FZD7/FZD2). Non-significant differentials (FDR > 0.1) were truncated at zero.

Incucyte Live imaging and quantification

mCherry SP5 reporter lines were treated with different conditions for 5 days. Images were captured over time using Incucyte S3 Live-Cell Analysis System (Essen BioScience, USA). Data were acquired from a 20x objective lens in phase contrast and a red fluorescence channel (Excitation: 585, Emission: 665, acquisition time: 2000 ms). A total of four images per well were acquired at intervals. The mCherry Integrated Intensity per image (RCU x μm^2/Image) of each condition was calculated using the Incucyte S3 Live-Cell Analysis System software. Representative images on day 4 were shown.

Immunofluorescence

H1 hESCs treated with FLAgs and CHIR99021 at different doses were washed with cold PBS and then fixed for 20 min with 4% PFA. Fixed cells were rinsed with PBS, permeabilized with 0.3% triton for 10 min. and blocked with 1% BSA for 1 h. Cells were incubated for 2 hours with primary Abs for BRACHYURY, OCT4, SOX2, SOX17, TBX6, and HAND1 in 1% BSA (Table S3). Cells were washed 3 times with PBS and then stained for secondary antibody for 1 h at room temperature using Alexa Fluor 488-labeled donkey anti-goat, Alexa Fluor 568-labeled donkey anti-mouse Ab, Alexa Fluor 568-labeled donkey anti-rabbit or Alexa Fluor 647-labeled donkey anti-mouse. Coverslips were mounted using Fluoromount (Sigma-Aldrich) and analyzed on a Zeiss LSM700

confocal microscope using a 60X oil immersion objective. Images were assembled using ImageJ and Photoshop CS6 (Adobe Systems, Mountain View, CA).

Quantitative RT-PCR

Total RNA was extracted from H1 hESCs or WTC11 cells using TRIzol reagent (Invitrogen; Thermo Fisher Scientific, Inc., Waltham, MA, USA) according to the manufacturer's protocol and quantified using a nanodrop. Total RNA (1–2 μg) was reverse-transcribed into cDNA using the High-Capacity cDNA Reverse Transcription Kit (Applied Biosystems, Cat#4368813) according to the manufacturer’s protocol. qPCR was performed using the Power SYBR Green PCR Master Mix (Applied Biosystems, Cat# 4368708) using the following thermocycling conditions: 95 °C for 10 min, followed by 40 cycles of 95 °C for 15 sec., 60 °C for 1 min, and 72°C for 30 sec. Primer sequences indicated in Table S3 were used, and gene expression was evaluated by the $2^{-\Delta\Delta CT}$ method.

Luciferase reporter assay

HEK293T cells were transduced with lentivirus coding for the pBARls reporter (Biechele and Moon 2008) and with *Renilla* Luciferase as a control to generate a Wnt-βcatenin signaling reporter cell line. For luciferase assay, 2×10^5 cells were seeded in each well of 24-well plates for 24 hrs prior to stimulation. The following day, F2L6.13 or F7L6.13 protein were added, and following 16 hrs of stimulation, cells were lysed and luminescence was measured following the dual luciferase protocol (Promega) using an Envision plate reader (PerkinElmer).

Western blot

H1 ESCs were solubilized with lysis buffer (1% Nonidet P-40, 0.1% sodium dodecyl sulfate (SDS), 0.1% deoxycholic acid, 50 mM Tris (pH 7.4), 0.1 mM EGTA, 0.1 mM EDTA, 20 mM sodium fluoride (NaF), 1:500 protease inhibitors (Sigma) and 1 mM sodium orthovanadate (Na3VO4). Lysate was incubated for 30 min at 4 °C, centrifuged at 14,000 × g for 10 min, boiled in SDS sample buffer, separated by SDS-polyacrylamide gel electrophoresis, transferred onto a nitrocellulose membrane and Western blotted using the following Abs: BRACHYURY, SOX17, OCT4, GAPDH (Table S3). Ab detection was performed by a chemiluminescence-based detection system (ECL; ThermoFisher).

References

Biechele, Travis L., and Randall T. Moon. 2008. "Assaying Beta-Catenin/TCF Transcription with Beta-Catenin/TCF Transcription-Based Reporter Constructs." *Methods in Molecular Biology (Clifton, N.J.)* 468: 99–110.

Bray, Nicolas L., Harold Pimentel, Páll Melsted, and Lior Pachter. 2016. "Near-Optimal Probabilistic RNA-Seq Quantification." *Nature Biotechnology* 34 (5): 525–27.

Butler, Andrew, Paul Hoffman, Peter Smibert, Efthymia Papalexi, and Rahul Satija. 2018. "Integrating Single-Cell Transcriptomic Data across Different Conditions, Technologies, and Species." *Nature Biotechnology* 36 (5): 411–20.

Cao, Junyue, Malte Spielmann, Xiaojie Qiu, Xingfan Huang, Daniel M. Ibrahim, Andrew J. Hill, Fan Zhang, et al. 2019. "The Single-Cell Transcriptional Landscape of Mammalian Organogenesis." *Nature* 566 (7745): 496–502.

Dann, Emma, Neil C. Henderson, Sarah A. Teichmann, Michael D. Morgan, and John C. Marioni. 2022. "Differential Abundance Testing on Single-Cell Data Using k-Nearest Neighbor Graphs." *Nature Biotechnology* 40 (2): 245–53.

Hafemeister, Christoph, and Rahul Satija. 2019. "Normalization and Variance Stabilization of Single-Cell RNA-Seq Data Using Regularized Negative Binomial Regression." *Genome Biology* 20 (1): 296.

Hao, Yuhan, Stephanie Hao, Erica Andersen-Nissen, William M. Mauck 3rd, Shiwei Zheng, Andrew Butler, Maddie J. Lee, et al. 2021. "Integrated Analysis of Multimodal Single-Cell Data." *Cell* 184 (13): 3573-3587.e29.

Hart, Traver, Amy Hin Yan Tong, Katie Chan, Jolanda Van Leeuwen, Ashwin Seetharaman, Michael Aregger, Megha Chandrashekhar, et al. 2017. "Evaluation and Design of Genome-Wide CRISPR/SpCas9 Knockout Screens." *G3 (Bethesda, Md.)* 7 (8): 2719–27.

Korsunsky, Ilya, Nghia Millard, Jean Fan, Kamil Slowikowski, Fan Zhang, Kevin Wei, Yuriy Baglaenko, Michael Brenner, Po-Ru Loh, and Soumya Raychaudhuri. 2019. "Fast, Sensitive and Accurate Integration of Single-Cell Data with Harmony." *Nature Methods* 16 (12): 1289–96.

Loh, Kyle M., Angela Chen, Pang Wei Koh, Tianda Z. Deng, Rahul Sinha, Jonathan M. Tsai, Amira A. Barkal, et al. 2016. "Mapping the Pairwise Choices Leading from Pluripotency to Human Bone, Heart, and Other Mesoderm Cell Types." *Cell* 166 (2): 451–67.

Love, Michael I., Wolfgang Huber, and Simon Anders. 2014. "Moderated Estimation of Fold Change and Dispersion for RNA-Seq Data with DESeq2." *Genome Biology* 15 (12): 550.

Pijuan-Sala, Blanca, Jonathan A. Griffiths, Carolina Guibentif, Tom W. Hiscock, Wajid Jawaid, Fernando J. Calero-Nieto, Carla Mulas, et al. 2019. "A Single-Cell Molecular Map of Mouse Gastrulation and Early Organogenesis." *Nature* 566 (7745): 490–95.

Satija, Rahul, Jeffrey A. Farrell, David Gennert, Alexander F. Schier, and Aviv Regev. 2015. "Spatial Reconstruction of Single-Cell Gene Expression Data." *Nature Biotechnology* 33 (5): 495–502.

Slyper, Michal, Caroline B. M. Porter, Orr Ashenberg, Julia Waldman, Eugene Drokhlyansky, Isaac Wakiro, Christopher Smillie, et al. 2020. "A Single-Cell and Single-Nucleus RNA-Seq Toolbox for Fresh and Frozen Human Tumors." *Nature Medicine* 26 (5): 792–802.

Stuart, Tim, Andrew Butler, Paul Hoffman, Christoph Hafemeister, Efthymia Papalexi, William M. Mauck 3rd, Yuhan Hao, Marlon Stoeckius, Peter Smibert, and Rahul Satija. 2019. "Comprehensive Integration of Single-Cell Data." *Cell* 177 (7): 1888-1902.e21.

Subramanian, Aravind, Pablo Tamayo, Vamsi K. Mootha, Sayan Mukherjee, Benjamin L. Ebert, Michael A. Gillette, Amanda Paulovich, et al. 2005. “Gene Set Enrichment Analysis: A Knowledge-Based Approach for Interpreting Genome-Wide Expression Profiles.” *Proceedings of the National Academy of Sciences of the United States of America* 102 (43): 15545–50.

Tian, Ruilin, Mariam A. Gachechiladze, Connor H. Ludwig, Matthew T. Laurie, Jason Y. Hong, Diane Nathaniel, Anika V. Prabhu, et al. 2019. “CRISPR Interference-Based Platform for Multimodal Genetic Screens in Human IPSC-Derived Neurons.” *Neuron* 104 (2): 239-255.e12.

Tyser, Richard C. V., Elmir Mahammadov, Shota Nakanoh, Ludovic Vallier, Antonio Scialdone, and Shankar Srinivas. 2021. “Single-Cell Transcriptomic Characterization of a Gastrulating Human Embryo.” *Nature* 600 (7888): 285–89.

Welker, Jordan M., Wesley A. Wierson, Maira P. Almeida, Carla M. Mann, Melanie E. Torrie, Zhitao Ming, Stephen C. Ekker, et al. 2021. “GeneWeld: Efficient Targeted Integration Directed by Short Homology in Zebrafish.” *Bio-Protocol* 11 (14): e4100.

Wierson, Wesley A., Jordan M. Welker, Maira P. Almeida, Carla M. Mann, Dennis A. Webster, Melanie E. Torrie, Trevor J. Weiss, et al. 2020. “Efficient Targeted Integration Directed by Short Homology in Zebrafish and Mammalian Cells.” *ELife* 9 (May). https://doi.org/10.7554/eLife.53968.
